# Remote stereoselective deconjugation of α,β-unsaturated esters by simple amidation reactions†
†Electronic supplementary information (ESI) available. CCDC 1034974, 1034976–1034978. For ESI and crystallographic data in CIF or other electronic format see DOI: 10.1039/c5sc01118c



**DOI:** 10.1039/c5sc01118c

**Published:** 2015-05-25

**Authors:** Mahesh Vishe, Radim Hrdina, Amalia I. Poblador-Bahamonde, Céline Besnard, Laure Guénée, Thomas Bürgi, Jérôme Lacour

**Affiliations:** a Department of Organic Chemistry , University of Geneva , Quai Ernest Ansermet 30 , CH-1211 Geneva 4 , Switzerland . Email: jerome.lacour@unige.ch ; http://www.unige.ch/sciences/chiorg/lacour/ ; Fax: +41 22-379-3215; b Laboratory of Crystallography , University of Geneva , Quai Ernest Ansermet 24 , CH-1211 Geneva 4 , Switzerland; c Department of Physical Chemistry , University of Geneva , Quai Ernest Ansermet 30 , CH-1211 Geneva 4 , Switzerland

## Abstract

The thermodynamically disfavored isomerization of α,β-unsaturated esters to deconjugated β,γ-unsaturated analogues occurs readily when coupled to an amidation. Within the framework of macrocyclic derivatives, it is shown that 15, 16, and 18 membered macrocycles react with *t*BuOK and anilines to generate, in one-pot, β,γ-unsaturated amides (yields up to 88%). Importantly, single (chiral) diastereomers are isolated (d.r. > 49 : 1, ^1^H NMR) irrespective of the size and nature of the rings, showing an effective transmission of remote stereochemistry during the isomerization process. CSP-chromatographic resolution and absolute configuration determination by VCD are achieved.

## Abstract 2

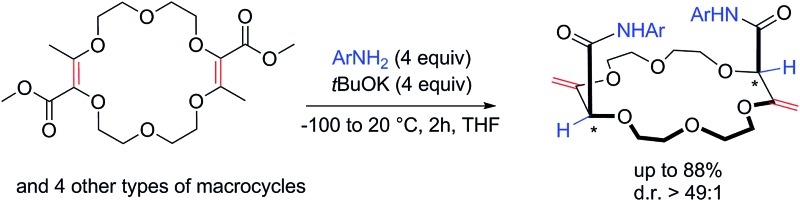
The amidation of macrocyclic conjugated esters affords in one-pot single (chiral) β,γ-unsaturated diastereomers *via* effective remote stereocontrol.

## Introduction

**Scheme 1:**
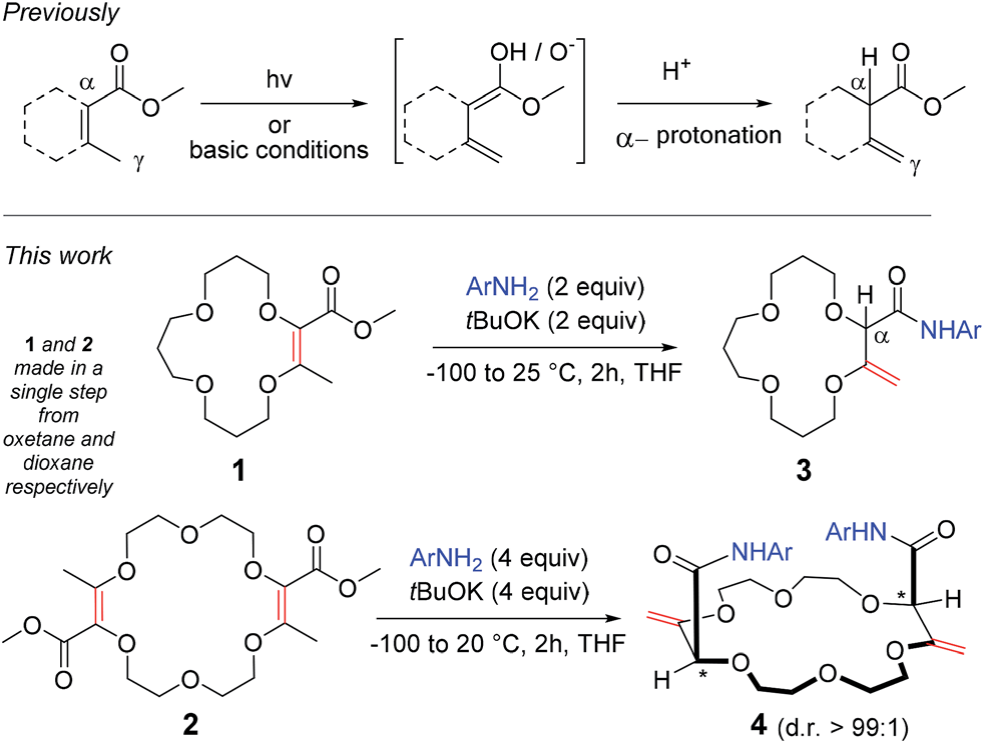
Deconjugation of 2,3-dioxo-methyl butenoate moieties.

The isomerization of α,β-unsaturated esters to their deconjugated β,γ-unsaturated analogues is a valued reaction as it allows, in a single step, alkenes and esters to be decoupled leading to benefits from the individualized reactivity and properties of the separated functional groups. However, due to the thermodynamic stability associated with conjugation, achieving such an isomerization is intrinsically difficult. To solve this problem, photochemical^1^ or strongly basic conditions^2^ are used to generate dienol or dienolate intermediates, which are then protonated selectively in the α position to the ester, under kinetic control (Scheme 1, top).^3^ Such reactions have been used in both academic and industrial laboratories.^2b,4^ Herein, we report that the isomerization becomes facile when coupled to an amidation reaction. Using unsaturated macrocycles as substrates for practical reasons,^5^ we report that the 15 and 18-membered derivatives **1** and **2** react with *t*BuOK and anilines to generate, in one-pot, the β,γ-unsaturated amides **3** and **4** respectively (Scheme 1, bottom, yields up to 88%). In the case of **4**, even if the stereogenic centers are separated by more than 7 atoms, single diastereomers are observed (d.r. > 49 : 1, ^1^H NMR).^6,7^ This highly stereoselective isomerization can be extended to other ring compositions and sizes showing the effectiveness of the remote (intramolecular) transmission of stereochemistry. Calculations at the DFT/B3PW91 level validate the preferred formation of the chiral diastereomeric series over the meso. CSP-chromatographic separation of the enantiomers was also achieved and the absolute configuration determined by VCD.

## Results and discussion

Recently, we an others^5,8^ have reported the synthesis of functionalized polyether macrocycles through metal-catalyzed decompositions of α-diazo β-ketoesters in the presence of cyclic ethers such as oxetane and 1,4-dioxane. In these one pot reactions that occur at high concentration (typically 1 M), four building blocks are condensed. Compounds **1** and **2**, which possess respectively one and two dioxobutanoate motifs, are afforded in generally good yields (up to 84%).^5^ The presence of both α,β-unsaturated ester moieties and β-methyl groups led us to consider the possibility of base or photochemically-induced alkene isomerizations. Unfortunately, attempts following established protocols^2*b*^ led either to unreacted starting materials or to the complete decomposition of the substrates.

**Fig. 1:**
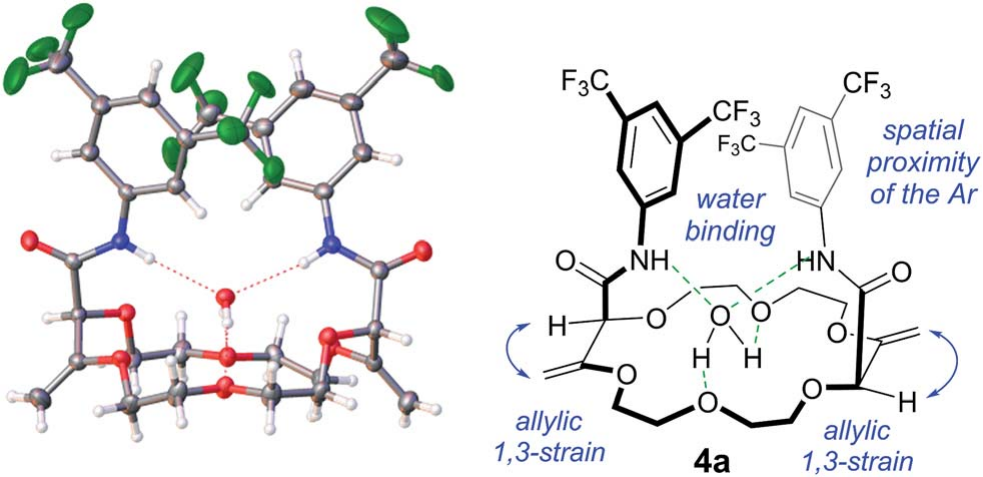
“Diaxial”-like conformation of the isomerized macrocycles, *e.g.* **4a**. Displacement ellipsoid plot of the crystal structure of **4a**. Thermal ellipsoids are drawn at 50% probability. Solvent molecules and the disorder of the CF_3_ groups are omitted for clarity.

However, treatments of **1** and **2** with *t*BuOK at 20 °C in the presence of 3,5-bis(trifluoromethyl) aniline (**a**, two and four equivalents respectively),^9^ led to products **3a** and **4a** after 2 hours of reaction (70 and 60% yield respectively). In both cases, ^1^H NMR spectroscopic analysis indicated the presence of amide groups and, importantly, displayed signals typical for enol ether protons (*δ* 4.33–4.35 ppm and 4.33–4.51 ppm respectively) and also singlets which could be assigned to α-hydrogen atoms (*δ* 4.27 and 4.40 ppm for **3** and **4** respectively). Interestingly, in the case of **4a**, only one set of signals was obtained, indicative of the presence of a single diastereomeric species despite a separation of the stereogenic centers by more than 7 atoms (d.r. > 49 : 1, ^1^H NMR)^10^ – the nature and the relative (chiral) configuration of which was established unambiguously by X-ray diffraction analysis (ESI† and Fig. 1).^11^

With this result in hand, an optimization study was conducted using 3,5-bis(trifluoromethyl) aniline (**a**) for the amidation (Table 1). A 1 : 1 ratio between the base and the aniline was strictly maintained throughout the study. With both substrates **1** and **2**, the addition of the base at 20 °C leads to an exothermic reaction (entries 1 and 4). Care was then taken to add *t*BuOK at a low temperature (–100 °C) and then allow the reaction to warm up to 20 °C on its own. Satisfactorily, yields of products increased in both cases (entries 2 and 5).

**Table 1:** Optimization studies

Entry	Substrate	Base	Equiv.	Temp (°C)	Yield^*b*^	d.r.
1	**1**	*t*BuOK	2	20	70	—
2^*a*^	**1**	*t*BuOK	2	–100	80	—
3^*a*^	**1**	*t*BuOK	4	–100	95	—
4	**2**	*t*BuOK	4	20	60	>49
5^*a*^	**2**	*t*BuOK	4	–100	85	>49
6^*a*^	**2**	*t*BuOK	8	–100	85	>49
7^*a*^	**2**	*t*BuONa	4	–100	83	>49
8^*a*^	**2**	*t*BuOLi	4	–100	—	—
9^*a*^	**2**	KHMDS	4	–100	60	>49
10^*a*^	**2**	NaHMDS	4	–100	35	>49
11^*a*^	**2**	LiHMDS	4	–100	—	—
12^*a*^	**2**	BuLi	4	–100	—	—
13^*a*^	**2**	LDA	4	–100	35	>49

^*a*^Addition of base performed at –100 °C (using an ethanol liquid nitrogen bath) and, after 2 minutes, the reaction was allowed to warm to 20 °C.

^*b*^Isolated yields. Average of at least two runs [%].

Addition to **1** and **2** of a larger amount of base and aniline (four and eight equivalents respectively) was beneficial only in the case of monoester **1** (entry 3). A stoichiometry of two equivalents of base and aniline for each unsaturated ester moiety was thus chosen for the remainder of the study.

**Fig. 2:**
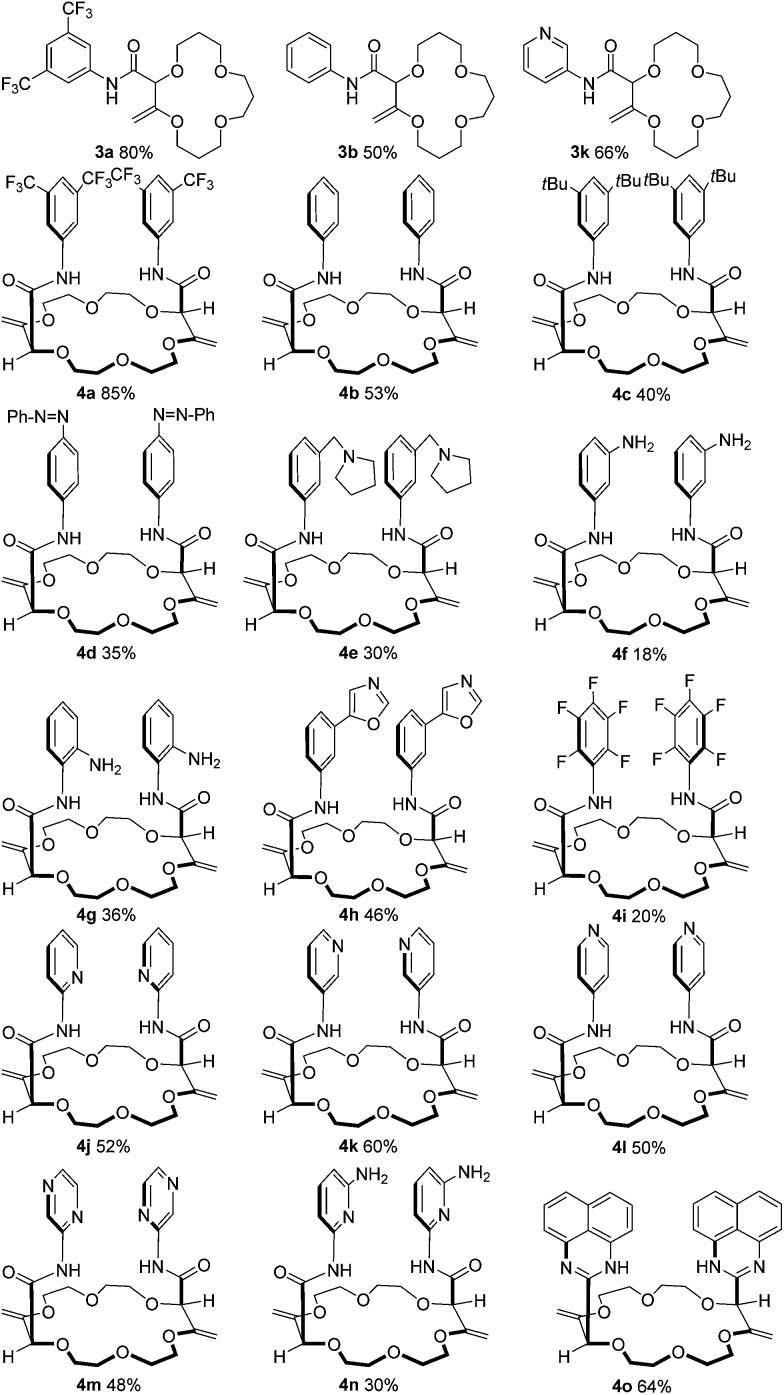
Substrate scope using macrocycles **1** and **2**.

Care was also taken to investigate the influence of the base and its counterion as, with crown-ether like compounds, an influence of the cationic atom was expected. While *t*BuONa afforded **4a** in a similar yield (83 *vs.* 85%), a total lack of product was noticed with *t*BuOLi (entry 8). With hexamethyldisilazane salts (entries 9 to 11), lower yields were globally obtained and a clear difference was noticed between the potassium, sodium and lithium derivatives. Again, no product was formed with the Li^+^ salt; a result confirmed with BuLi (entry 12). Only in the case of LDA (entry 13) was a modest yield of **4a** obtained. In view of these results, *t*BuOK was selected and the reaction was generalized with a series of anilines as reactants (Fig. 2).

First, **1** was reacted with regular aniline **b** and pyridine-derived **k** to afford the corresponding 15C4 products **3b** and **3k** in moderate yields (50% and 66% respectively). Using **2** as the substrate, 18C6 products **4a–4o** were obtained in low to excellent yields (18–85%). Electron-donating and withdrawing substituents were equally introduced at the *ortho*, *meta* and *para* positions of the anilines (products **4c–4i**). Amino pyridines and pyrazines (**j–n**) were well tolerated as products **4j**, **4k**, **4l**, **4m** and **4n** were isolated in 30 to 60% yields. Finally, when 1,8-diaminonaphthalene was used with **2** as the substrate, a double sequence of three consecutive reactions occurred to afford product **4o** in 64% yield. In this case, the resulting amides react spontaneously with the free amino naphthalene groups to form cyclic perimidines under the reaction conditions.

**Fig. 3:**
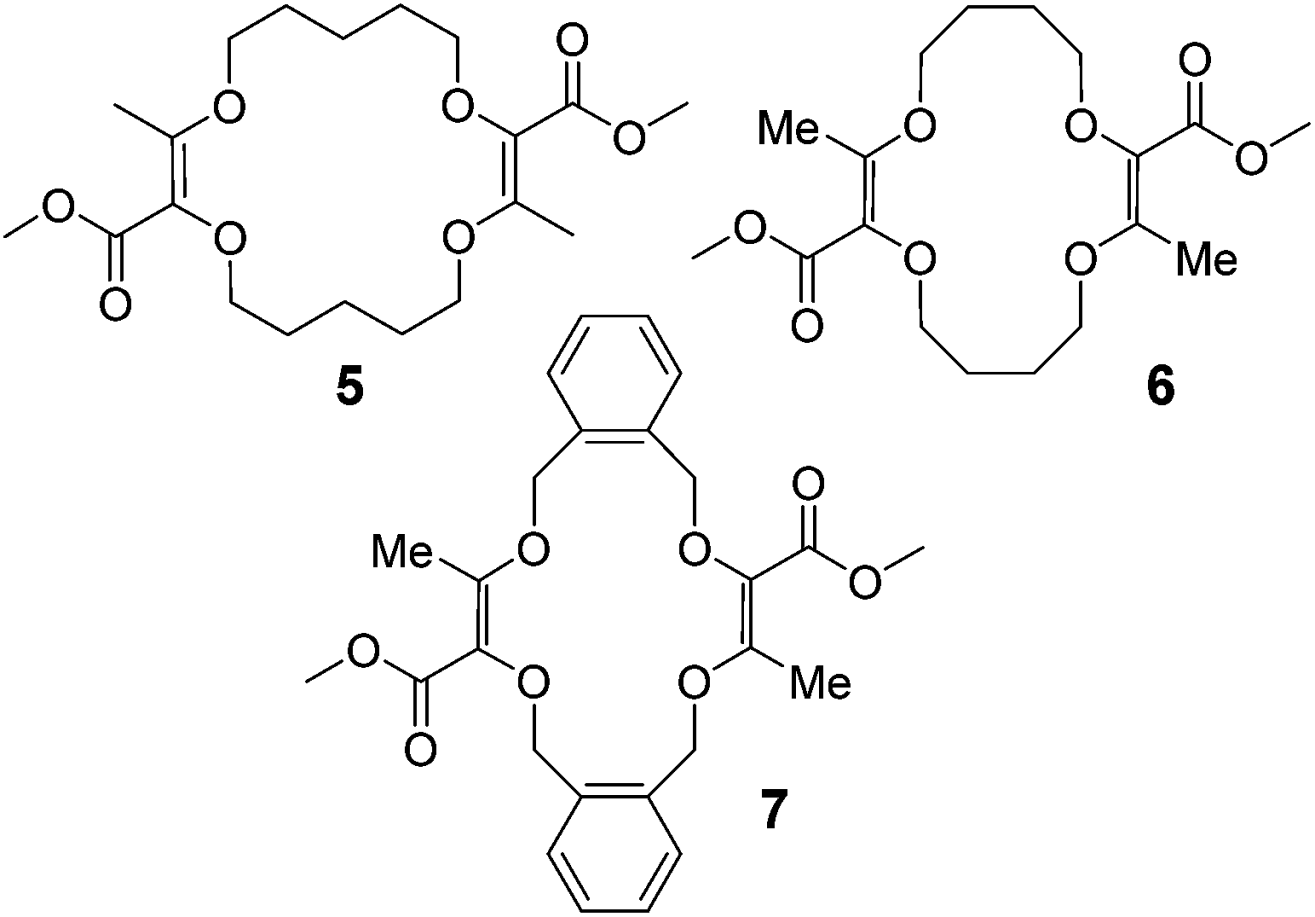
THP, THF and benzofuran-derived macrocycles **5**, **6** and **7**.

**Fig. 4:**
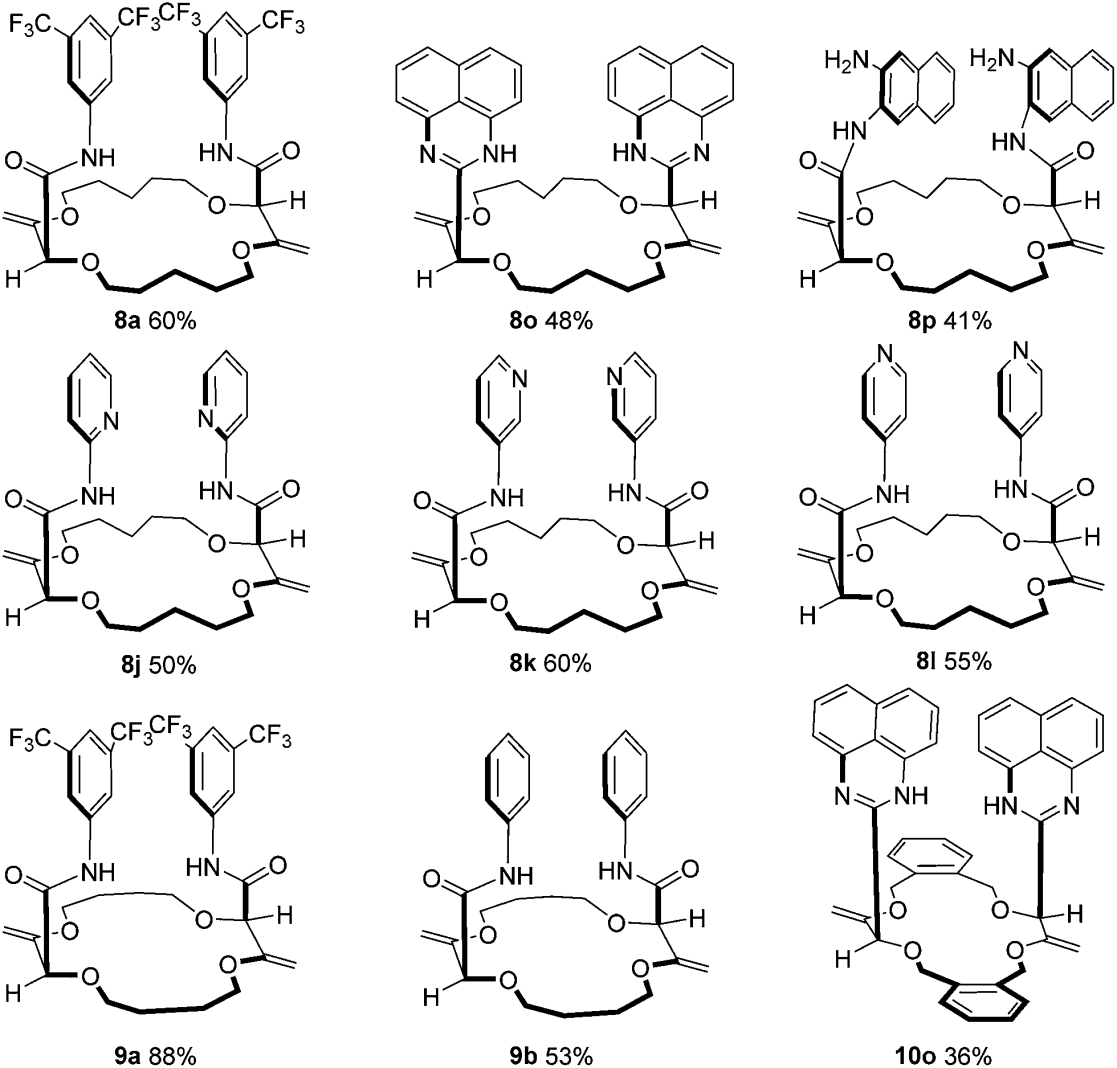
Substrate scope using macrocycles **5**, **6** and **7**.

Moreover, it was shown that macrocycles **5**, **6**, **7** (Fig. 3), derived in one-step from THP (tetrahydropyran), THF (tetrahydrofuran) and benzofuran,^5a,c^ also reacted well to afford compounds of type **8**, **9** and **10** respectively. A selection of products is detailed in Fig. 4 using some of the anilines or pyridines already introduced on **3** and **4**. Similar yields were globally obtained for these 18C4 and 16C4 macrocycles demonstrating the generality of the process.

Several of these macrocycles were analyzed successfully by X-ray diffraction (see Fig. 1 (**4a**) and the ESI† for **4l**, **8a**, and **10o**). Of importance, strong evidence was found for allylic 1,3-strain interactions between the exocyclic double bonds and the adjacent stereogenic centers.^12^ In fact, in all macrocycles, irrespective of their size, the nature of the ring and of the substituents, the α-C–H bonds are periplanar to the exocyclic C=CH_2_ bonds.^13^ As a consequence, the amide moieties are disposed in an essentially perpendicular orientation to the mean plane of the macrocycles. This geometry then favors (i) close interactions between the aromatic moieties and (ii) hydrogen bonding interactions between the amides N–H and neighboring oxygen atoms or, in the case of the 18C6 macrocycles of type **4**, with guest water molecules.^14^

## Mechanistic rationale

**Scheme 2:**
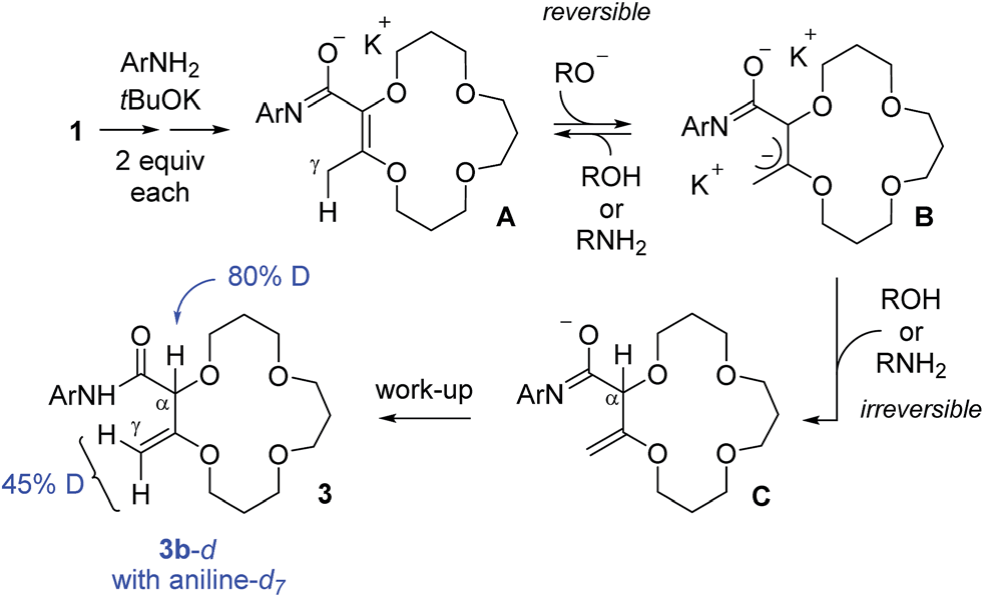
Condensed mechanistic rationale for the tandem amidation–isomerization. Results of deuteration experiments are indicated in blue.

To gain some insight on the transformation, two isotope labeling experiments were performed. First, compound **1** was treated with aniline-*d*_7_ and *t*BuOK to yield deuterated products **3b** *-d* with 80% and 45% incorporations of deuterium at the α and γ positions respectively (see Scheme 2). This result indicates that the aniline moiety acts both as a nucleophile and proton source in the transformation. The resulting **3b** *-d* were then resubmitted to the reaction conditions but with regular aniline PhNH_2_ in the medium. After two hours, the amount of deuteration was found to be unchanged, indicating a lack of D/H exchange in compound **3b** *-d*. With this experimental information, a mechanistic rationale can be proposed (Scheme 2).

First, a reaction between the anilines and *t*BuOK occurs to generate anilides that react both as a nucleophile and base to yield, in a few steps, anionic intermediates of type **A**. γ-Deprotonation then occurs to form allylic **B**; this step being reversible to account for the 45% of deuteration at the γ position of **3b** *-d* in the presence of aniline-*d*_7_. However, α-protonation of **B** also occurs. This step is proposed to be irreversible to explain the lack of D/H exchange when **3b** *-d* is resubmitted to the reaction conditions and this explains the predominant formation of the deconjugated products.^15,16^ Imidate intermediates **C** are then quenched during the work-up, yielding products **3**.

**Scheme 3:**
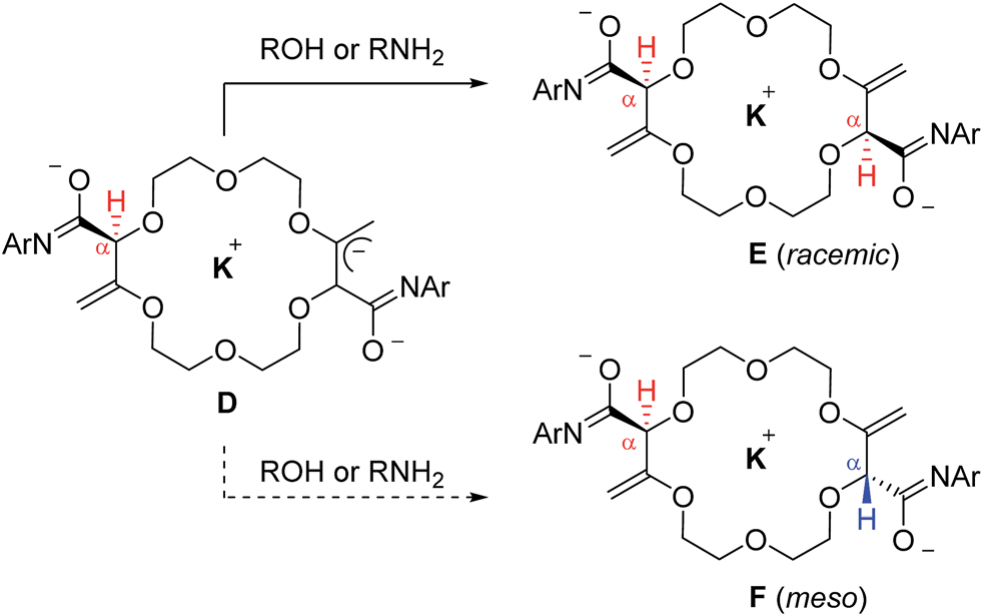
Stereoselective protonation in favor of the racemic over meso diastereomers.

**Fig. 5:**
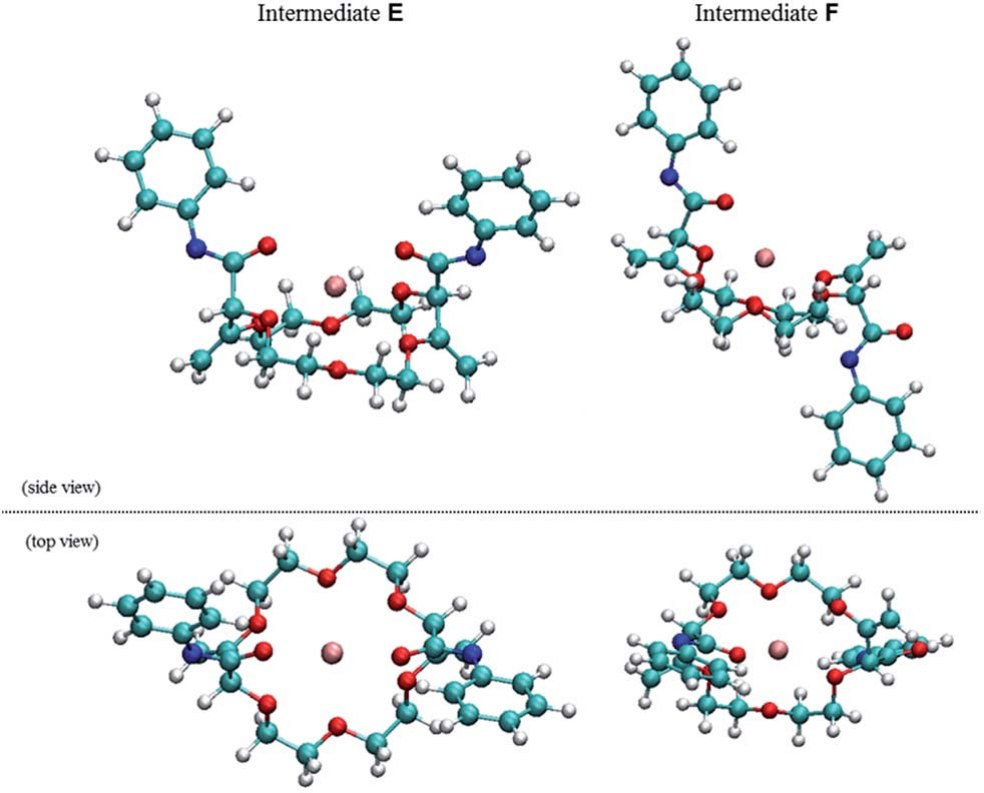
Side and top view of the calculated geometries (DFT/B3PW91) of racemic **E** (right) and meso **F** (left, +5.4 kcal mol^–1^) intermediates.

With substrates, **2**, **5**, **6** and **7** that contain two butenoate motifs, this isomerization sequence occurs twice to generate compounds **4**, **8**, **9** and **10** with a very high stereoselectivity (d.r. > 49 : 1, ^1^H NMR) even if the stereogenic centers are separated by several atoms (seven or more).^6^ A rationale for this general asymmetric induction is detailed in Scheme 3 and Fig. 5. In intermediates **D**, which possess an already transposed double bond and an anionic allylic imidate functional group, the presence of the first stereogenic center controls remotely the stereoselectivity of the protonation – and hence the configuration of the second center. It is proposed that a potassium (or sodium) cation serves as a bridge (relay) between the two imidate anions and, under these chelating conditions, the pathway leading the racemic product is highly favored.

Full optimization of structures **E** and **F** were conducted at the DFT/B3PW91 level of theory to assess the coordination mode of the potassium cation and the relative energy of both diastereomers. Care was taken to account for the presence of the two imidate moieties that allow a coordination of the potassium cation with either oxygen or nitrogen atoms (see ESI†). Geometry optimizations for all possible conformers of **E** and **F** were thus modeled. Only intermediate **E** favors the chelation of the cation with both oxygens of the imidate moieties.^17^ The comparison of the relative energies of the most stable conformers for intermediates **E** (racemic, Ar = Ph) and **F** (meso, Ar = Ph) indicates that the chiral diastereomer is more stable that its achiral analogue by 5.4 kcal mol^–1^ (Fig. 5).^18^ This energy difference between **E** and **F** is sufficiently large to consider with confidence that there is also a large energy gap between the transition states which leads to these intermediates; this ΔΔ*G* ^‡^ gap is decisive for the stereoselectivity as the α-protonation is irreversible (see above).^19^

**Fig. 6:**
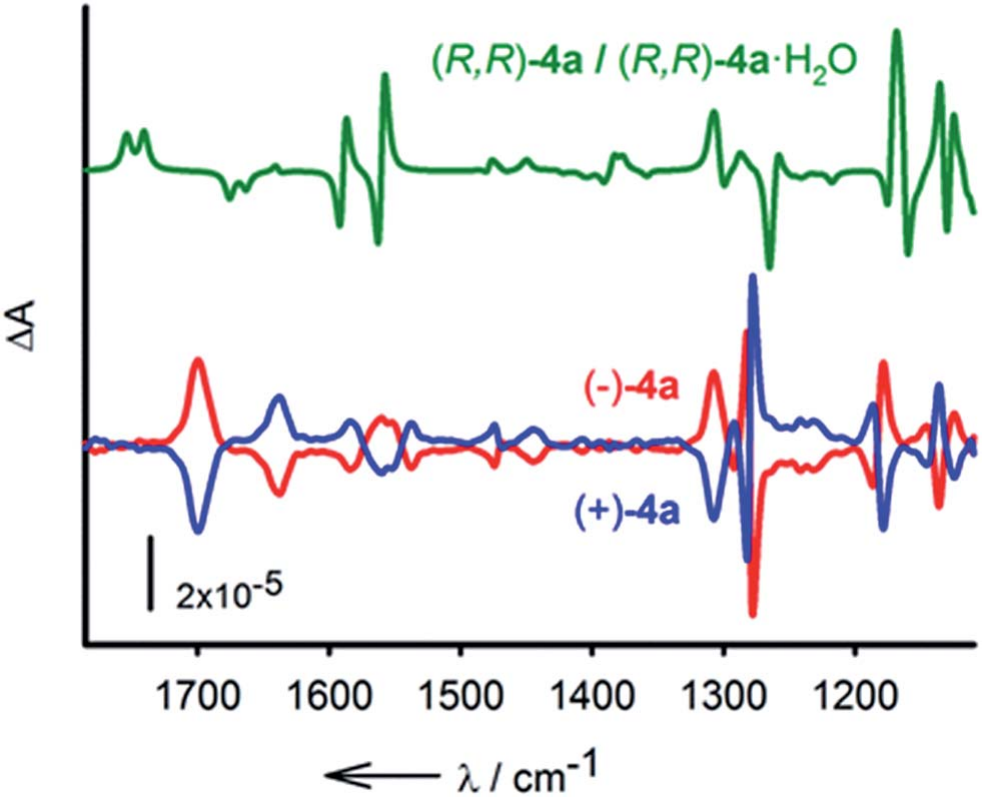
Calculated (1 : 1 average) spectrum of (*R*,*R*)-**4a** and (*R*,*R*)-**4a**·H_2_O (green). Experimental VCD spectra (CD_2_Cl_2_, 298 K) of (–)-**4a** (red) and (+)-**4a** (blue).

Finally, the resolution of **4a** was tackled.^20^ The enantiomeric separation was performed by CSP-chromatography on a semi-preparative scale using a Chiralpak IC column and a mixture of *n*-hexane : i-PrOH 85 : 15 as the eluent. From 50 mg of *rac*-**4a**, after several runs, two major separated fractions were afforded, 20 mg (ee > 99%) of (–)-**4a** and 18 mg (ee > 99%) of (+)-**4a** (see the ESI† for details). The electronic circular dichroism (ECD) spectra display totally symmetric curves in the 250 nm to 300 nm domain. The spectra are reported in Fig. S4.† With the separated enantiomers in hand, care was taken to determine the absolute configuration with certainty.^21^ This was established by vibrational circular dichroism (VCD) in view of the rigidity of compounds **2**.^22^ IR absorption and VCD spectra were measured for solutions (CD_2_Cl_2_) of both (+) and (–)-**4a** and compared to the averaged spectrum calculated for (*R*,*R*)-**4a** and its water complex (*R*,*R*)-**4a**·H_2_O (Fig. 6). Overall, considering that the macrocycle is present in solution as a mixture of the hydrated and non-hydrated forms, a good agreement between the experimental and theoretical spectra was observed. Some discrepancies are observed in the region of the amide II vibrations (N–H deformation) around 1560 cm^–1^. This spectral region is strongly affected by the interaction with water, which is not well described by the calculations. Despite this difficulty the VCD measurements allow the assignment of (–)-**4a** as the (*R*,*R*) enantiomer.

## Conclusions

In conclusion, a new deconjugation reactivity of α,β-unsaturated esters is reported owing to an amidation–isomerization sequence. This transformation is favored by the presence of anilines that act not only as nucleophiles but also, importantly, as effective proton sources in the isomerization step. Applied to macrocycles, the process is highly stereoselective (d.r. > 49 : 1) despite a separation of the stereogenic centers by more than 7 atoms. Calculations at the DFT/B3PW91 level validate the preference for the chiral diastereomers *via* a cation acting as a relay for the stereochemical transmission. CSP-chromatographic separation of the enantiomers was also achieved and the absolute configuration determined by VCD. As such, well-defined conformationally-constrained chiral macrocycles are obtained – and this in only two steps from simple and commercial cyclic ether precursors. Applications in enantioselective catalysis are looked for.^23^
