# Supplementary material for: Remote stereoselective deconjugation of α,β-unsaturated esters by simple amidation reactions
Source: Chem Sci. 2015 May 25;6(8):4923–8. doi: 10.1039/c5sc01118c (PMC5664367; doi:10.1039/c5sc01118c)
Supplement: Supplementary file 1 [file SC-006-C5SC01118C-s001.pdf]

Electronic Supplementary Material (ESI) for Chemical Science.
This journal is © The Royal Society of Chemistry 2015

Remote Stereoselective Deconjugation of α,β-Unsaturated Esters by Simple Amidation Reactions

Mahesh Vishe,[a] Radim Hrdina,[a] Amalia I. Poblador-Bahamonde,[a] Céline Besnard,[b] Laure Guénée,[b] Thomas Bürgi,[c] and Jérôme Lacour[*,a]*

[a] *Department of Organic Chemistry, University of Geneva, 30 Quai Ernest Ansermet, CH-1211 Geneva 4, Switzerland*
[b] *Laboratory of Crystallography, University of Geneva, 24 Quai Ernest Ansermet, CH-1211 Geneva 4, Switzerland*
[c] *Department of physical Chemistry, University of Geneva, 30 Quai Ernest Ansermet, CH-1211 Geneva 4, Switzerland*

Jerome.Lacour@unige.ch

Supporting Information

Table of Contents

1. General Remarks 2
2. General procedures 2
 2.a. General synthesis of macrocycles 2
 2.b. General synthesis of perimidine derivatives 2
3. Spectral and spectrometric data 3
4. Enantiodifferentiation of 4a using $Eu(hfc)_3$ as NMR chiral solvating agent (^{1}H NMR, 400 MHz, $CDCl_3$) 57
5. CSP-HPLC traces for 4a 58
6. ECD spectra of 4a 59
7. Computational Details. 60
8. Vibrational circular dichroism (VCD) and infrared (IR) analysis 67
9. Crystallographic data 70
 Macrocycle 4a 70
 Macrocycle 4l 71
 Macrocycle 8a 72
 Macrocycle 10o 73
10. References 74

1. General Remarks

NMR spectra were recorded on 300, 400 or 500 MHz spectrometer at 20 °C unless otherwise stated. ^{1}H-NMR chemical shifts are given in ppm relative to Me_4Si with the solvent resonance used as the internal standard ($CDCl_3$ δ = 7.26 ppm). ^{13}C-NMR (125 or 101 MHz) chemical shifts were given in ppm relative to Me_4Si with the solvent resonance used as the internal standard ($CDCl_3$ = 77.16 ppm). IR spectra were recorded using an ATR sampler and are reported in wave numbers (cm^{-1}). Melting points (Mp) were measured in open capillary tubes and were uncorrected. Optical rotations were measured in a thermostated (20 °C) 10.0 cm long microcell at 589 nm (Na). Electrospray mass spectra (ESI +) were obtained by the department of Mass Spectrometry of the University of Geneva. All reactions involving air sensitive compounds were carried out under dry N_2 or argon by means of an inert gas/vacuum double manifold line and standard Schlenk techniques. Flash column chromatography was performed with silica gel 40-63 or alumina (neutral Brockmann I, 50-200 μm).

2. General procedures

2.a. General synthesis of macrocycles

To the suspension of starting macrocycle **1** (0.1 mmol) and aniline (0.4 mmol) in 1.0 mL of dry THF was added *t*BuOK (44.8 mg, 0.4 mmol) at -100 °C. After 2 minutes, the cooling bath (EtOH, N_2 liquid) was removed and the reaction was allowed to reach room temperature on its own. It was stirred for additional 2 hours. Without further treatment or work-up, the reaction mixture was purified by column chromatography (SiO_2) or preparative TLC.

2.b. General synthesis of perimidine derivatives

To the suspension of starting macrocycle (**2o**, **5o** or **7o**) (0.1 mmol), 1,8-diaminonaphthalene (0.22 mmol) and *t*-BuOH (0.1 mmol) in 1.0 mL of dry THF was added *t*BuOK (44.8 mg, 0.44 mmol) at -100 °C. After 2 minutes, the cooling bath (EtOH, N_2 liquid) was removed and the reaction was allowed to reach room temperature on its own. It was stirred for additional 2 hours. Without further treatment or work-up, the reaction mixture was purified by column chromatography (SiO_2) or preparative TLC.

3. Spectral and spectrometric data

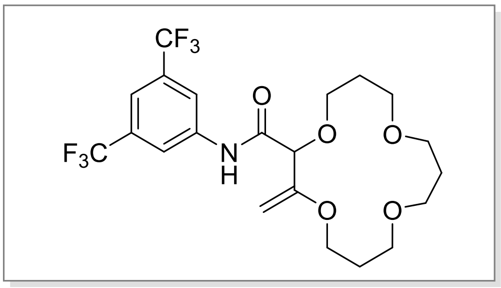

3a. Yield : 80% of pale yellow solid (54 mg)

M.p. 128 °C - 130 °C (crystallized from CH_2Cl_2 / Pentane)

R*f* : 0.31 (silica gel, mobile phase EtOAc/ Pentane 30/70),

^{1}H NMR (400 MHz, $CDCl_3$): δ/ppm = 1.75– 1.93 (m, 5H), 2.00 – 2.10 (m, 1H), 3.49 – 3.75 (m, 9H), 3.81 – 3.89 (m, 3H), 4.27 (s, 1H), 4.33 (d, *J* = 2.7 Hz, 1H), 4.35 (d, *J* = 2.6 Hz, 1H), 7.61 (s, 1H), 8.22 (s, 2H), 9.25 (s, 1H).

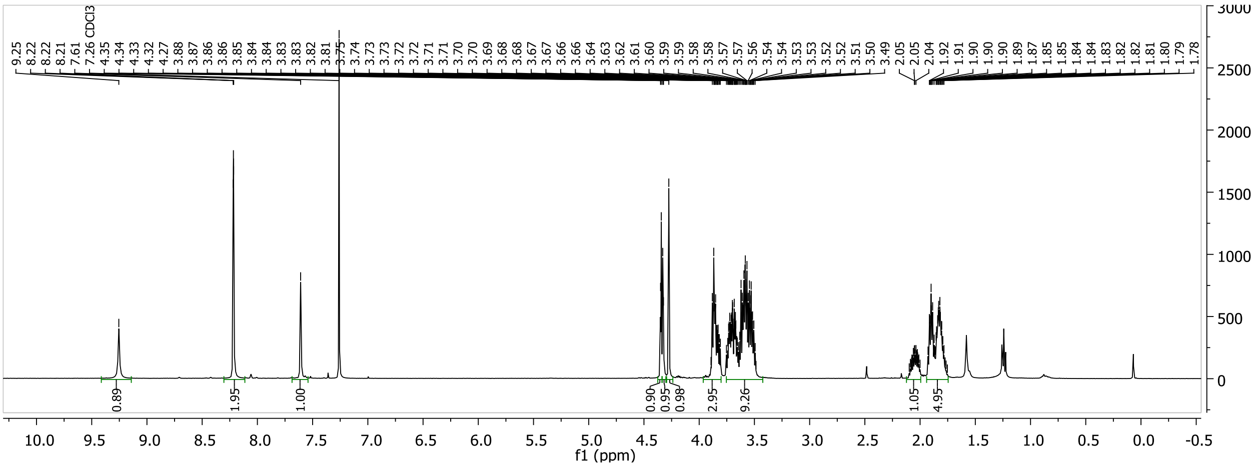

^{13}C NMR (101 MHz, $CDCl_3$): δ/ppm = 29.86 (1CH_2), 29.83 (1CH_2), 30.55 (1CH_2), 64.67 (1CH_2), 66.73 (2CH_2), 67.12 (1CH_2), 68.44 (1CH_2), 68.64 (1CH_2), 82.88 (1CH), 88.52 (1CH_2), 117.62 (m, 1CH), 119.43 (m, 2CH), 123.29 (q, *J* = 272.8 Hz, 2C), 132.48 (q, *J* = 33.6 Hz, 2C), 139.24 (1C), 157.50 (1C), 168.63 (1C).

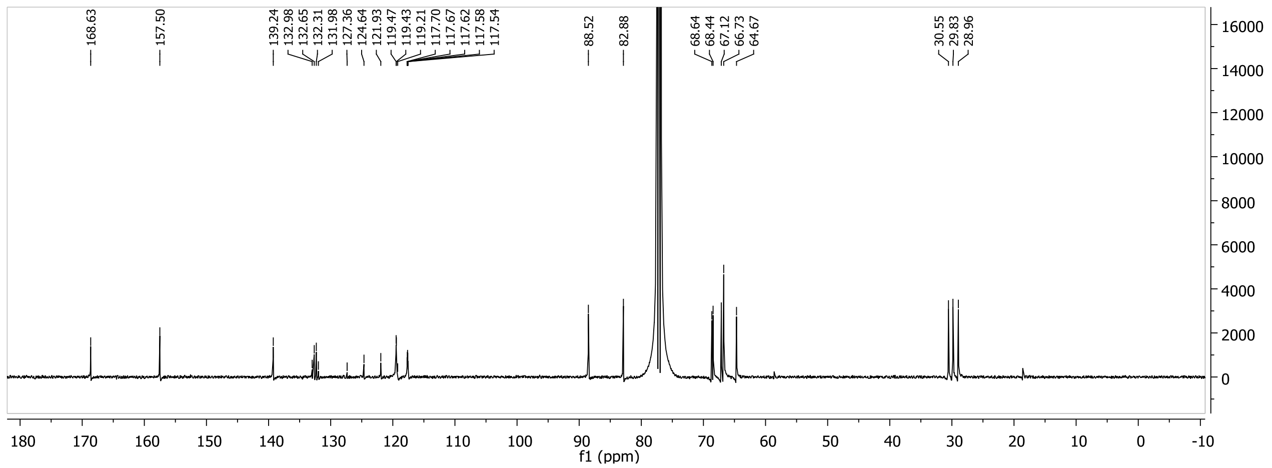

^{19}F NMR (282 MHz, $CDCl_3$): δ/ppm = -62.29 (s, 6F).

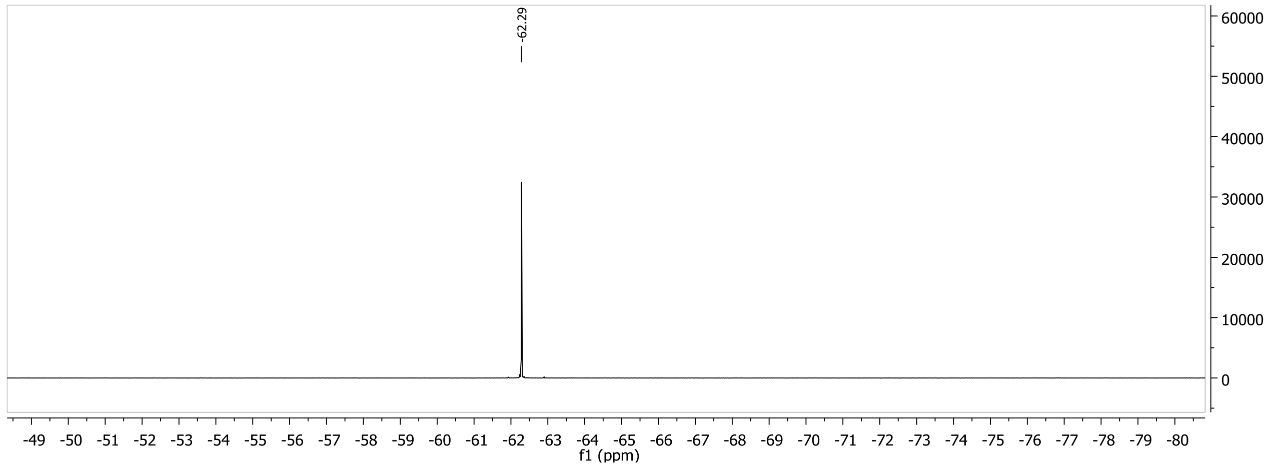

IR (neat): $\tilde{\nu}$ /cm^{-1} = 3232, 2935, 2864, 1679, 1622, 1535, 1474, 1443, 1379, 1330, 1274, 1167, 1121, 1082, 1008, 1032, 933, 882, 762, 699, 680, 568.

HR-ESI: m/z = 486.1715 $[M+H]^+$ (calculated for $C_{21}H_{26}F_6NO_5$ m/z = 486.1710)

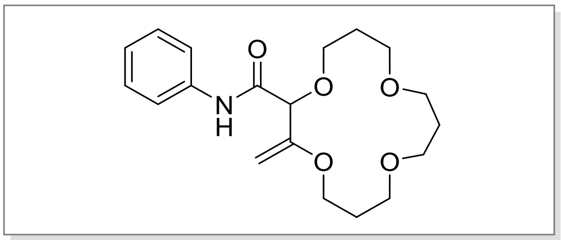

3b. Yield : 50% of pale yellow non crystalline solid (30 mg)

R*f* : 0.17 (silica gel, mobile phase EtOAc/ Pentane 30/70)

^{1}H NMR (400 MHz, $CDCl_3$): δ/ppm = 1.65 – 1.93 (m, 6H), 3.41 – 3.64 (m, 9H), 3.69 – 3.81 (m, 3H), 4.18 (s, 1H), 4.23 (d, J = 2.6 Hz, 1H), 4.25 (d, J = 2.6 Hz, 1H), 7.03– 7.08 (m, 1H), 7.24 – 7.29 (m, 2H), 7.55 – 7.56 (m, 2H), 8.62 (s, 1H).

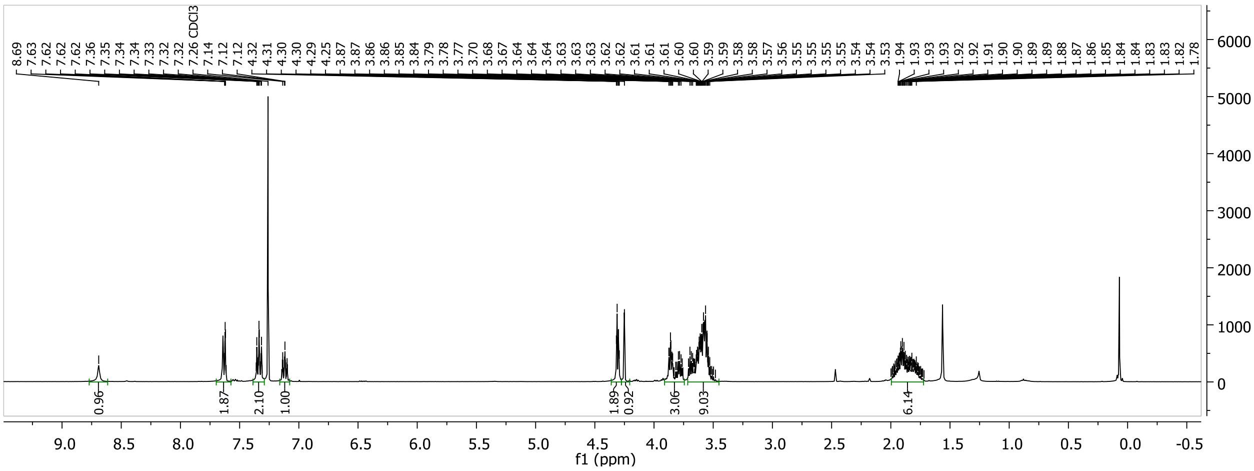

^{13}C NMR (101 MHz, $CDCl_3$): δ/ppm = 29.15 ($1CH_2$), 30.41 ($1CH_2$), 30.55 ($1CH_2$), 63.83 ($1CH_2$), 66.62 ($1CH_2$), 66.70 ($1CH_2$), 66.67 ($1CH_2$), 67.52 ($1CH_2$), 67.86 ($1CH_2$), 82.74 (1CH), 87.80 ($1CH_2$), 119.75 (2CH), 124.43 (1CH), 129.10 (2CH), 137.64 (1C), 158.21 (1C), 167.51 (1C).

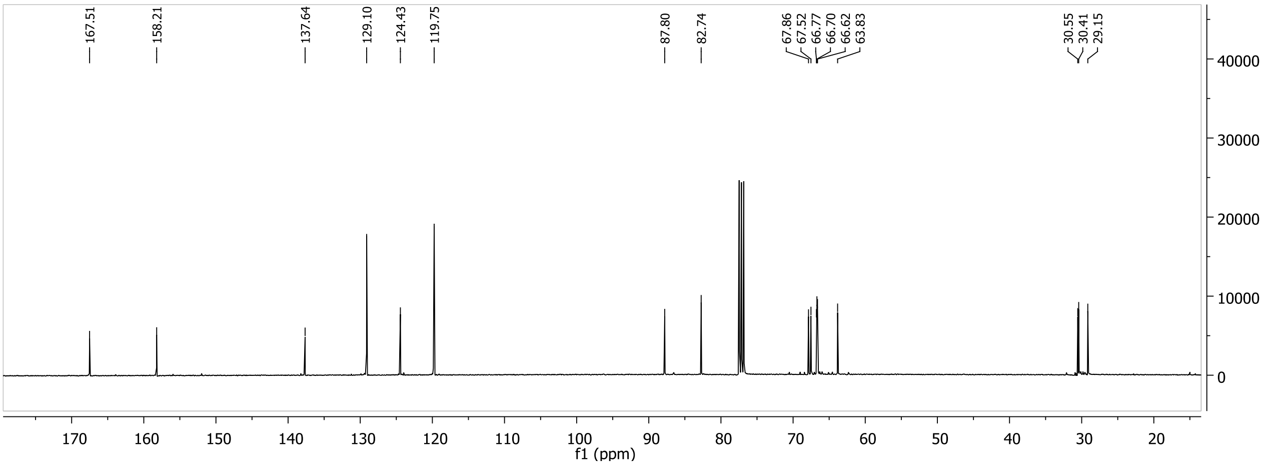

IR (neat): $\tilde{\nu}$ /cm^{-1} = 3358, 2928, 1680, 1643, 1598, 1520, 1441, 1294, 1234, 1159, 1114, 1071, 1025, 1003, 843, 757, 691, 584.

HR-ESI: m/z = 350.1960 $[M+H]^+$ (calculated for $C_{19}H_{27}NO_5$ m/z = 350.1962)

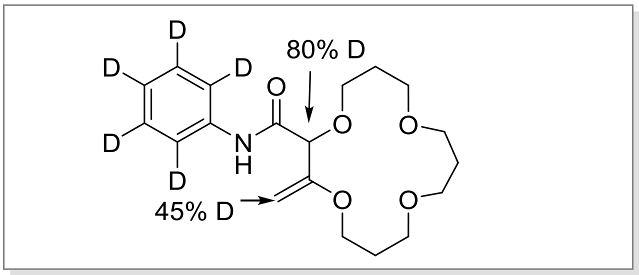

Deuteration Experiment:

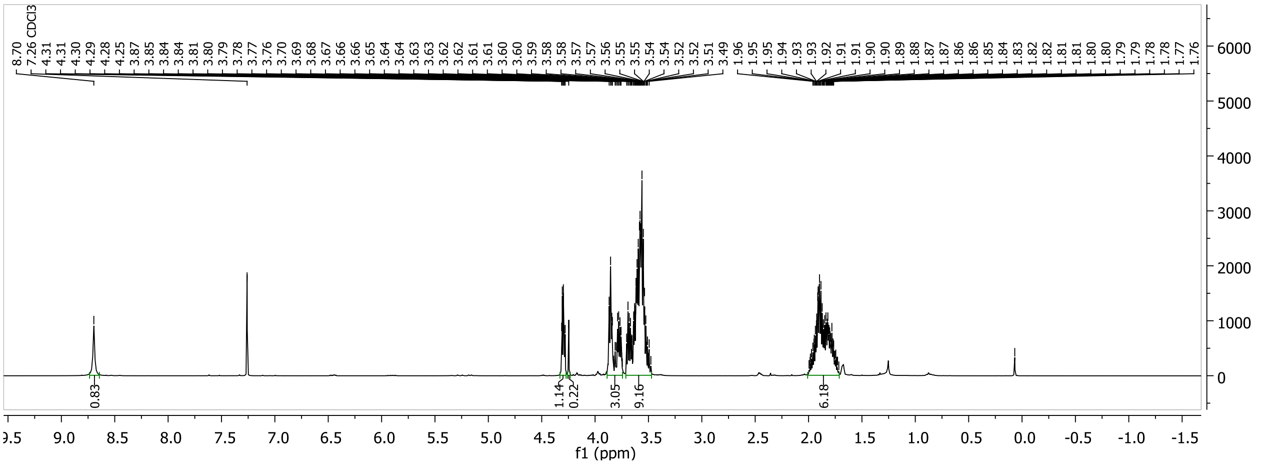

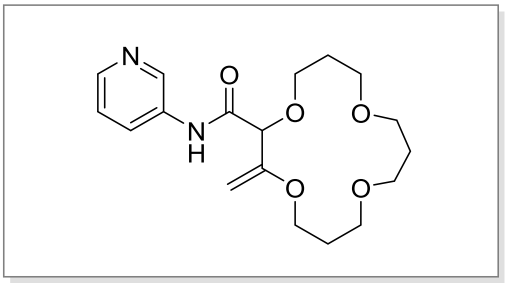

3k. Yield : 66% of viscous yellow liquid (40 mg)

R*f* : 0.57 (silica gel, mobile phase MeOH/ CH_2Cl_2 5/95), (using preparative silica TLC MeOH/Et_3N/ CH_2Cl_2 : 5/5/90)

^{1}H NMR (400 MHz, $CDCl_3$): δ/ppm = 1.73 – 2.04 (m, 6H), 3.50 – 3.70 (m, 9H), 3.79 – 3.89 (m, 3H), 4.27 (s, 1H), 4.31 (d, *J* = 2.8 Hz, 1H), 4.33 (d, *J* = 2.6 Hz, 1H), 7.27– 7.30 (m, 1H), 8.27 (dd, *J* = 2.6, 1.4 Hz, 1H), 8.29 (dd, *J* = 2.6, 1.4 Hz, 1H), 8.67 (s, 1H), 8.88 (s, 1H).

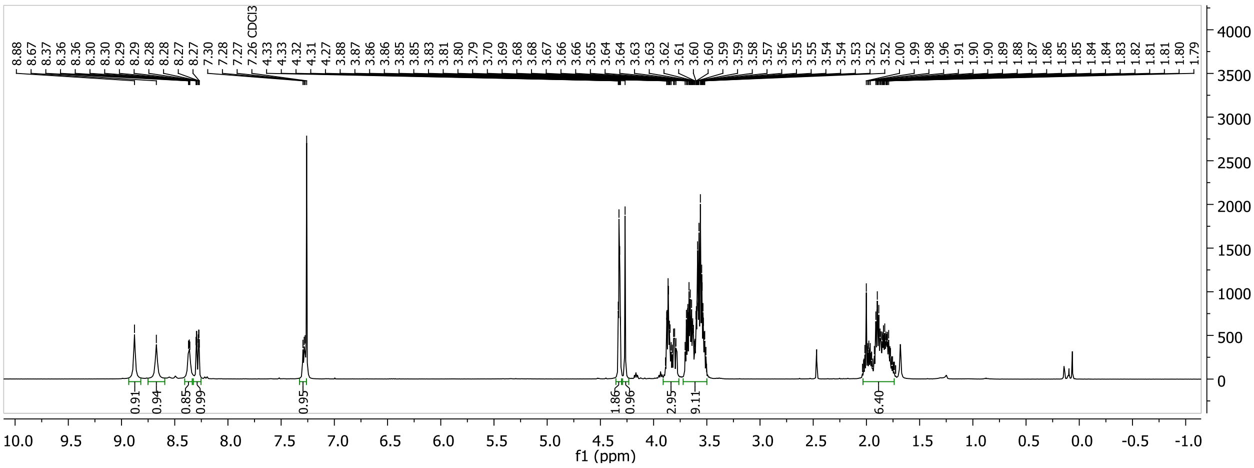

^{13}C NMR (101 MHz, $CDCl_3$): δ/ppm = 29.09 (1CH_2), 30.17 (1CH_2), 30.55 (1CH_2), 64.20 (1CH_2), 66.71 (1CH_2), 66.78 (1CH_2), 66.80 (1CH_2), 67.92 (1CH_2), 68.19 (1CH_2), 82.80 (1CH), 88.25 (1CH_2), 123.81 (1CH), 126.90 (1CH), 134.51 (1CH), 141.32 (1CH), 145.57 (1CH), 157.81 (1C), 168.38 (1C).

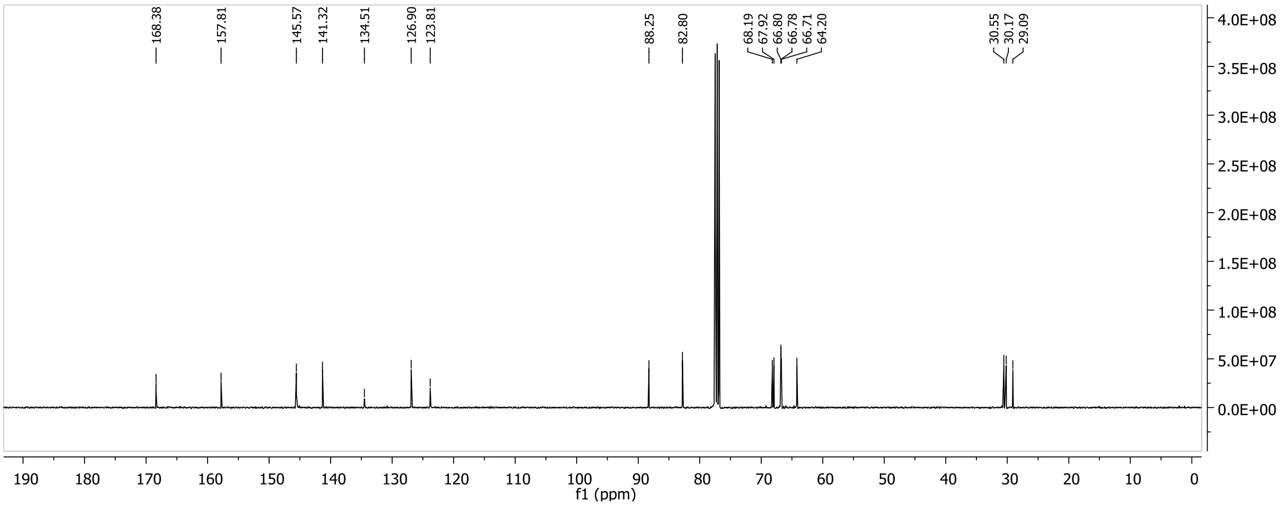

IR (neat): $\tilde{\nu}$ /cm^{-1} = 2926, 2868, 1688, 1587, 1523, 1482, 1422, 1330, 1289, 1094, 916, 804, 728,706, 644, 621, 531.

HR-ESI: m/z = 351.1916 $[M+H]^+$ (calculated for $C_{18}H_{27}N_2O_5$ m/z = 351.1915)

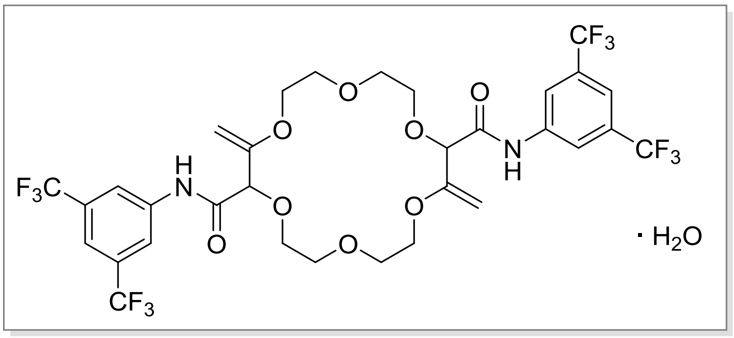

4a. Yield: 85 % of white solid (169 mg)

X-ray crystallized from CH_2Cl_2/ heptane (see crystallographic section)

R*f* : 0.52 (silica gel, mobile phase MeOH/ CH_2Cl_2 5/95)

^{1}H NMR (400 MHz, $CDCl_3$): δ/ppm = 3.56 – 3.59 (m, 2H), 3.63 – 3.72 (m, 6H), 3.76 – 3.94 (m, 6H, and H_2O-2H), 3.98 – 4.01 (m, 2H), 4.35 (d, *J* = 3.0 Hz, 2H), 4.40 (s, 2H), 4.50 (d, *J* = 3.0 Hz, 2H), 7.45 (s, 2H), 8.08 (s, 4H), 10.04 (s, 2H).

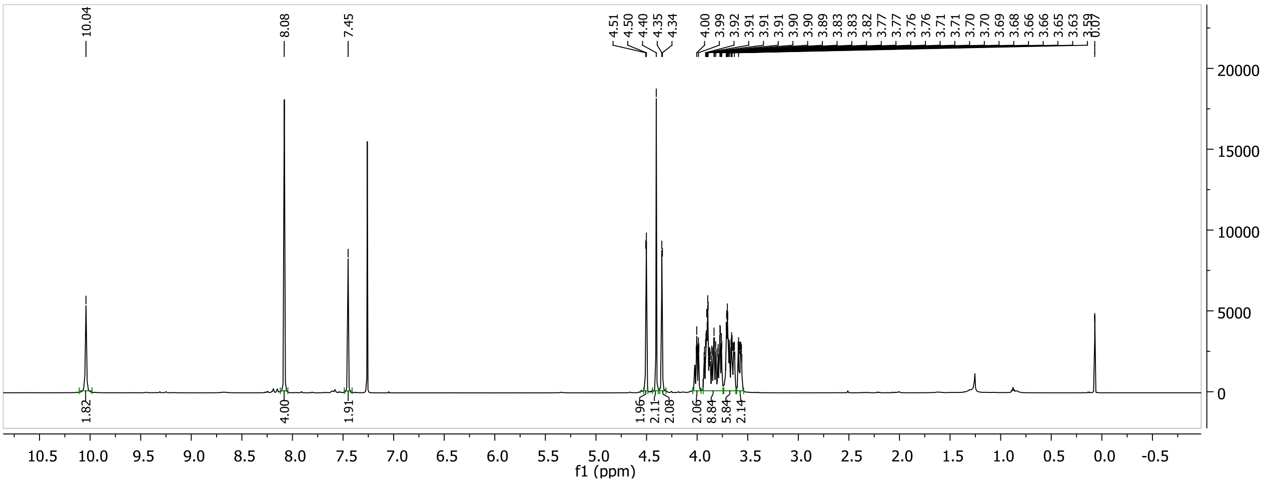

^{13}C NMR (101 MHz, $CDCl_3$): δ/ppm = 66.51 ($2CH_2$), 68.23 ($2CH_2$), 68.90 ($2CH_2$), 70.33 ($2CH_2$), 83.06 (2CH), 89.01 ($2CH_2$), 117.27 (2CH), 119.29 (4CH), 123.18 (q, *J* = 272.6 Hz, $4CF_3$), 132.13 (q, *J* = 33.1 Hz, 4C), 139.64 (2C), 155.85 (2C), 168.08 (2C).

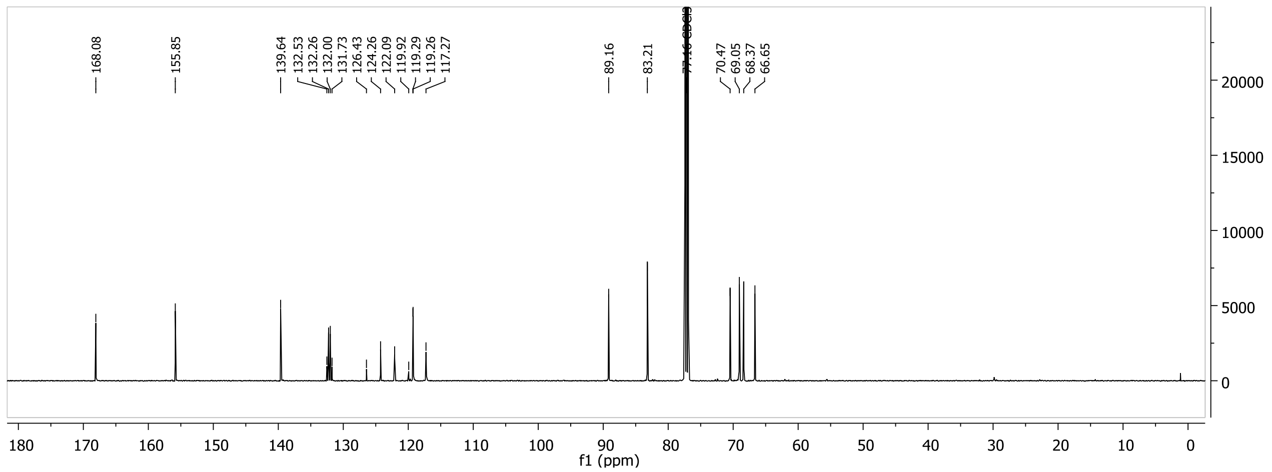

^{19}F NMR (282 MHz, $CDCl_3$): δ/ppm = -62.35 (s, 12F).

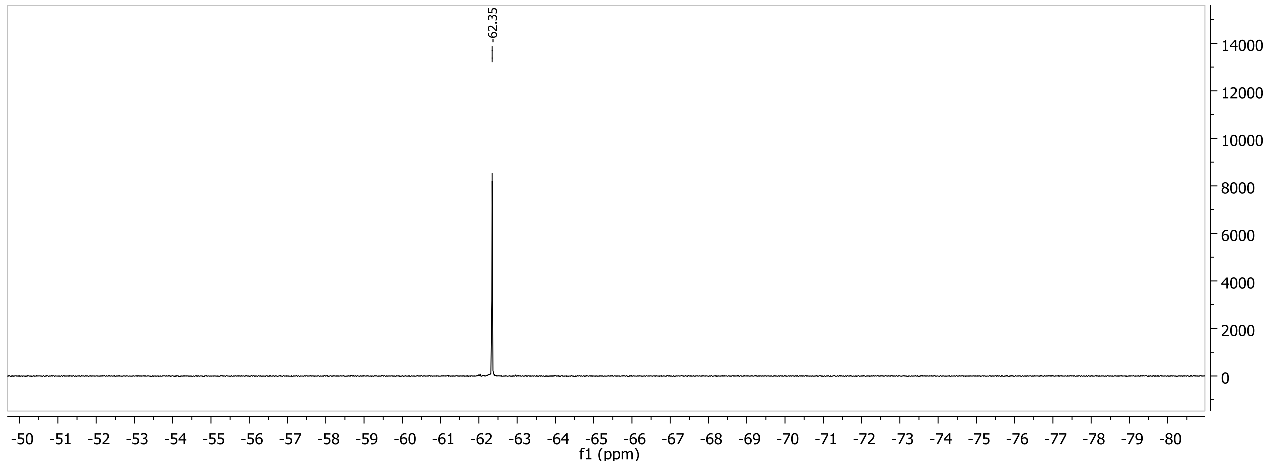

IR (neat): $\tilde{\nu}$ /cm^{-1} = 3466, 3234, 3110, 2936, 1694, 1627, 1575, 1472, 1443, 1381, 1275, 1218, 1176, 1123, 1073, 1004, 938, 886, 831, 734, 703, 680, 588.

HR-ESI: m/z = 799.1866 [M+H]$^+$ (calculated for $C_{32}H_{31}F_{12}N_2O_8$ m/z = 799.1883)

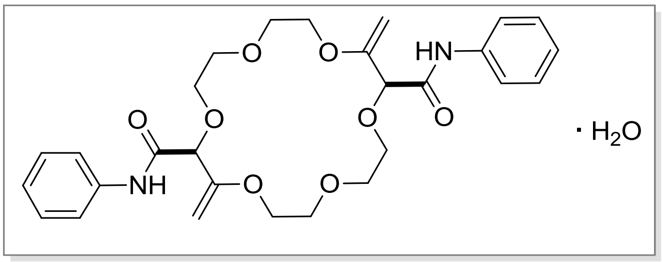

4b. Yield: 53% of white solid (56 mg)

R*f*: 0.2 (silica gel, mobile phase CH_2Cl_2/EtOAc/MeOH/Et_3N 5/5/1/0.1)

^{1}H NMR (400 MHz, $CDCl_3$): δ/ppm = 3.24 bs (H_2O), 3.54 – 3.71 (m, 8H), 3.74 – 3.81 (m, 6H), 3.87 – 3.93 (m, 2H), 4.22 (d, *J* = 2.8 Hz, 2H), 4.32 (s, 2H), 4.38 (d, *J* = 2.7 Hz, 2H), 6.95 – 7.02 (m, 2H), 7.13 – 7.18 (m, 4H), 7.53 – 7.58 (m, 4H), 9.08 (s, 2H).

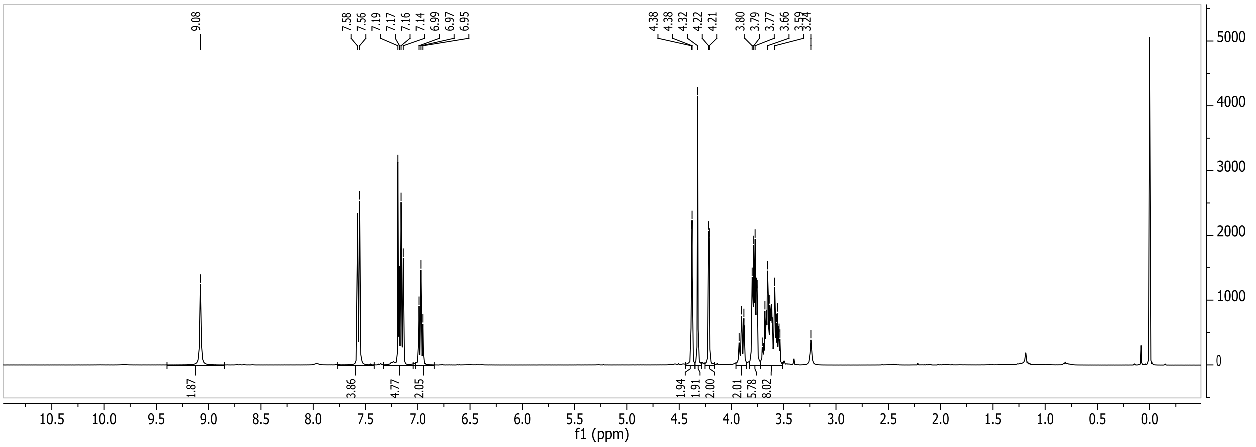

^{13}C NMR (101 MHz, $CDCl_3$)): δ/ppm = 67.1 (2CH_2), 68.7 (2CH_2), 69.1 (2CH_2), 70.3 (2CH_2), 82.9 (2CH), 88.0 (2CH_2), 119.9 (4CH), 124.3 (2CH), 128.9 (4CH), 138.0 (2C), 157.0 (2C), 167.5 (2C).

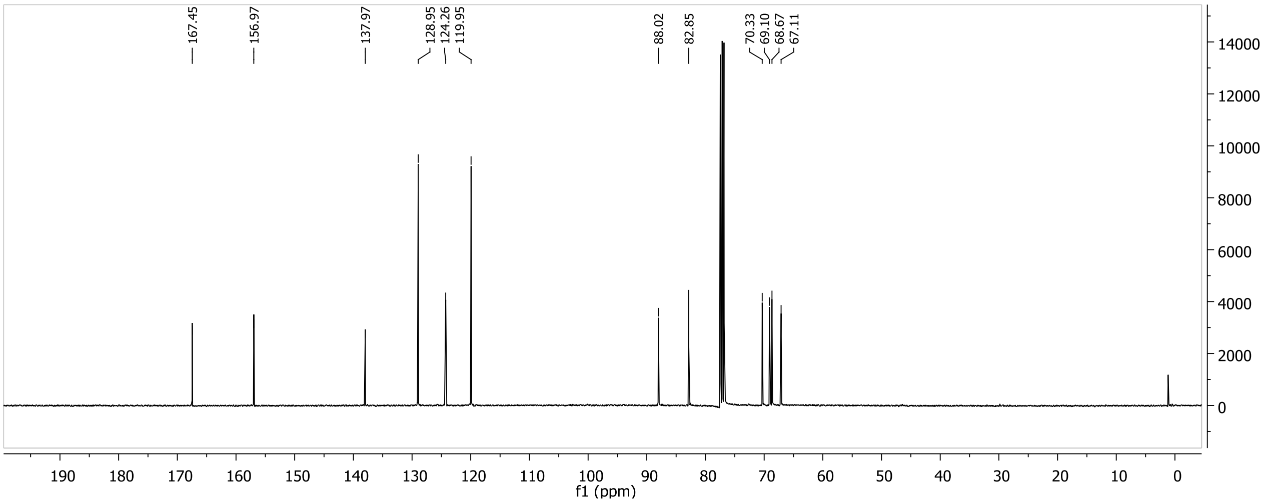

IR (neat): $\tilde{\nu}$ /cm^{-1} = 3509, 3463, 3254, 3193, 3130, 3072, 2918, 2878, 1687, 1595, 1547, 1494, 1467, 1442, 1360, 1325, 1302, 1285, 1264, 1244, 1103, 1075, 1025, 999, 942, 931, 902, 842, 821, 796, 779, 756, 696.

HR-ESI: m/z = 527.2391 $[M+H]^+$ (calculated for $C_{28}H_{35}N_2O_8$ m/z = 527.2393)

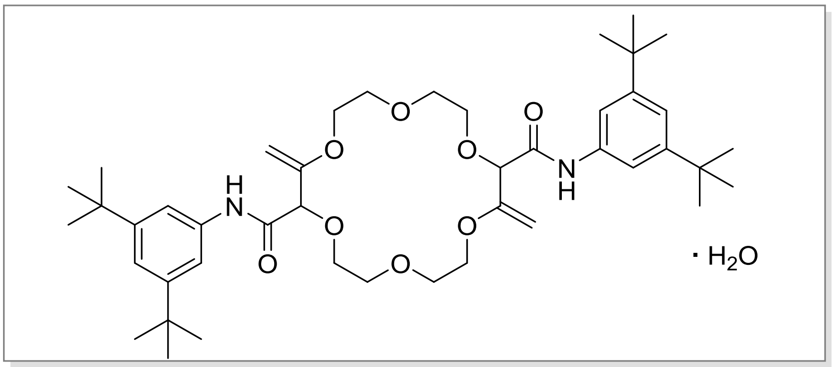

4c. Yield: 40 % of white solid (75 mg)

R*f* : 0.5 (silica gel, mobile phase MeOH/ CH_2Cl_2 5/95)

^{1}H NMR (400 MHz, $CDCl_3$): δ/ppm = 1.16 (s, 36H), 2.92 (bs, H_2O), 3.49 – 3.61 (m, 6H), 3.71 – 3.92 (m, 10H), 4.19 (d, *J* = 2.7 Hz, 2H), 4.33 (s, 2H), 4.37 (d, *J* = 2.7 Hz, 2H), 6.99 (t, *J* = 1.8 Hz, 2H), 7.42 (d, *J* = 1.7 Hz, 4H), 9.66 (s, 2H)

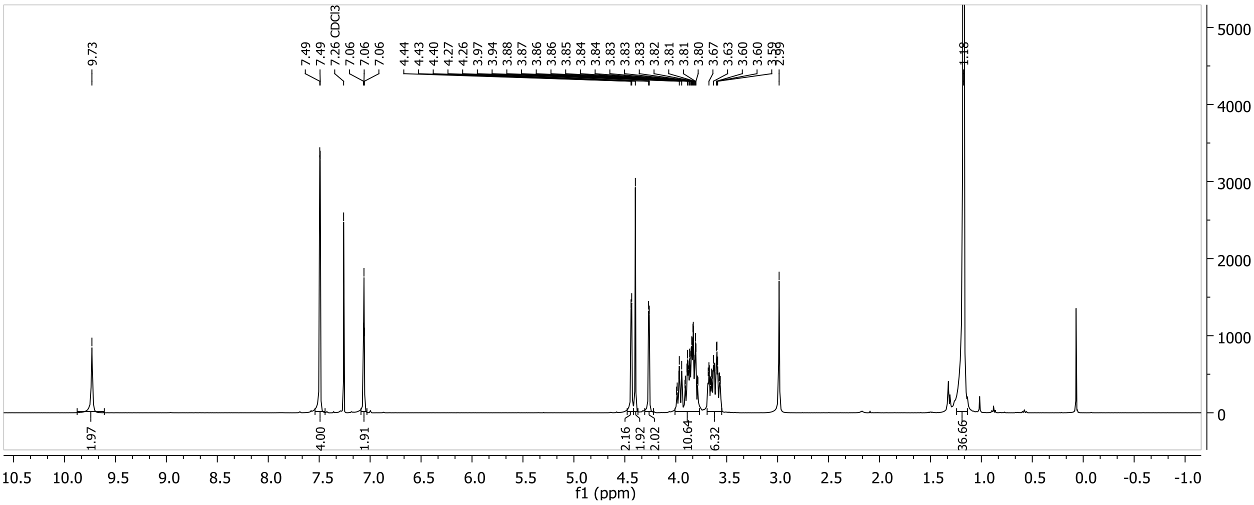

^{13}C NMR (101 MHz, $CDCl_3$): δ/ppm = 31.42 (12CH_3), 34.90 (4C), 67.06 (2CH_2), 68.12 (2CH_2), 69.37 (2CH_2), 71.04 (2CH_2), 83.63 (2CH), 87.91 (2CH_2), 114.63 (4CH), 118.37 (2CH), 137.72 (2C), 151.31 (4C), 156.77 (2C), 167.34 (2C).

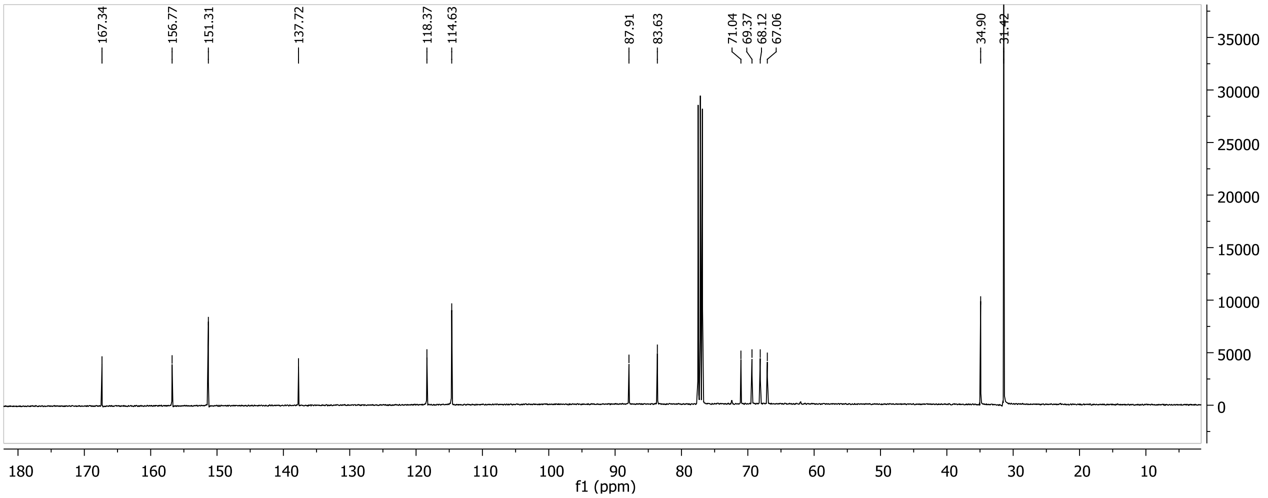

IR (neat): $\tilde{\nu}$ /cm^{-1} = 3506, 3264, 2957, 2869, 1686, 1603, 1560, 1443, 1393, 1361, 1292, 1246, 1102, 1073, 997, 929, 896, 869, 824, 799, 728, 708, 571.

HR-ESI: m/z = 751.4890 $[M+H]^+$ (calculated for $C_{42}H_{67}N_2O_8$ m/z = 751.4892)

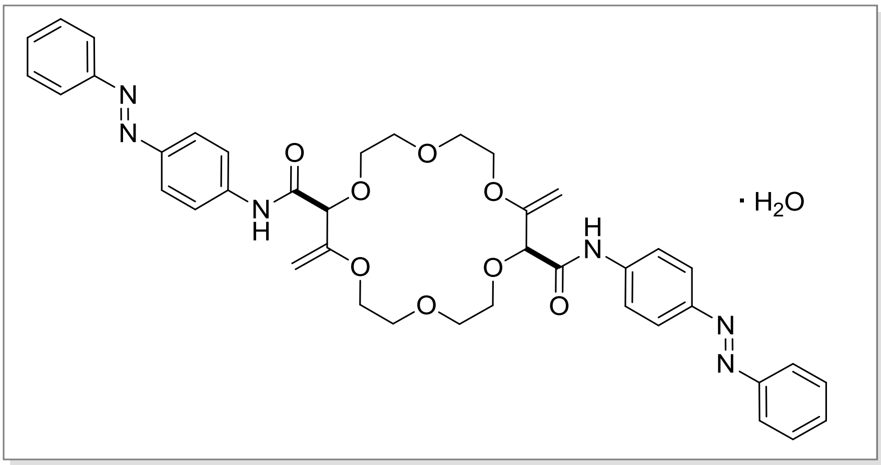

4d. Yield: 35 % of orange solid (61 mg)

R*f* : 0.3 (silica gel, mobile phase CH_2Cl_2/EtOAc/MeOH/Et_3N 5/5/1/0.1)

^{1}H NMR (400 MHz, $CDCl_3$): δ/ppm = 2.47 (bs, H_2O), 3.61 – 3.66 (m, 4H), 3.70 – 3.78 (m, 4H), 3.82 – 4.04 (m, 8H), 4.33 (d, *J* = 2.7 Hz, 2H), 4.40 (s, 2H), 4.48 (d, *J* = 2.6 Hz, 2H), 7.39 – 7.42 (m, 6H), 7.79 – 7.89 (m, 12H), 9.78 (s, 2H).

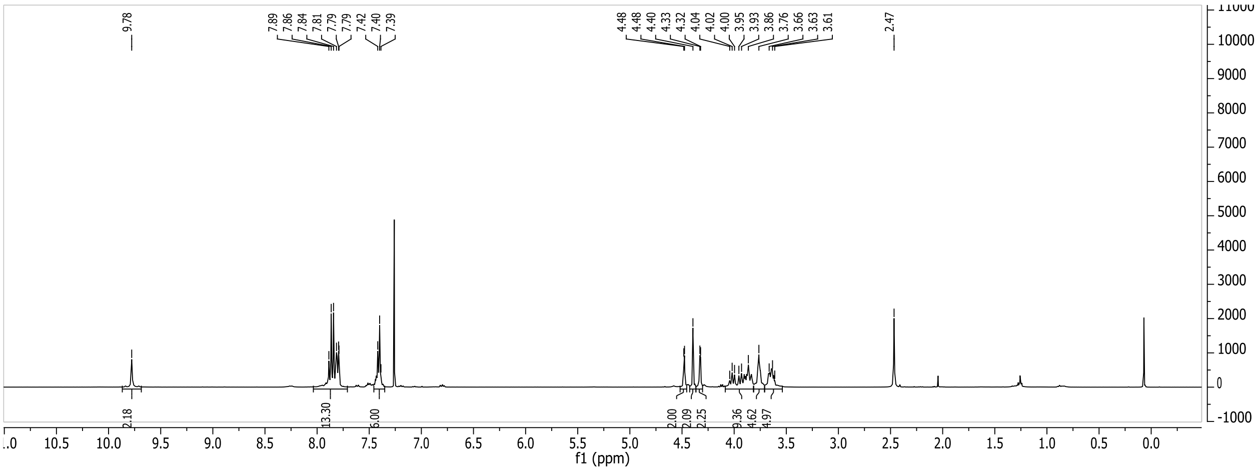

^{13}C NMR (101 MHz, $CDCl_3$): δ/ppm = 66.8 ($2CH_2$), 68.6 ($2CH_2$), 69.8 ($2CH_2$), 70.7 ($2CH_2$), 84.4 (2CH), 89.2 ($2CH_2$), 120.6 (4CH),123.2 (4CH), 124.3 (4CH), 129.4 (4CH), 131.0 (2CH), 141.3 (2C), 149.4 (2C), 153.1 (2C), 156.4 (2C), 168.1 (2C).

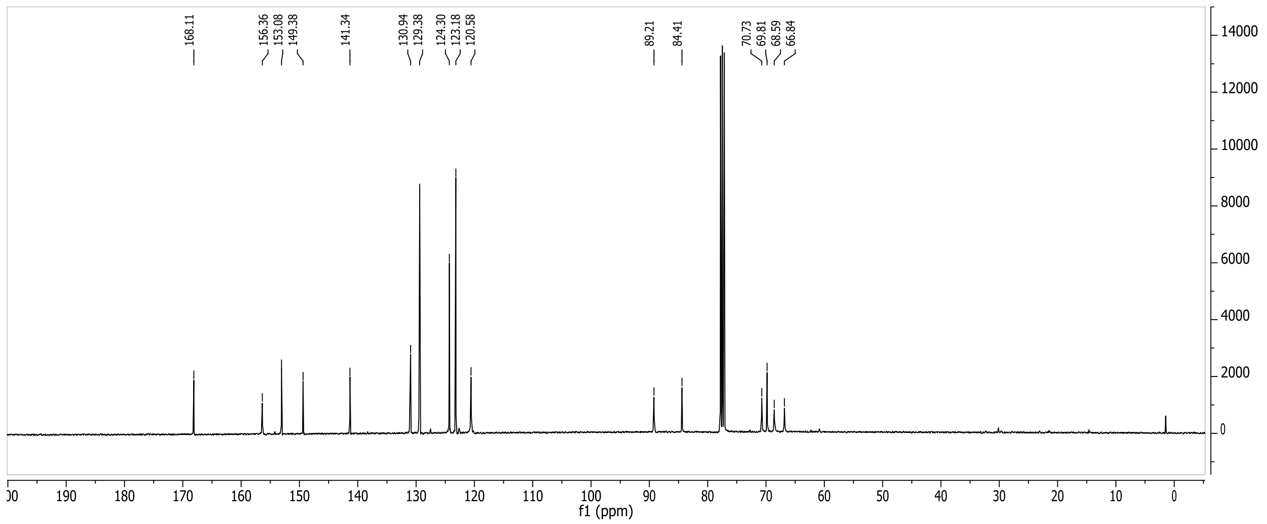

IR (neat): $\tilde{\nu}$ /cm^{-1} = 3285, 3058, 2921, 2879, 1689, 1638, 1595, 1527, 1460, 1438, 1407, 1293, 1245, 1139, 1094, 1073, 993, 927, 834, 766, 722, 687.

HR-ESI: m/z = 735.3118 $[M+H]^+$ (calculated for $C_{40}H_{43}N_6O_8$ m/z = 735.3142)

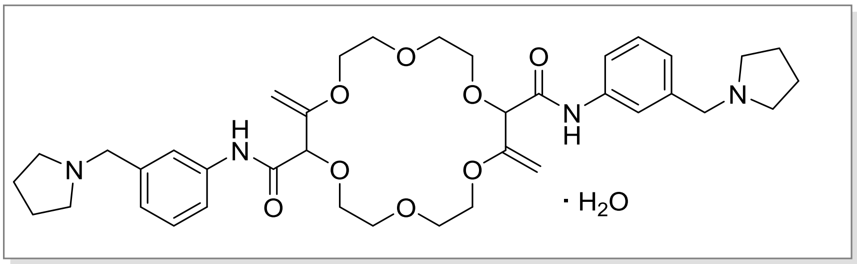

4e. Yield: 30 % of yellow solid (52 mg)

R*f* : 0.12 (silica gel, mobile phase MeOH/ CH_2Cl_2 10/90), (using preparative silica TLC MeOH/Et_3N/ CH_2Cl_2 : 5/5/90)

^{1}H NMR (400 MHz, C_6D_6): δ/ppm = 1.57 – 1.60 (m, 8H), 2.42 (bs, H_2O), 2.32 – 2.41 (m, 8H), 2.72 (ddd, *J* = 11.3, 3.6, 1.6 Hz, 2H), 2.96 – 3.06 (m, 6H), 3.20 – 3.36 (m, 6H), 3.40 – 3.49 (m, 6H), 3.94 (d, *J* = 2.3 Hz, 2H), 4.31 (d, *J* = 2.3 Hz, 2H), 4.33 (s, 2H), 7.13 – 7.23 (m, 4H), 8.22 (d, *J* = 8.1, 2H), 8.28 (s, 2H), 10.10 (s, 2H).

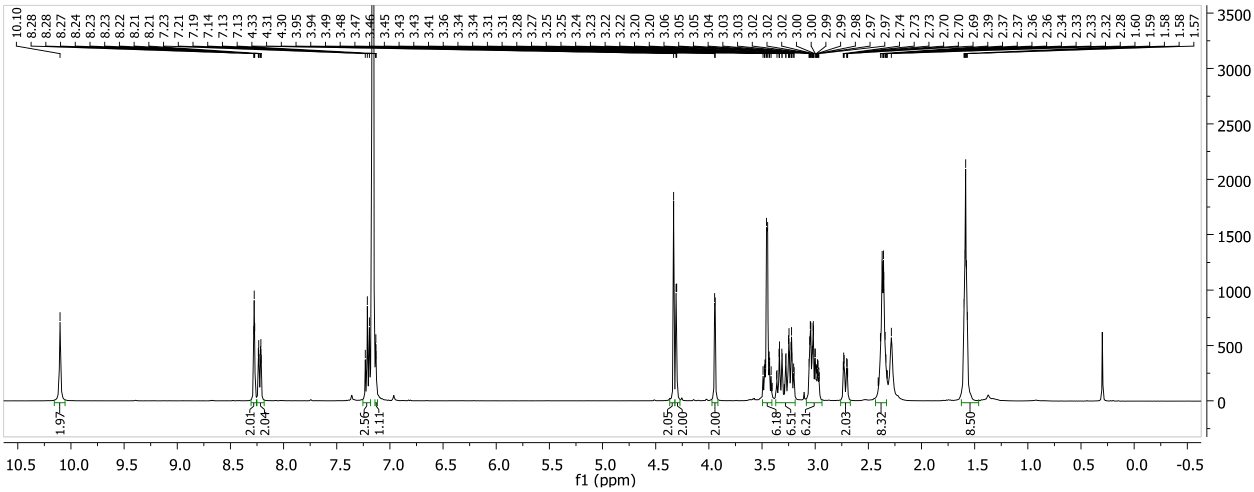

^{13}C NMR (101 MHz, C_6D_6): δ/ppm = 23.99 ($4CH_2$), 54.21 ($4CH_2$), 60.68 ($2CH_2$), 66.55 ($2CH_2$), 68.12 ($2CH_2$), 69.19 ($2CH_2$), 70.39 ($2CH_2$), 84.49 (2CH), 87.53 ($2CH_2$), 118.66 (2CH), 120.37 (2CH), 124.31 (2CH), 129.06 (2CH), 139.70 (2C), 141.39 (2C), 157.36 (2C), 167.03 (2C).

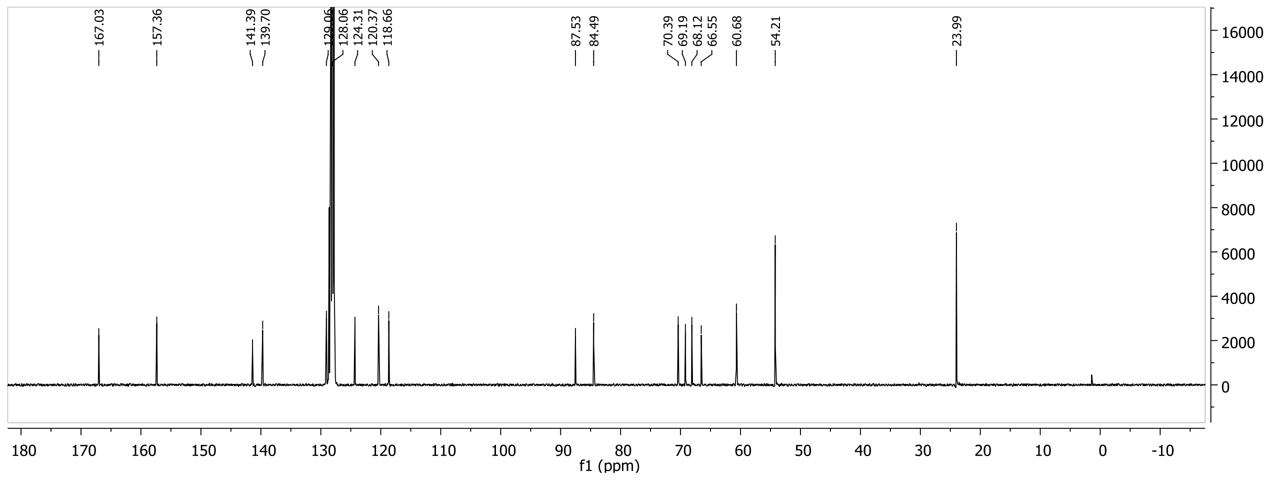

IR (neat): $\tilde{\nu}$ /cm^{-1} = 3500, 3268, 2920, 2783, 2787, 1680, 1612, 1593, 1549, 1487, 1443, 1376, 1286, 1243, 1126, 1085, 998, 938, 899, 828, 786, 696, 537.

HR-ESI: m/z = 693.3860 [M+H]$^+$ (calculated for $C_{38}H_{53}N_4O_8$ m/z = 693.3856)

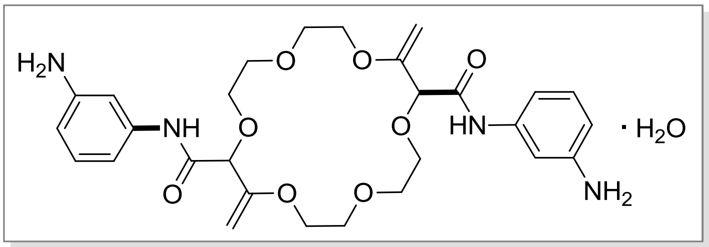

4f. Yield: 18 % of white solid (50 mg)

R*f* : 0.11 (silica gel, mobile phase CH_2Cl_2/EtOAc/MeOH/Et_3N 5/5/1/0.1)

^{1}H NMR (400 MHz, $CDCl_3$): δ/ppm = 1.9 (bs, H_2O), 3.57 – 3.59 (m, 6H), 3.63 - 3.85 (m, 10 H, +2 NH_2), 4.27 (d, *J* = 2.6 Hz, 2H), 4.34 (s, 2H), 4.43 (d, *J* = 2.6 Hz, 2H), 6.36 – 6.39 (m, 2H), 7.01 – 7.05 (m, 2H), 7.12 – 7.14 (m, 4H), 9.64 (s, 2H).

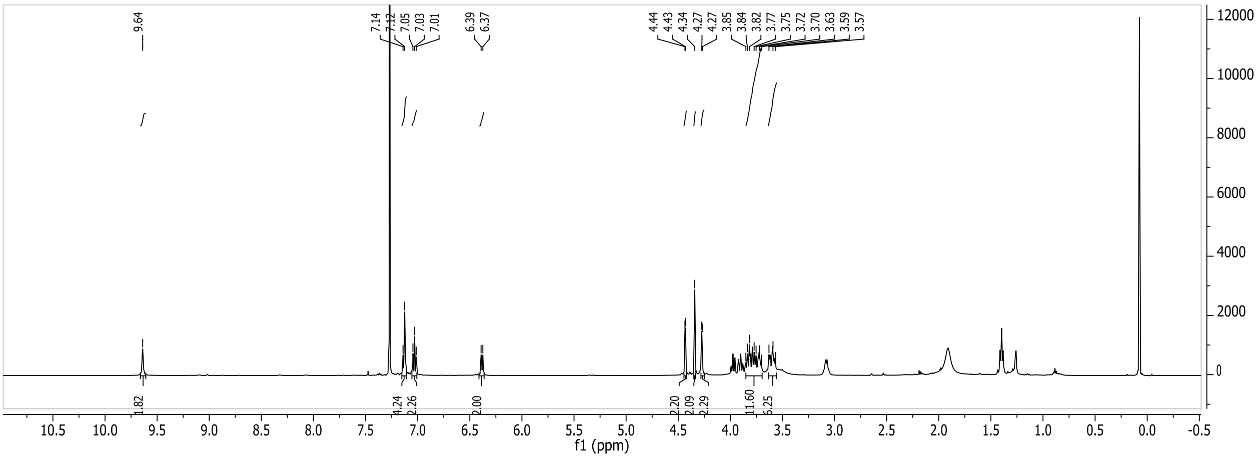

^{13}C NMR (101 MHz, $CDCl_3$): δ/ppm = 65.5 ($2CH_2$), 67.1 ($2CH_2$), 68.2 ($2CH_2$), 69.3 ($2CH_2$), 82.6 (2CH), 87.2 ($2CH_2$), 105.6 (2CH), 109.2 (2CH), 109.9 (2CH), 128.5 (2C), 138.1 (2CH), 145.9 (2C), 155.3 (2C), 166.3 (2C).

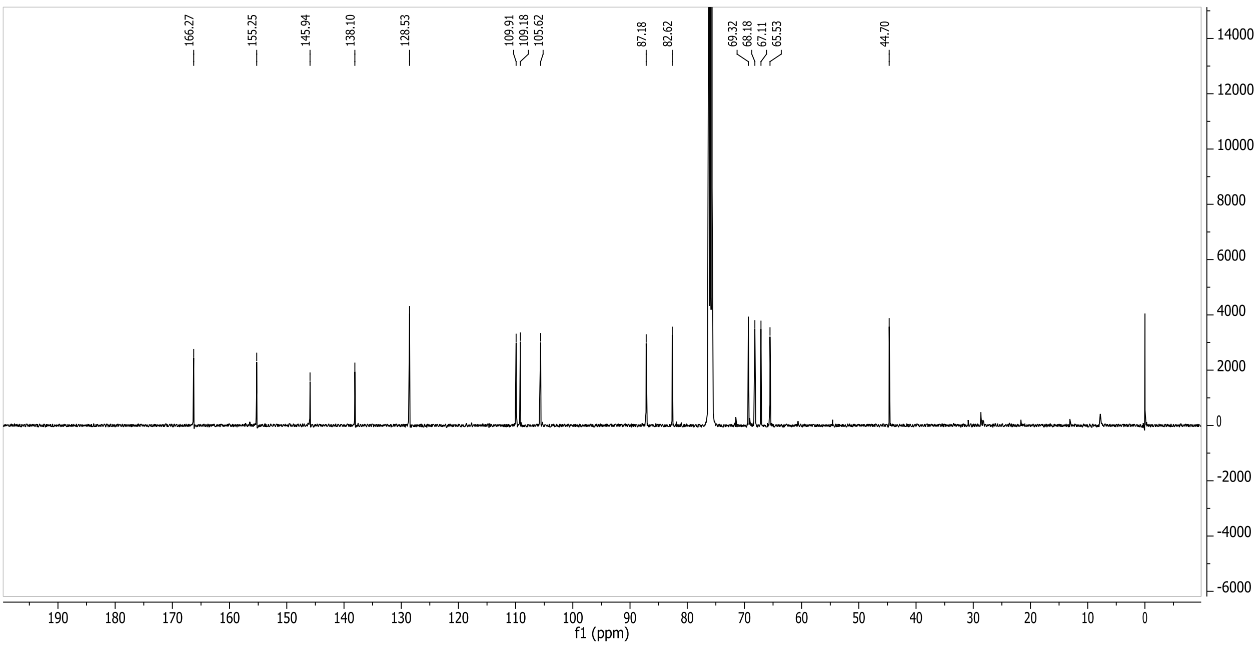

IR (neat): $\tilde{\nu}$ /cm^{-1} = 3330, 2925, 2245, 1675, 1608, 1544, 1495, 1457, 1372, 1288, 1244, 1163, 1094, 1075, 993, 908, 829, 778, 725, 689.

HR-ESI: m/z = 557.2612 [M+H]$^+$ (calculated for $C_{28}H_{37}N_4O_8$ m/z = 557.2611)

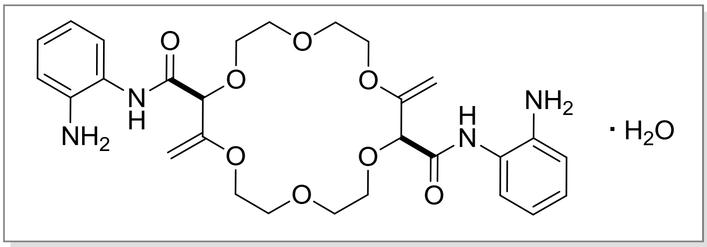

4g. Yield: 36 % of white solid (101 mg)

$\mathbf{R}_{\boldsymbol{f}}$: 0.14 (silica gel, mobile phase CH_2Cl_2/EtOAc/MeOH/Et_3N 5/5/1/0.1)

^{1}H NMR (500 MHz, $CDCl_3$): δ/ppm = 2.48 (bs, H_2O), 3.54 – 3.66 (m, 2H), 3.67 – 3.68 (m, 4H), 3.81 – 3.94 (m, 10 H, + 2 NH_2), 4.30 (d, *J* = 2.6 Hz, 2H), 4.37 (s, 2H), 4.41 (d, *J* = 2.6 Hz, 2H), 6.53 (d, *J* = 7.9 Hz, 2H), 6.64 – 6.67 (m, 2H), 6.89 – 6.92 (m, 2H), 7.30 (d, *J* = 7.2 Hz, 2H), 9.26 (s, 2H).

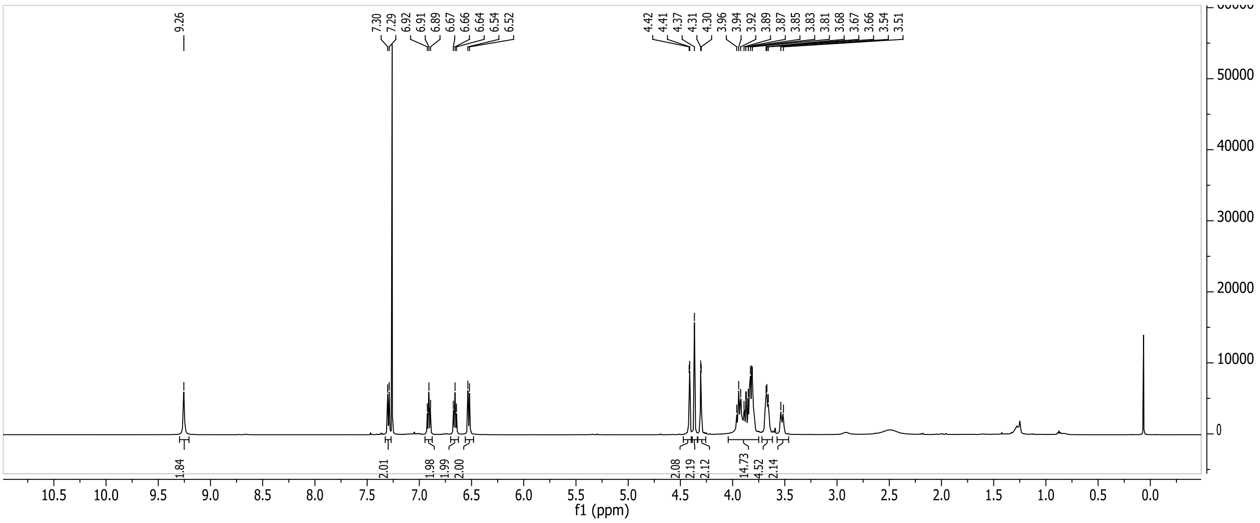

^{13}C NMR (101 MHz, $CDCl_3$): δ/ppm = 67.0 (2CH_2), 67.5 (2CH_2), 68.9 (2CH_2), 70.4 (2CH_2), 83.1 (2CH), 89.3 (2CH_2), 116.8 (2CH), 118.7 (2CH), 123.5 (2C), 125.7 (2CH), 126.8 (2CH), 140.9 (2C), 156.1 (2C), 167.5 (2C).

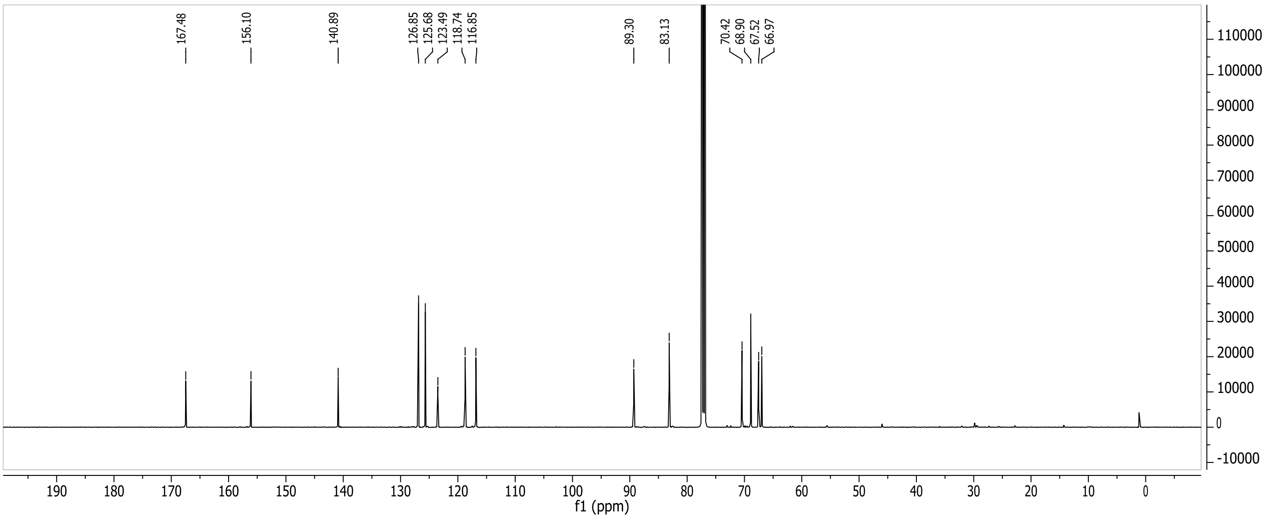

IR (neat): $\tilde{\nu}$ /cm^{-1} = 3335, 3231, 2924, 2613, 2498, 1668, 1633, 1533, 1503, 1454, 1398, 1359, 1283, 1243, 1098, 1073, 998, 930, 905, 817, 749, 697.

HR-ESI: m/z = 557.2612 [M+H]$^+$ (calculated for $C_{28}H_{37}N_4O_8$ m/z = 557.2611)

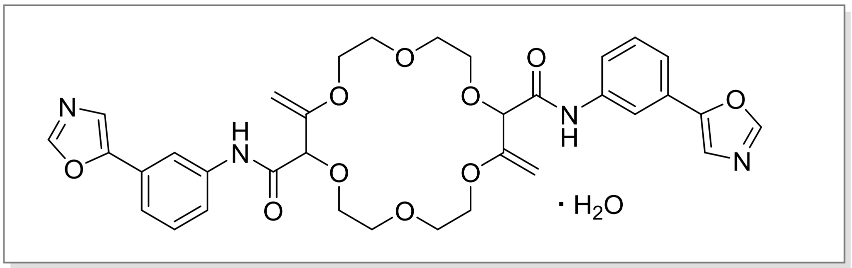

4h. Yield: 46 % of yellow solid (75 mg)

R*f* : 0.28 (silica gel, mobile phase MeOH/ CH_2Cl_2 5/95), (using preparative silica TLC MeOH/CH_2Cl_2 : 5/90)

^{1}H NMR (400 MHz, C_6D_6): δ/ppm = 2.40 (bs, H_2O), 3.60 – 3.69 (m, 4H), 3.74 – 3.79 (m, 4H), 3.85 – 3.94 (m, 6H), 4.00 – 4.05 (m, 2H), 4.32 (d, *J* = 2.8 Hz, 2H), 4.39 (s, 2H), 4.48 (d, *J* = 2.8 Hz, 2H), 7.15 – 7.23 (m, 6H), 7.58 (d, *J* = 7.9 Hz, 2H), 7.75 (s, 2H), 7.98 (s, 2H), 9.71 (s, 2H).

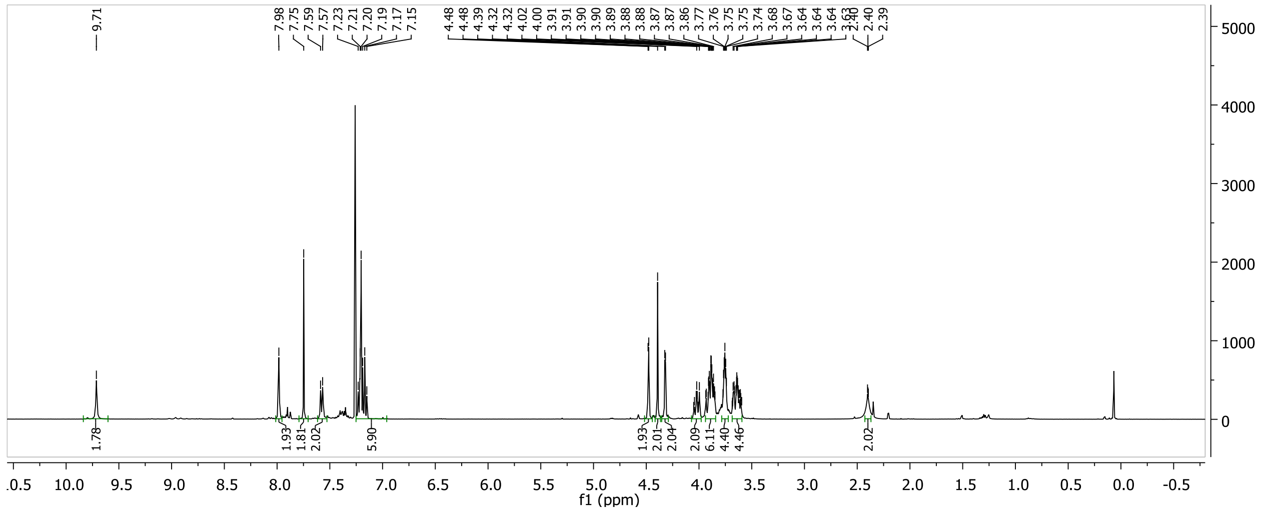

^{13}C NMR (101 MHz, C_6D_6): δ/ppm = 66.69 (2CH_2), 68.51 (2CH_2), 69.19 (2CH_2), 70.28 (2CH_2), 83.46 (2CH), 88.70 (2CH_2), 115.53 (2CH), 119.88 (2CH), 119.92 (2CH), 121.77 (2CH), 128.29 (2C), 129.43 (2CH), 138.83 (2C), 150.43 (2C), 151.28 (2C), 156.41 (2C), 167.71 (2C).

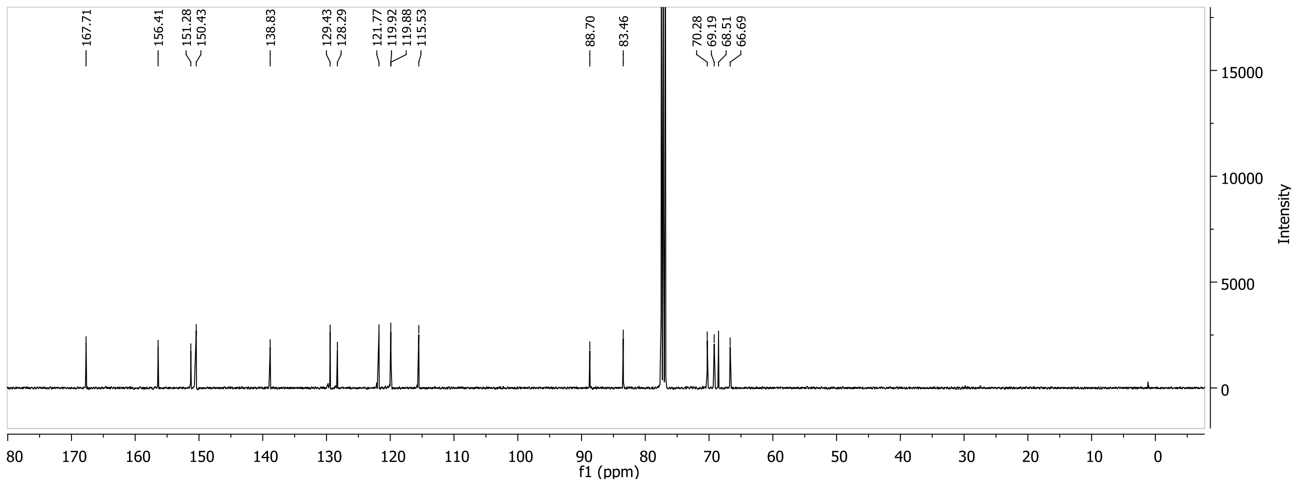

IR (neat): $\tilde{\nu}$ /cm^{-1} = 3321, 3119, 2924, 1683, 1617, 1580, 1596, 1535, 1496, 1290, 1241, 1085, 991, 951, 886, 823, 789, 689, 587.

HR-ESI: m/z = 661.2499 $[M+H]^+$ (calculated for $C_{34}H_{37}N_4O_{10}$ m/z = 661.2504)

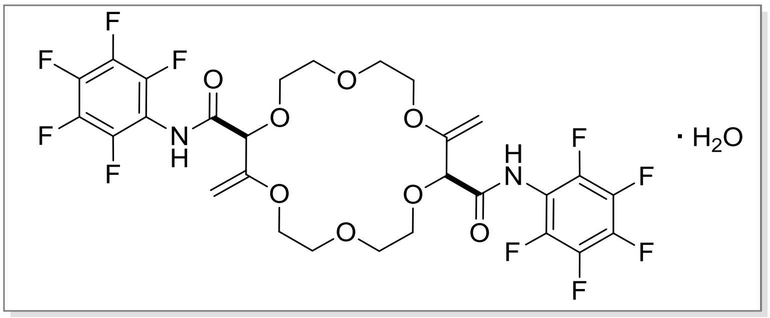

4i. Yield: 20 % of pale yellow solid (29 mg)

R*f* : 0.5 (silica gel, mobile phase CH_2Cl_2/EtOAc/MeOH/Et_3N 5/5/1/0.1)

^{1}H NMR (500 MHz, $CDCl_3$): δ/ppm = 2.78 (bs, H_2O), 3.50 – 3.54 (m, 2H), 3.55 – 3.60 (m, 2H), 3.65 – 3.69 (m, 2H), 3.78 – 3.89 (m, 6H), 3.93 – 3.99 (m, 4H), 4.28 (d, *J* = 12.8 Hz, 2H), 4.41 – 4.42 (m, 4H), 10.28 (s, 2H).

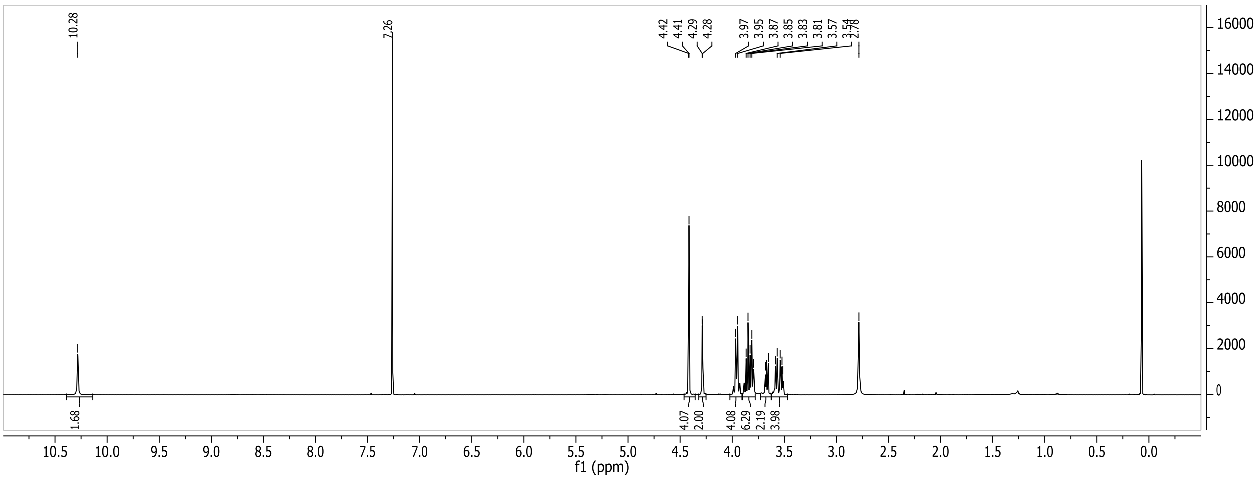

^{13}C NMR (126 MHz, $CDCl_3$): δ/ppm = 66.8 ($2CH_2$), 67.6 ($2CH_2$), 69.5 ($2CH_2$), 71.0 ($2CH_2$), 83.5 (2CH), 89.0 ($2CH_2$), 112.5 (m, 2C), 137.8 (m, 4CF), 139.8(m, 2CF), 143.0 (m, 4CF), 155.5 (2C), 168.3 (2C).

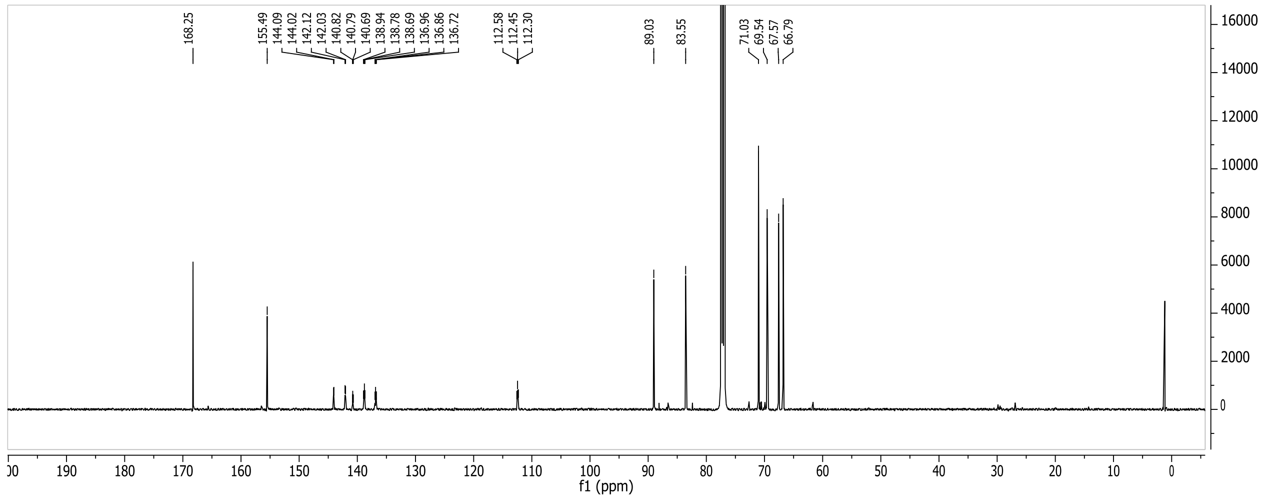

^{19}F NMR (282 MHz, $CDCl_3$): δ/ppm = -163.43 – -163.25 (m, 4F), -158.08 – -157.93 (m, 2F), -145.15 – -145.06 (m, 4F).

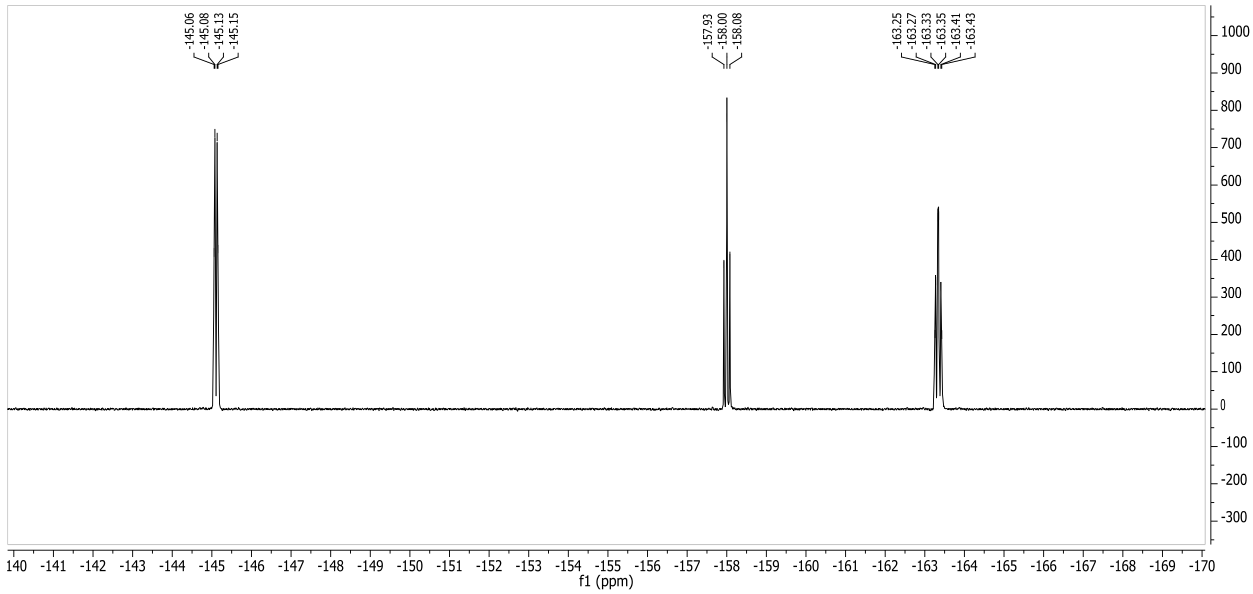

IR (neat): $\tilde{\nu}$ /cm^{-1} = 3484, 2931, 1702, 1642, 1522, 1493, 1456, 1295, 1277, 1246, 1108, 1047, 1000, 956, 927, 835, 694.

HR-ESI: m/z = 707.1435 $[M+H]^+$ (calculated for $C_{28}H_{25}F_{10}N_2O_8$ m/z = 707.1446)

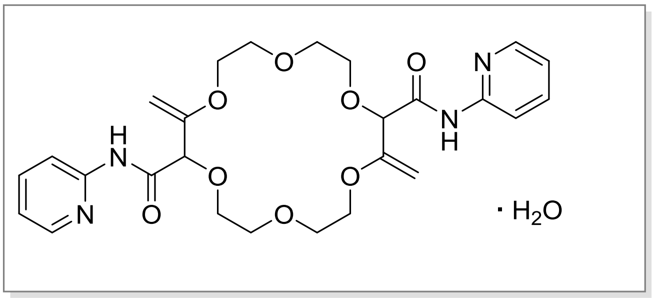

4j. Yield: 52 % of yellow solid (68 mg)

R*f* : 0.3 (silica gel, mobile phase MeOH/ CH_2Cl_2 5/95), (using preparative silica TLC MeOH/Et_3N/ CH_2Cl_2 : 5/5/90)

^{1}H NMR (400 MHz, $CDCl_3$): δ/ppm = 3.64 – 3.69 (m, 2H), 3.71 – 3.86 (m, 10H, and H_2O-2H), 3.89 – 4.02 (m, 4H), 4.29 (d, *J* = 2.8 Hz, 2H), 4.39 (d, *J* = 2.8 Hz, 2H), 4.48 (s, 2H), 7.01 (dd, *J* = 7.3, 5.0 Hz, 2H), 7.67 (td, *J* = 8.0, 2.0 Hz, 2H), 8.23 (d, *J* = 8.4 Hz, 2H), 8.28 (dd, *J* = 5.1, 1.8 Hz, 2H), 9.22 (s, 2H).

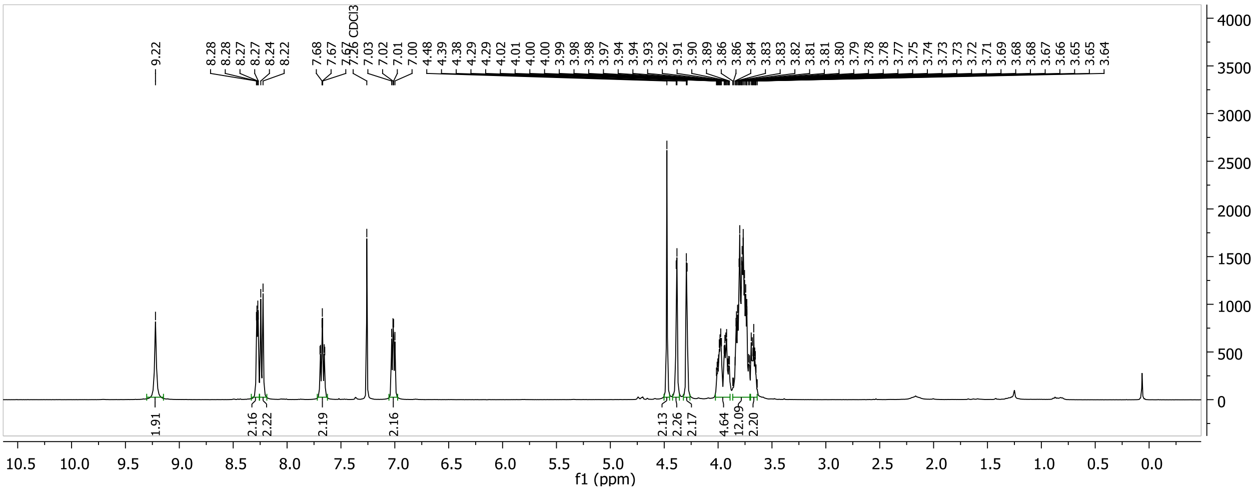

^{13}C NMR (101 MHz, $CDCl_3$): δ/ppm = 68.09 ($2CH_2$), 68.61 ($2CH_2$), 69.22 ($2CH_2$), 70.57 ($2CH_2$), 81.82 (2CH), 87.37 ($2CH_2$), 114.26 (2CH), 120.03 (2CH), 138.25 (2CH), 148.13 (2CH), 151.07 (2C), 157.36 (2C), 168.15 (2C).

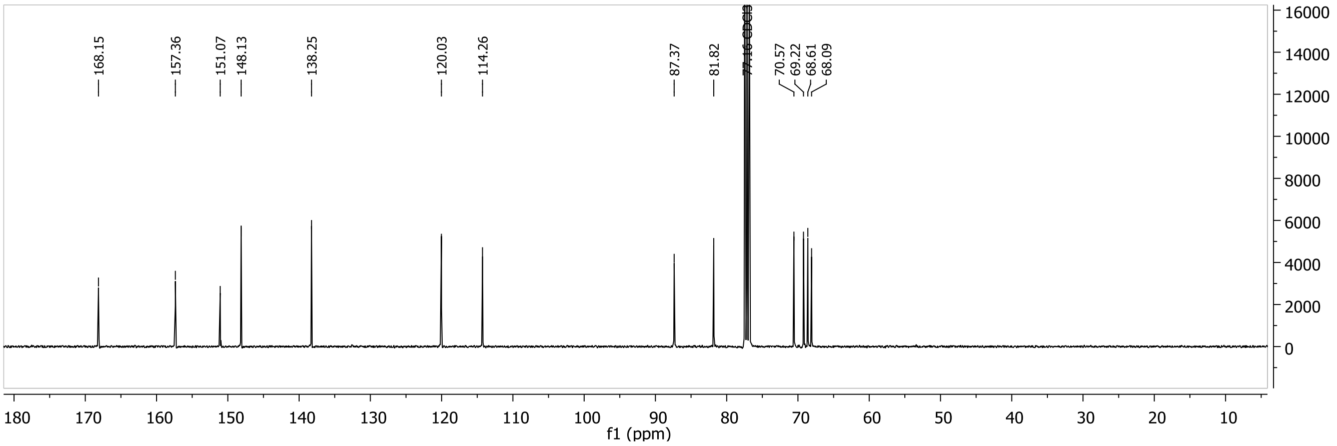

IR (neat): $\tilde{\nu}$ /cm^{-1} = 3383, 2926, 2875, 1759, 1694, 1636, 1575, 1513, 1459, 1432, 1298, 1244, 1137, 1082, 990, 927, 821, 777, 750, 665, 582.

HR-ESI: m/z = 529.2317 $[M+H]^+$ (calculated for $C_{26}H_{33}N_4O_8$ m/z = 529.2293)

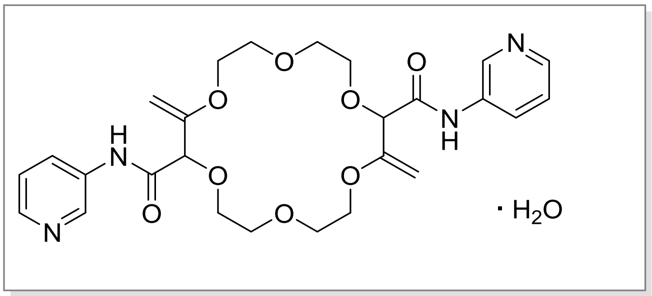

4k. Yield: 60 % of yellow solid (79 mg)

R*f* : 0.26 (silica gel, mobile phase MeOH/ CH_2Cl_2 5/95), (using preparative silica TLC MeOH/Et_3N/ CH_2Cl_2 : 10/10/80)

^{1}H NMR (400 MHz, $CDCl_3$): δ/ppm = 3.59 – 3.88 (m, 14H), 3.96 – 4.01 (m, 2H), 4.30 (d, *J* = 2.9 Hz, 2H), 4.39 (s, 2H), 4.46 (d, *J* = 2.8 Hz, 2H), 7.22 (dd, *J* = 8.3, 4.8 Hz, 2H), 8.27–8.33 (m, 4H), 8.83 (d, *J* = 2.5 Hz, 2H), 9.52 (s, 2H),

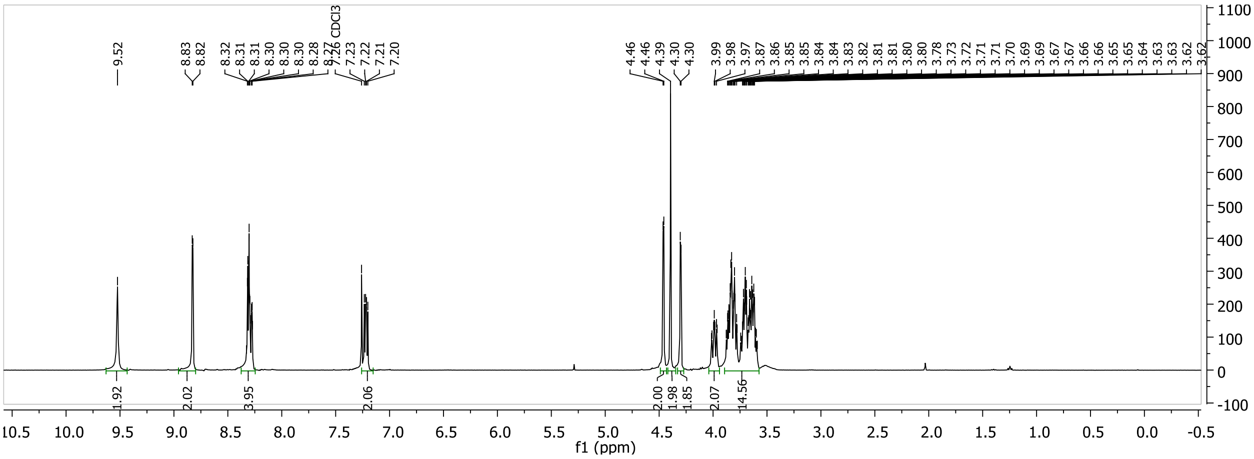

^{13}C NMR (101 MHz, $CDCl_3$): δ/ppm = 67.29 (2CH_2), 68.69 (2CH_2), 69.26 (2CH_2), 70.13 (2CH_2), 82.86 (2CH), 88.61 (2CH_2), 123.57 (2CH), 127.07 (2CH), 134.80 (2C), 142.13 (2CH), 145.17(2CH), 156.28 (2C), 168.20 (2C).

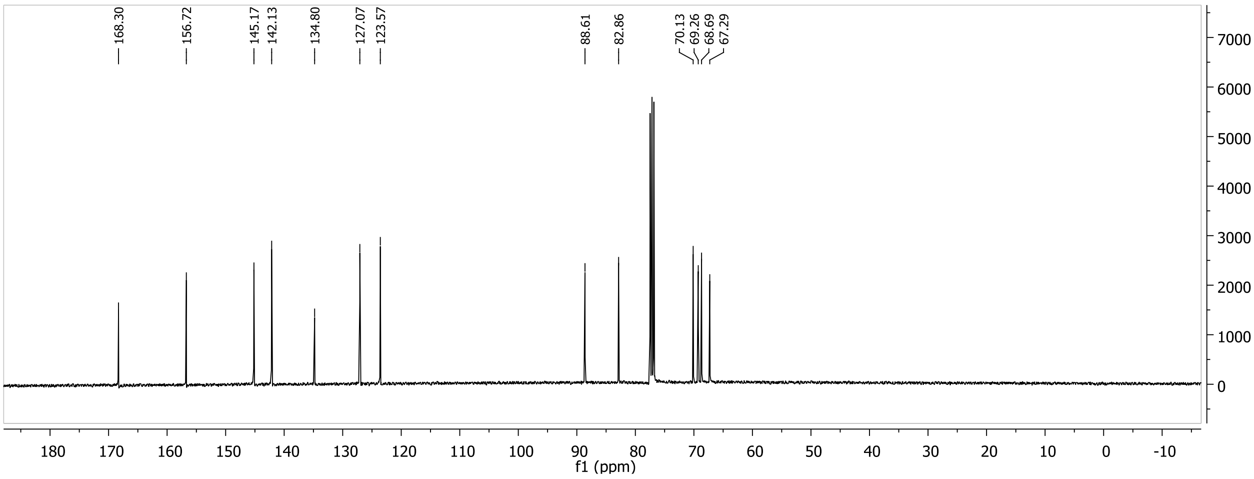

IR (neat): $\tilde{\nu}$ /cm^{-1} = 3328 2936, 2898, 2936, 1687, 1643, 1587, 1519, 1483, 1444, 1425, 1298, 1215, 1130, 1094, 1067, 1049, 1025, 977, 953, 912, 845, 793, 706, 676, 653, 618.

HR-ESI: m/z = 529.2290 $[M+H]^+$ (calculated for $C_{26}H_{33}N_4O_8$ m/z = 529.2293)

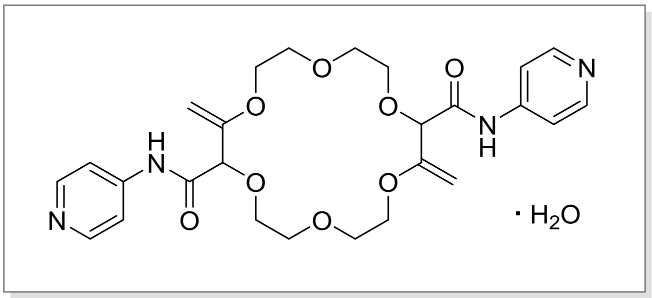

4l. Yield: 50 % of yellow solid (65 mg)

X-ray crystallized from CH_2Cl_2/ heptane (see crystallographic section)

R*f* : 0.23 (silica gel, mobile phase MeOH/ CH_2Cl_2 5/95), (using preparative silica TLC MeOH/Et_3N/ CH_2Cl_2 : 10/10/80)

^{1}H NMR (400 MHz, $CDCl_3$): δ/ppm = 3.58– 3.78 (m, 8H, and H_2O-2H), 3.84 – 3.91 (m, 6H),

3.98 – 4.03 (m, 2H), 4.34 (d, *J* = 2.9 Hz, 2H), 4.39 (s, 2H), 4.49 (d, *J* = 2.9 Hz, 2H), 7.50 – 7.51 (m, 4H), 8.38 – 8.40 (m, 4H), 9.39 (s, 2H).

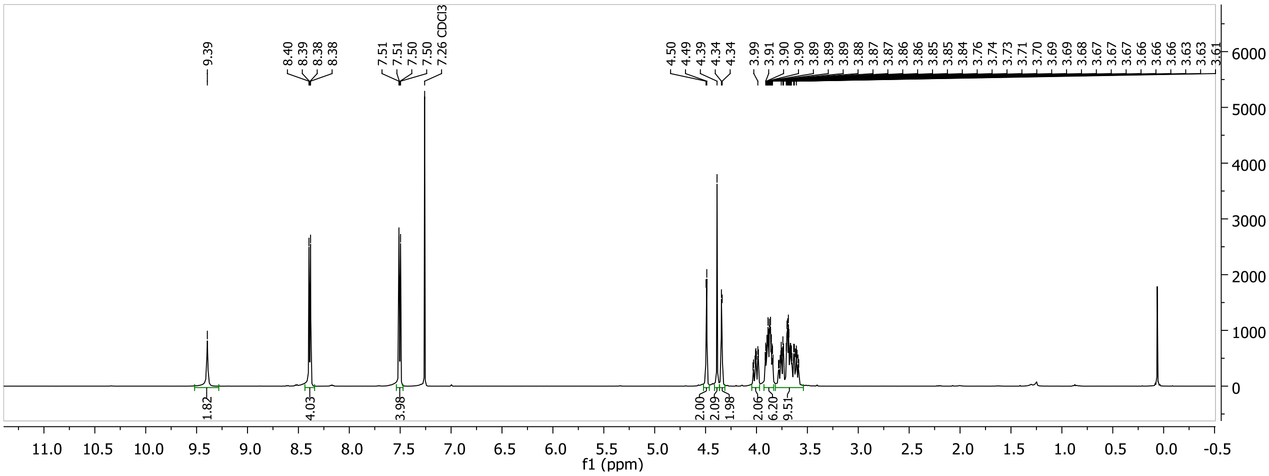

^{13}C NMR (101 MHz, $CDCl_3$): δ/ppm = 66.87 ($2CH_2$), 68.83 ($2CH_2$), 68.90 ($2CH_2$), 70.02 ($2CH_2$), 82.84 (2CH), 88.85 ($2CH_2$), 113.70 (4CH), 144.65 (2C), 150.79 (4CH), 156.31 (2C), 168.48 (2C),

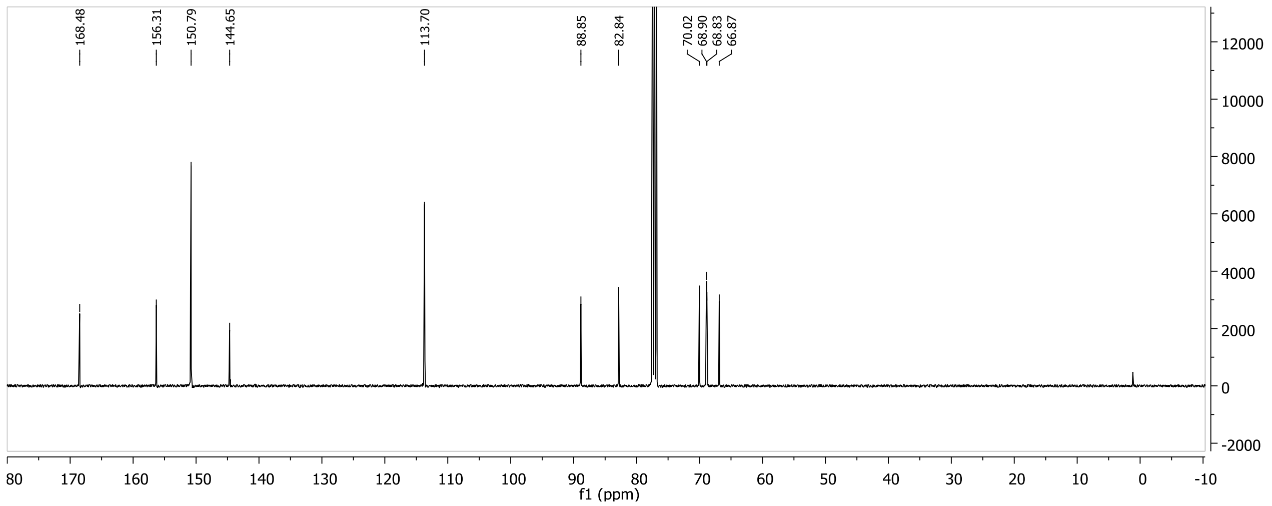

IR (neat): $\tilde{\nu}$ /cm^{-1} = 3518, 3285, 2928, 2877, 1702, 1631, 1588, 1511, 1464, 1418, 1334, 1298, 1215, 1130, 1094, 1067, 1049, 1025, 977, 953, 912, 845, 793, 706, 676, 653, 618.

HR-ESI: m/z = 529.2299 $[M+H]^+$ (calculated for $C_{26}H_{33}N_4O_8$ m/z = 529.2293)

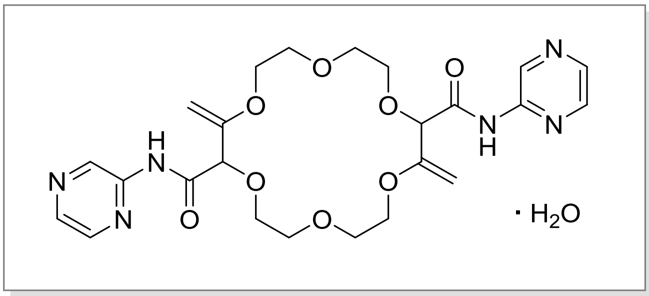

4m. Yield: 48 % of yellow solid (63 mg)

R*f* : 0.6 (silica gel, mobile phase MeOH/ CH_2Cl_2 5/95), (using preparative silica TLC MeOH/Et_3N/ CH_2Cl_2 : 10/5/85)

^{1}H NMR (400 MHz, $CDCl_3$): δ/ppm = 3.60 – 3.66 (m, 2H), 3.72 – 3.86 (m, 10H and H_2O-2H), 3.91 – 4.02 (m, 4H), 4.31 (d, *J* = 2.8 Hz, 2H), 4.42 (d, *J* = 2.8 Hz, 2H), 4.51 (s, 2H), 8.20 (dd, *J* = 2.5, 1.6 Hz, 2H), 8.29 (d, *J* = 2.5 Hz, 2H), 9.42 (s, 2H), 9.51 (d, *J* = 1.5 Hz, 2H).

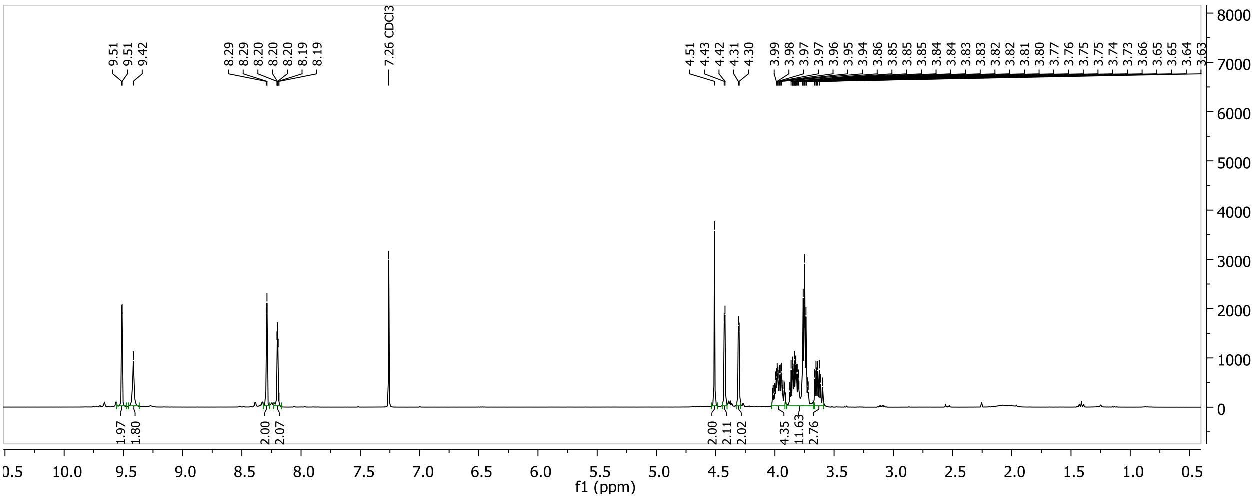

^{13}C NMR (101 MHz, $CDCl_3$): δ/ppm = 67.69 ($2CH_2$), 68.60 ($2CH_2$), 69.10 ($2CH_2$), 70.42 ($2CH_2$), 82.01 (2CH), 87.80 ($2CH_2$), 137.31 (2CH), 140.39 (2CH), 142.32 (2CH), 147.98 (2C), 156.71 (2C), 168.35 (2C).

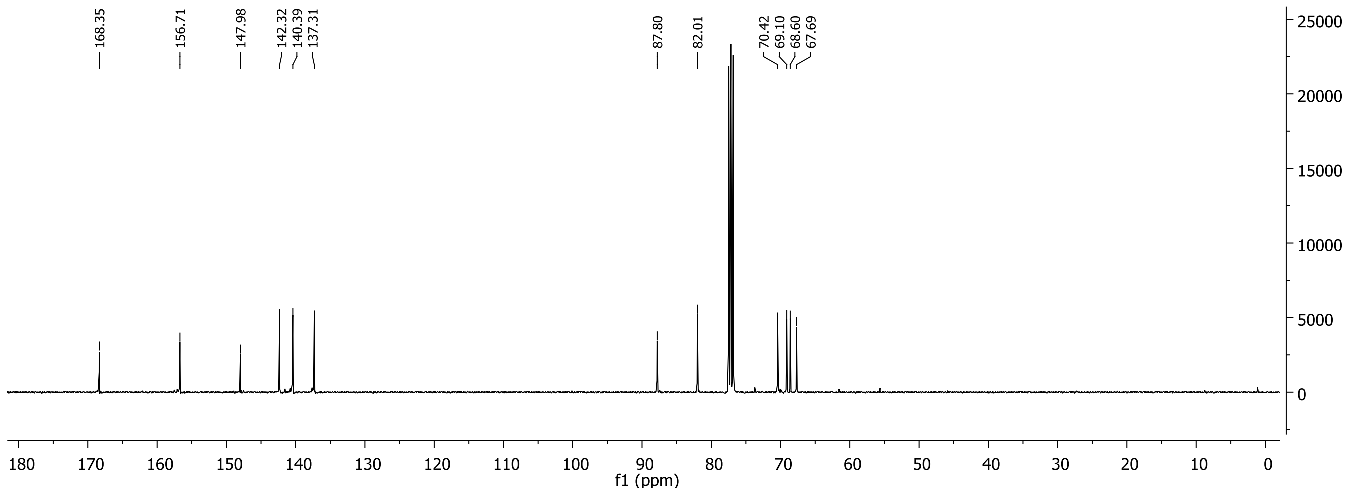

IR (neat): $\tilde{\nu}$ /cm^{-1} = 3296, 3122, 2925, 1689, 1631, 1637, 1597, 1534, 1505, 1473, 1292, 1135, 1092, 1074, 1010, 992, 929, 899, 819, 690, 641, 623, 555.

HR-ESI: m/z = 531.2209 $[M+H]^+$ (calculated for $C_{26}H_{33}N_4O_8$ m/z = 531.2198)

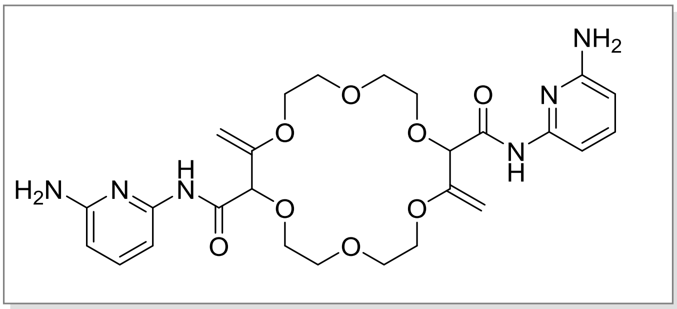

4n. Yield: 30 % of yellow solid (42 mg)

R*f* : 0.2 (silica gel, mobile phase MeOH/ CH_2Cl_2 10/90), (using preparative silica TLC MeOH/Et_3N/ CH_2Cl_2 : 5/5/90)

^{1}H NMR (400 MHz, CD_3CN): δ/ppm = 3.58 – 3.76 (m, 12H), 3.84 – 3.93 (m, 4H), 4.32 (d, *J* = 2.4 Hz, 2H), 4.35 (d, *J* = 2.4 Hz, 2H), 4.38 (s, 2H), 4.82 (bs, 4H), 6.26 (dd, *J* = 7.9, 0.8 Hz, 2H), 7.35 (dd, *J* = 7.8, 0.8 Hz, 2H), 7.42 (t, *J* = 7.9 Hz, 2H), 8.73 (s, 2H).

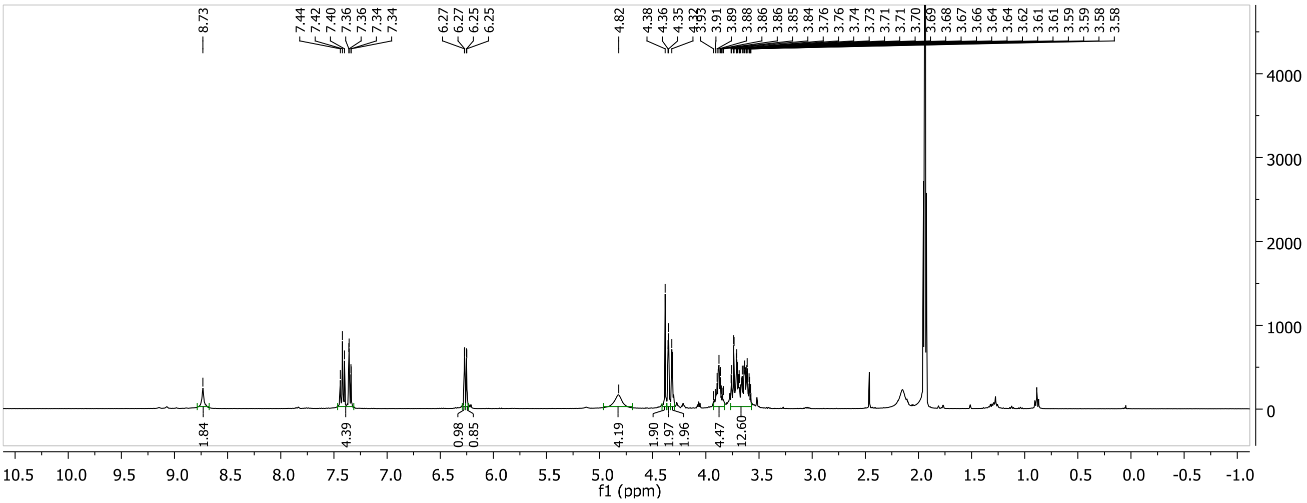

^{13}C NMR (101 MHz, CD_3CN): δ/ppm = 68.81 (2CH_2), 69.54 (2CH_2), 69.57 (2CH_2), 71.16 (2CH_2), 82.77 (2CH), 88.38 (2CH_2), 102.58 (2CH), 105.13 (2CH), 140.52 (2CH), 150.35 (2C), 158.30 (2C), 159.35 (2C), 168.18 (2C).

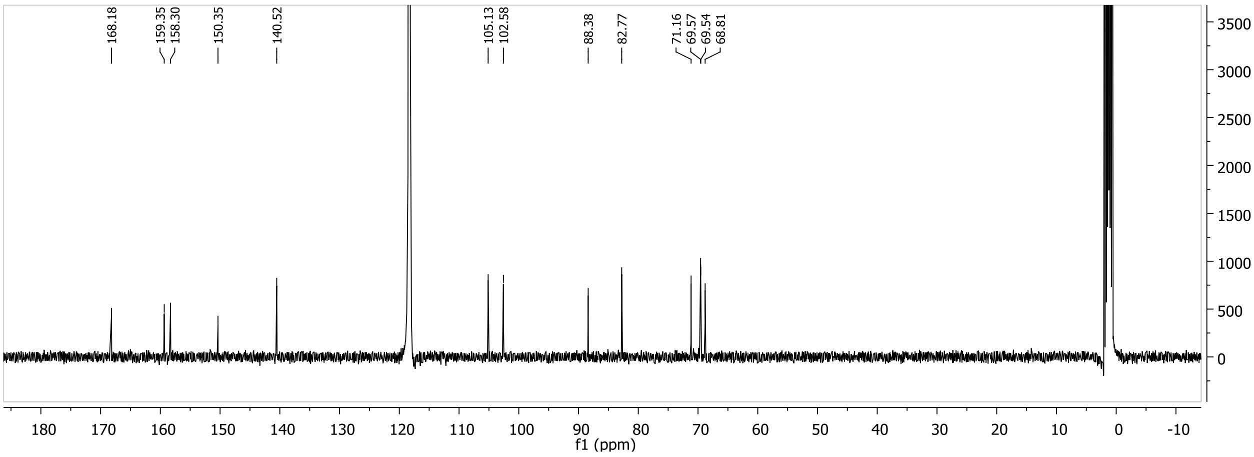

IR (neat): $\tilde{\nu}$ /cm^{-1} = 3387, 3206, 2925, 1694, 1610, 1529, 1455, 1295, 1235, 1082, 986, 835, 784, 667, 544.

HR-ESI: m/z = 559.2513 $[M+H]^+$ (calculated for $C_{26}H_{35}N_6O_8$ m/z = 559.2514)

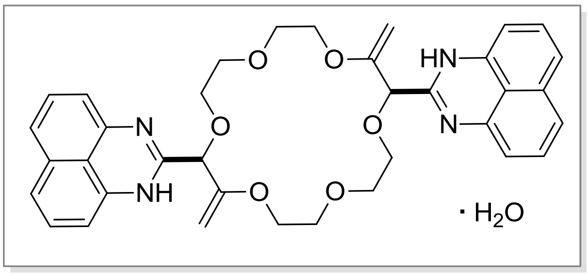

4o. Yield: 64 % of yellow solid (197 mg)

R*f* : 0.22 (silica gel, mobile phase CH_2Cl_2/EtOAc/MeOH/Et_3N 5/5/1/0.1)

^{1}H NMR (300 MHz, $CDCl_3$): δ/ppm = 3.19 (bs, H_2O), 3.58 – 3.59 (m, 4H), 3.74 – 3.85 (m, 8H), 3.98 – 4.28 (m, 4H), 4.28 (d, *J* = 2.7 Hz, 2H), 4.44 (s, 2H), 4.50 (d, *J* = 2.7 Hz, 2H), 6.52 (dd, *J* = 5.8 Hz, 2.4 Hz, 2H), 6.71 (d, *J* = 7.3 Hz, 2H), 6.98 – 7.02 (m, 4H), 7.05 – 7.08 (m, 2H), 7.15 – 7.20 (m, 2H), 10.61 (s, 2H).

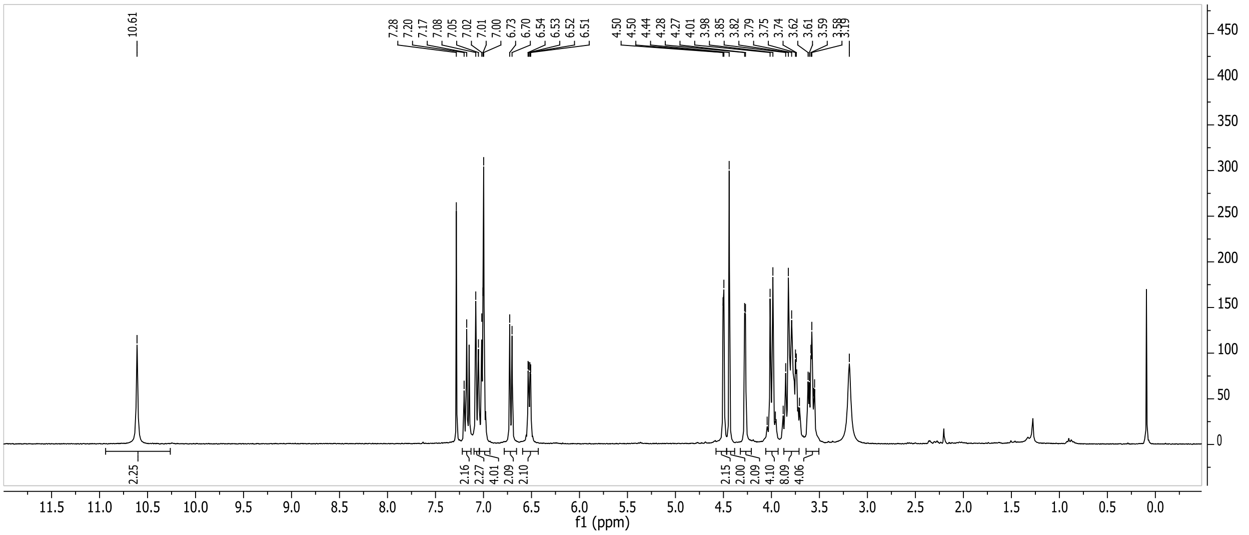

^{13}C NMR (101 MHz, $CDCl_3$): δ/ppm = 66.4 (2CH_2), 67.6 (2CH_2), 69.7 (2CH_2), 70.7 (2CH_2), 83.4 (2CH), 88.5 (2CH_2), 102.7 (2CH), 114.0 (2CH), 118.3 (2CH), 120.0 (2CH), 123.08 (2C), 127.9 (2CH), 128.9 (2CH), 135.7 (2C), 138.2 (2C), 145.1 (2C), 154.8 (2C), 156.4 (2C).

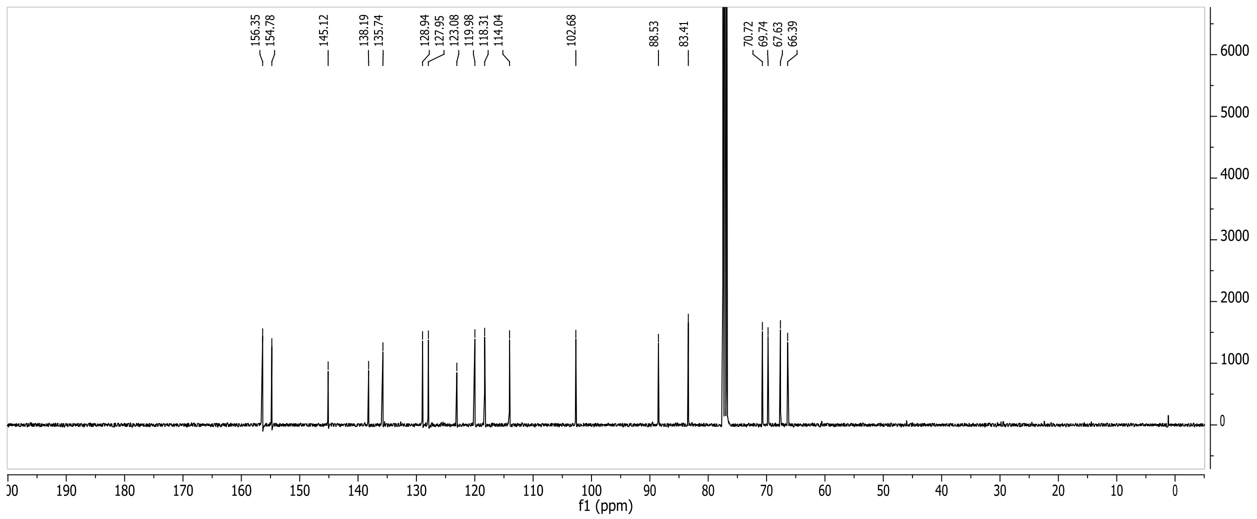

IR (neat): $\tilde{\nu}$ /cm^{-1} = 3202, 3052, 2922, 1632, 1607, 1592, 1536, 1477, 1443, 1412, 1371, 1340, 1285, 1163, 1095, 1073, 993, 931, 906, 822, 770, 726.

HR-ESI: m/z = 621.2687 $[M+H]^+$ (calculated for $C_{36}H_{37}N_4O_6$ m/z = 621.2708)

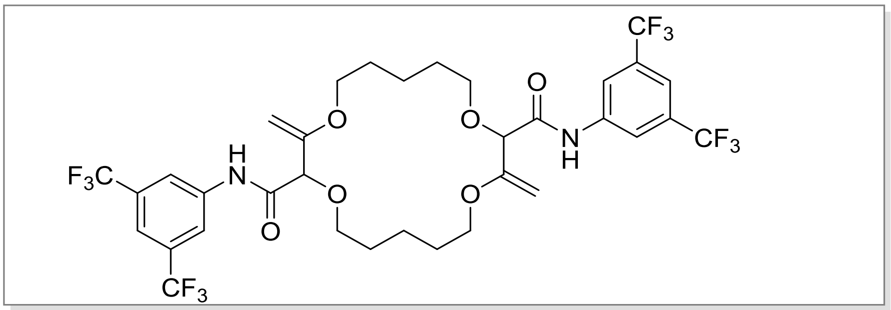

8a. Yield: 60 % of off-white solid (120 mg)

X-ray crystallized from CH_2Cl_2/ heptane (see crystallographic section)

M.p. 104 °C - 105 °C (crystallized from CH_2Cl_2 / Pentane)

R*f* : 0.57 (silica gel, mobile phase 30% EtOAc/Pentane), (using column chromatography on silica gel).

^{1}H NMR (400 MHz, $CDCl_3$): δ/ppm = 1.42 – 1.55 (m, 2H), 1.62 – 1.87 (m, 10H), 3.59 –3.61 (m, 4H), 3.72 –3.78 (m, 2H), 3.84 – 3.89 (m, 2H), 4.30 (s, 2H), 4.32– 4.35 (s, 4H), 7.58 (s, 2H), 8.04 (s, 4H), 8.63 (s, 2H).

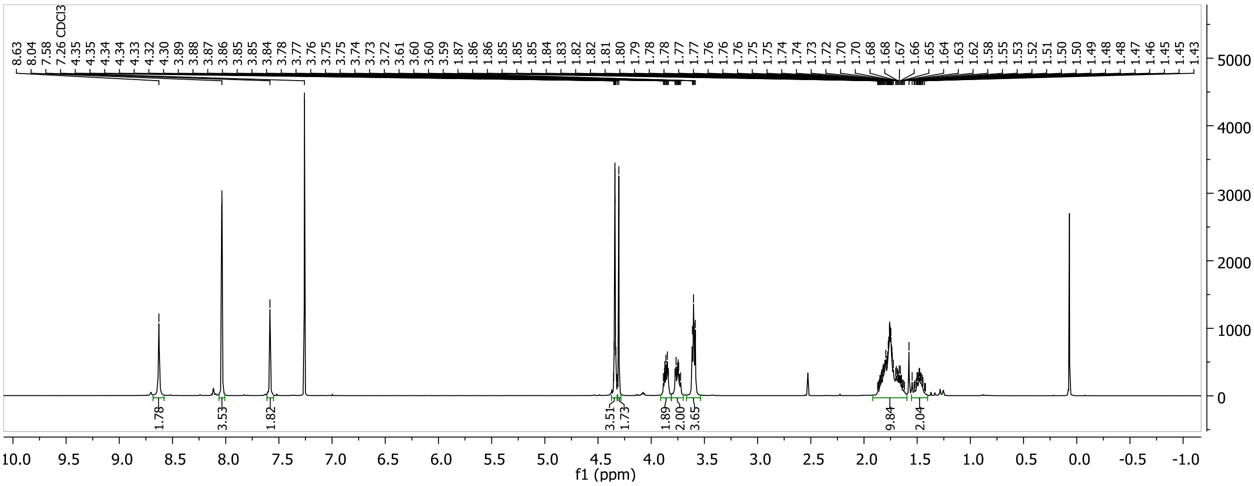

^{13}C NMR (101 MHz, $CDCl_3$): δ/ppm = 24.37 ($2CH_2$), 28.69 ($2CH_2$), 29.74 ($2CH_2$), 68.22 ($2CH_2$), 69.85 ($2CH_2$), 82.65 (2CH), 88.87 ($2CH_2$), 117.85 (2CH), 119.47 (4CH), 123.15 (q, J = 272.9 Hz $4CF_3$), 132.48 (q, J = 33.6 Hz 4C), 138.75 (2C), 156.30 (2C), 167.90 (2C).

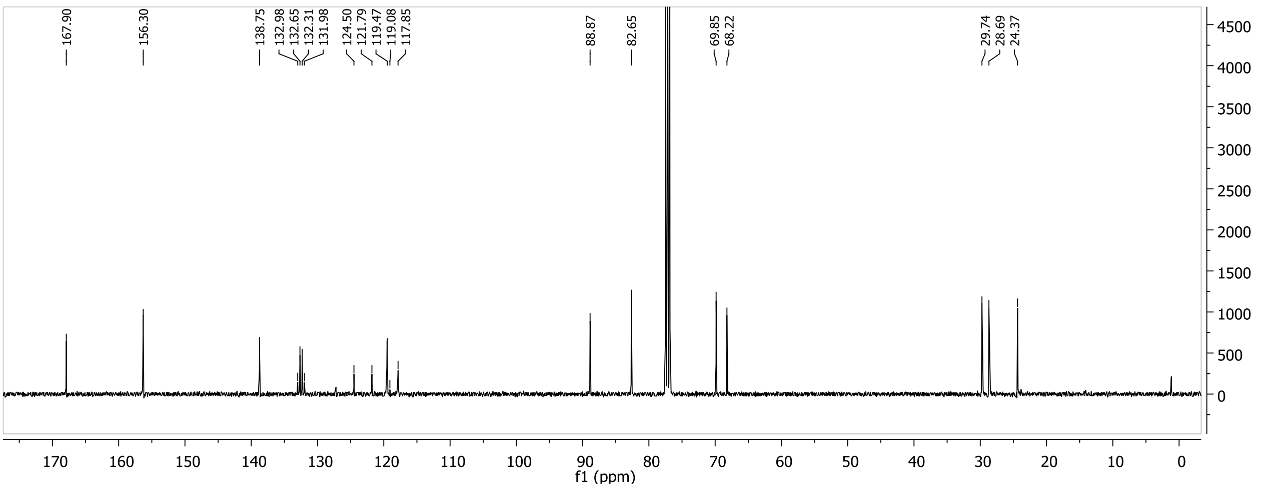

^{19}F NMR (282 MHz, $CDCl_3$): δ/ppm = -62.51 (s, 12F).

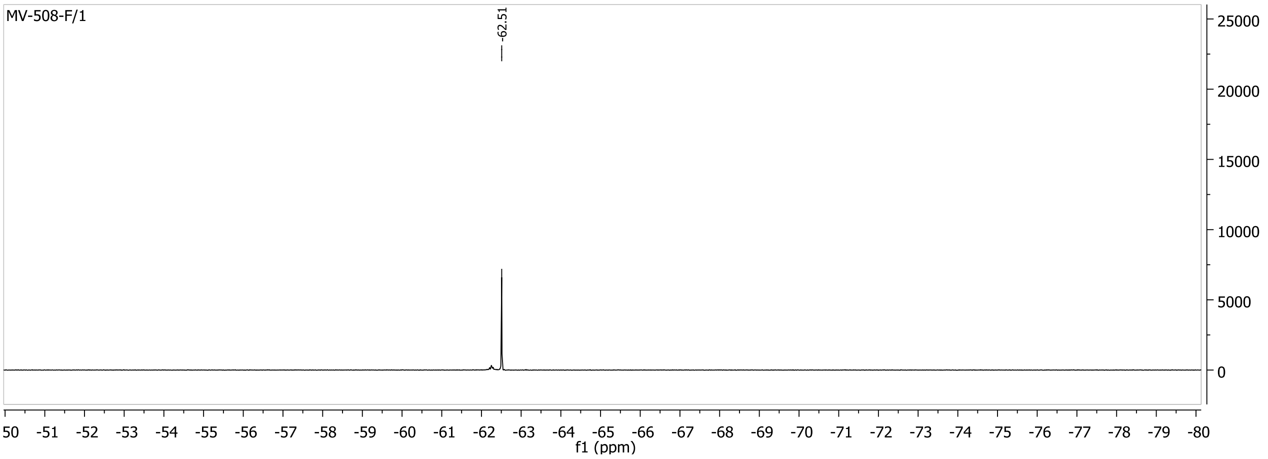

IR (neat): $\tilde{\nu}$ /cm^{-1} = 3309, 2939, 1685, 1626, 1539, 1473, 1441, 1379, 1274, 1169, 1120, 936, 886, 845, 700, 680, 568.

HR-ESI: m/z = 812.2603 $[M+NH4]^+$ (calculated for $C_{34}H_{38}F_{12}N_3O_6$ m/z = 812.2564)

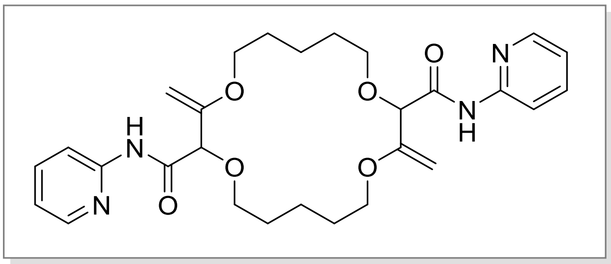

8j. Yield: 50% of pale yellow non crystalline solid (65 mg)

R*f* : 0.57 (silica gel, mobile phase MeOH/ CH_2Cl_2 5/95), (using preparative silica TLC MeOH/Et_3N/ CH_2Cl_2 : 5/5/90)

^{1}H NMR (400 MHz, $CDCl_3$): δ/ppm = 1.36 – 1.47 (m, 2H), 1.49 – 1.59 (m, 2H), 1.61 – 1.84 (m, 8H), 3.49 – 3.55 (m, 4H), 3.67 – 3.72 (m, 2H), 3.79 – 3.84 (m, 2H), 4.26 (s, 2H), 4.28 (d, *J* = 2.6 Hz, 2H), 4.29 (d, *J* = 2.4 Hz, 2H), 6.99 – 7.02 (m, 2H), 7.65 – 7.70 (m, 2H), 8.22 – 8.26 (m, 4H), 9.16 (s, 2H).

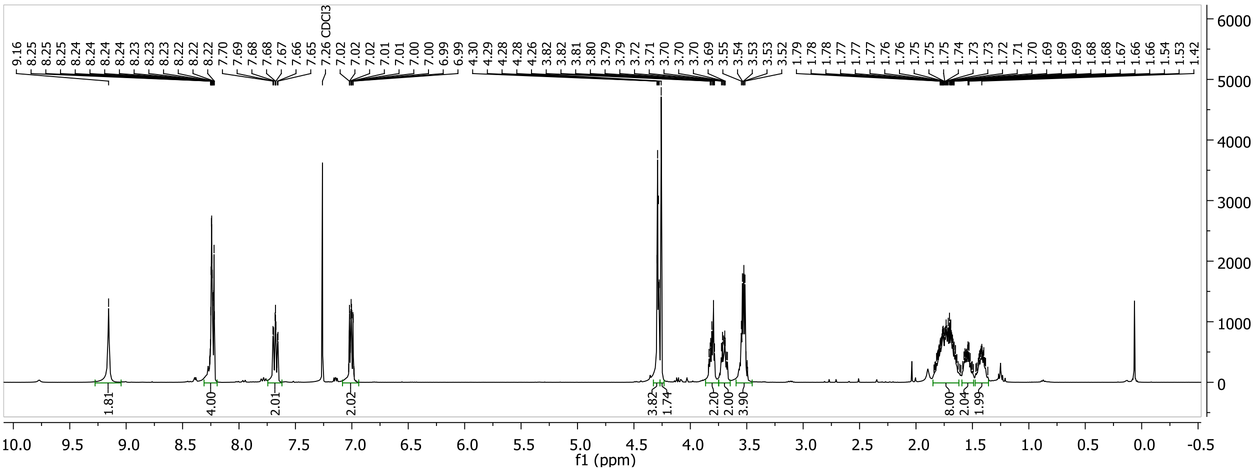

^{13}C NMR (101 MHz, $CDCl_3$): δ/ppm = 24.09 (2CH_2), 28.64 (2CH_2), 29.75 (2CH_2), 68.00 (2CH_2), 69.23 (2CH_2), 82.41 (2CH), 87.95 (2CH_2), 114.32 (2CH), 119.94 (2CH), 138.24 (2CH), 148.04 (2CH), 150.98 (2C), 157.07 (2C), 168.14 (2C).

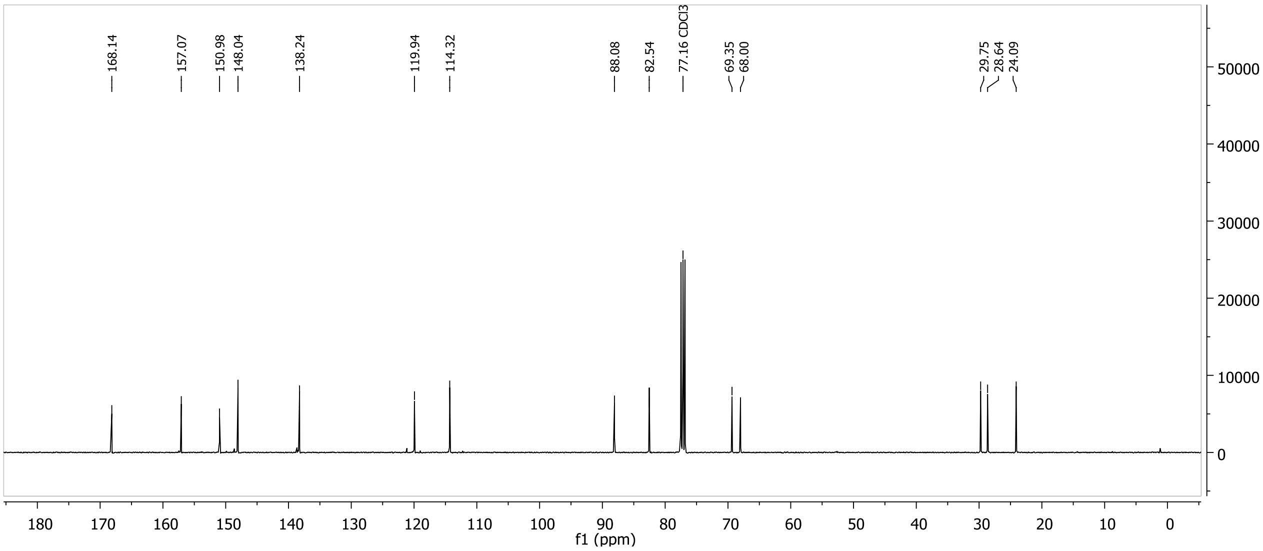

IR (neat): $\tilde{\nu}$ /cm^{-1} = 3385, 2928, 2866, 1694, 1632, 1575, 1512, 1432, 1298, 1225, 1082, 914, 777, 729, 621, 570.

HR-ESI: m/z = 525.2705 [M+H]$^+$ (calculated for $C_{26}H_{33}N_4O_6$ m/z = 525.2708)

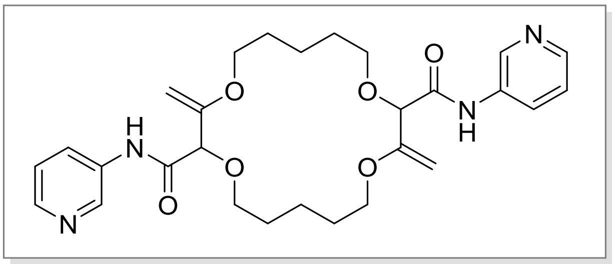

8k. Yield : 60% of pale yellow solid (78 mg)

M.p. 128 °C - 130 °C (crystallized from CH_2Cl_2 / Heptane)

R*f* : 0.24 (silica gel, mobile phase MeOH/ CH_2Cl_2 5/95), (using preparative silica TLC MeOH/Et_3N/ CH_2Cl_2 : 5/5/90)

^{1}H NMR (400 MHz, $CDCl_3$): δ/ppm = 1.45 – 1.77 (m, 12H), 3.54 – 3.59 (m, 4H), 3.70 – 3.81 (m, 4H), 4.29 (s, 2H), 4.30 (d, *J* = 2.5 Hz, 2H), 4.34 (d, *J* = 2.5 Hz, 2H), 7.24 – 7.27 (m, 2H), 8.31 – 8.35 (m, 4H), 8.73 (d, *J* = 2.5 Hz, 2H), 9.05 (s, 2H).

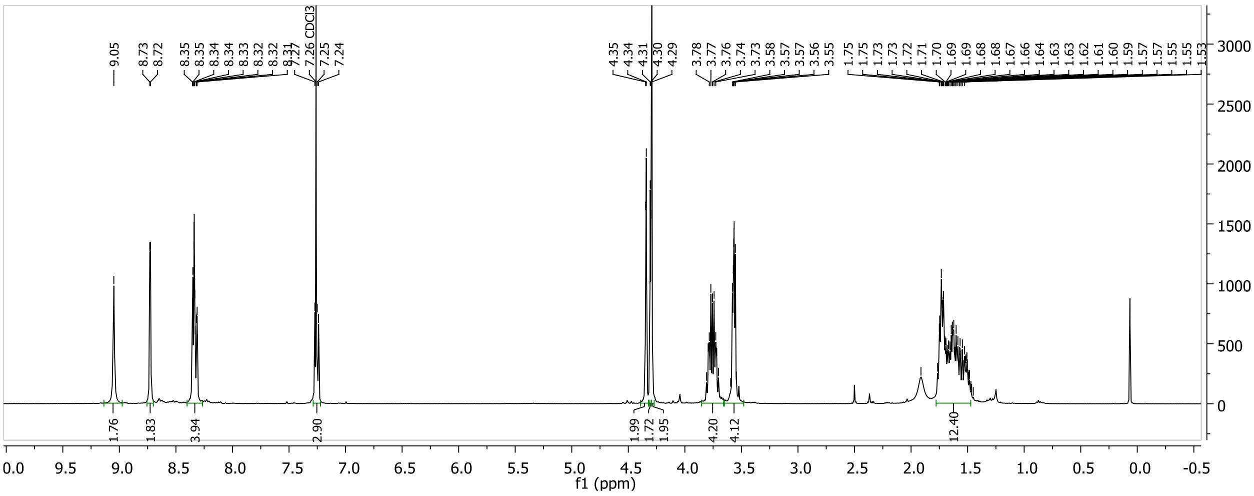

^{13}C NMR (101 MHz, $CDCl_3$): δ/ppm = 24.57 ($2CH_2$), 29.06 ($2CH_2$), 29.82 ($2CH_2$), 68.33 ($2CH_2$), 69.73 ($2CH_2$), 82.93 (2CH), 88.58 ($2CH_2$), 123.74 (2CH), 127.28 (2CH), 134.59 (2C), 141.83 (2CH), 145.12 (2CH), 156.85 (2C), 168.38 (2C).

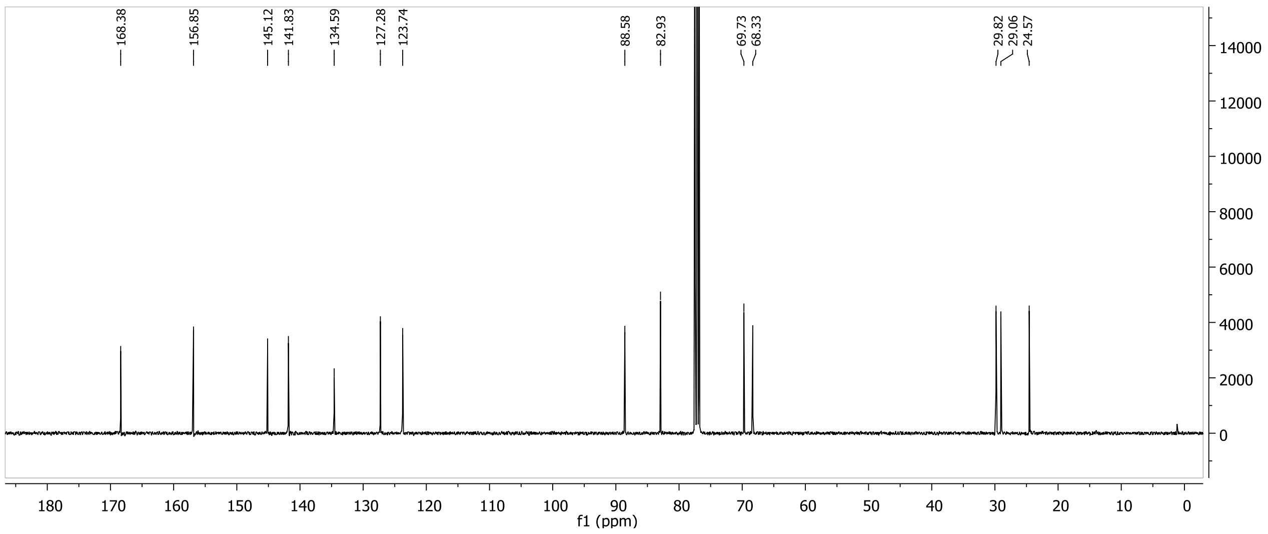

IR (neat): $\tilde{\nu}$ /cm^{-1} = 3241, 2928, 2871, 1685, 1636, 1585, 1523, 1480, 1420, 1330, 1291, 1225, 1084, 939, 804, 705, 620.

HR-ESI: m/z = 525.2704 [M+H]$^+$ (calculated for $C_{26}H_{33}N_4O_6$ m/z = 525.2708).

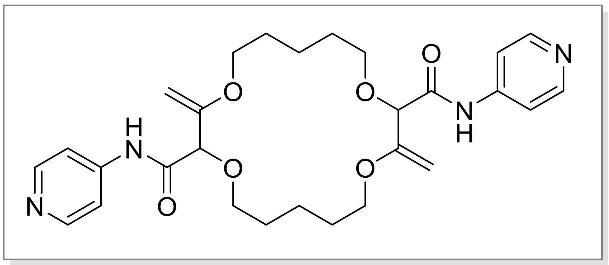

8l. Yield : 55% of pale yellow solid (72 mg)

M.p. 127 °C - 129 °C (crystallized from CH_2Cl_2 / Heptane)

R*f* : 0.122 (silica gel, mobile phase MeOH/ CH_2Cl_2 5/95), (using preparative silica TLC MeOH/Et_3N/ CH_2Cl_2 : 5/5/90)

^{1}H NMR (400 MHz, $CDCl_3$): δ/ppm = 1.39 – 1.49 (m, 2H), 1.60 – 1.84 (m, 10H), 3.51 – 3.60 (m, 4H), 3.68 – 3.75 (m, 2H), 3.81 – 3.88 (m, 2H), 4.26 (s, 2H), 4.33 (s, 4H), 7.47– 7.48 (m, 4H), 8.46 – 8.48 (m, 4H), 8.54 (s, 2H).

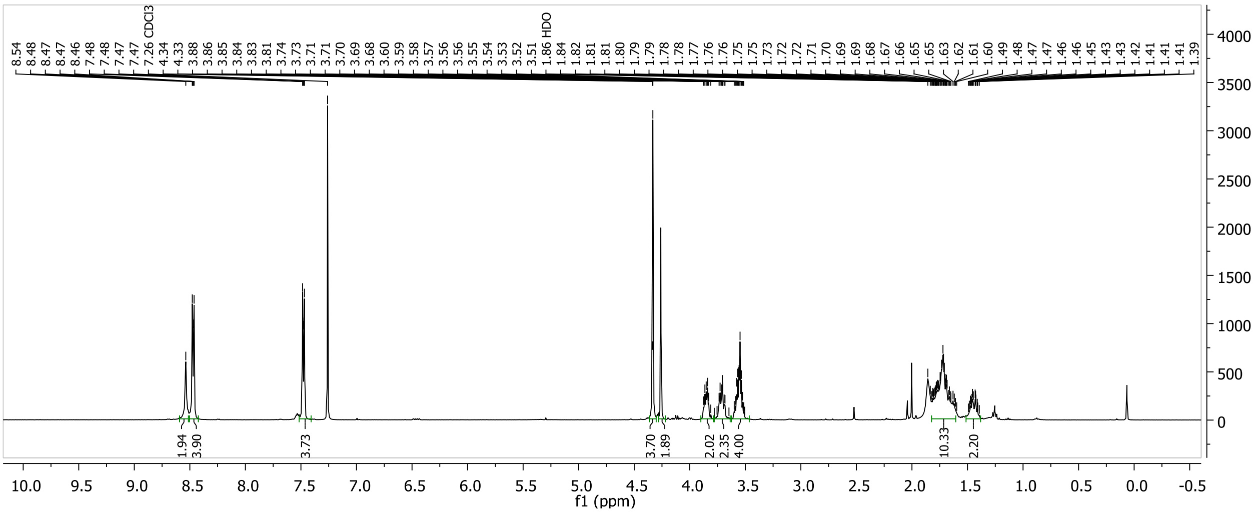

^{13}C NMR (101 MHz, $CDCl_3$): δ/ppm = 24.27 ($2CH_2$), 28.67 ($2CH_2$), 29.77 ($2CH_2$), 68.17 ($2CH_2$), 69.62 ($2CH_2$), 82.59 (2CH), 88.96 ($2CH_2$), 113.64 (4CH), 144.15 (2C), 150.86 (4CH), 156.23 (2C), 168.09 (2C).

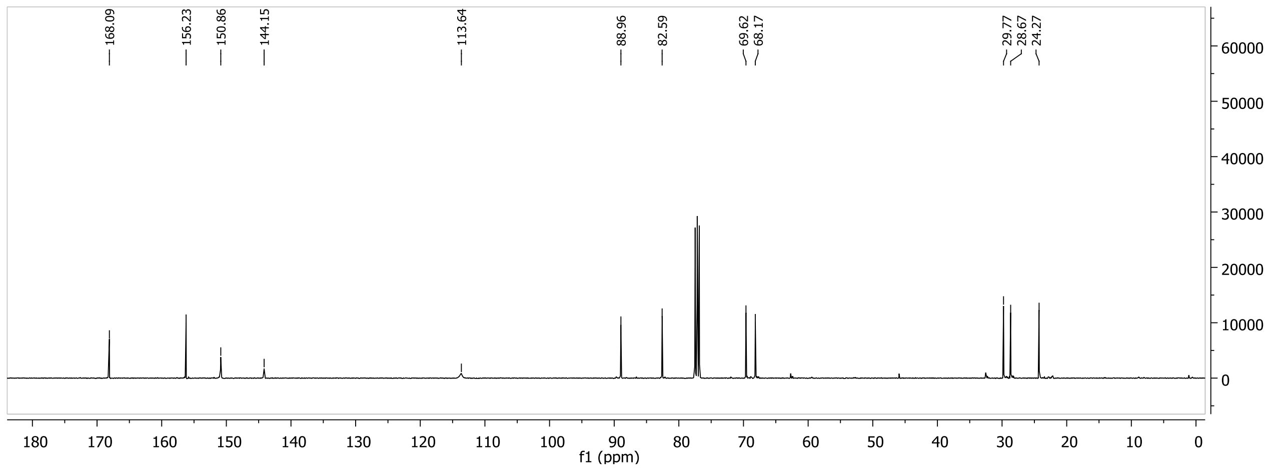

IR (neat): $\tilde{\nu}$ /cm^{-1} = 2926, 2864, 1698, 1585, 1506, 1412, 1330, 1286, 1209, 1084, 999, 822, 578.

HR-ESI: m/z = 525.2702 $[M+H]^+$ (calculated for $C_{26}H_{33}N_4O_6$ m/z = 525.2708)

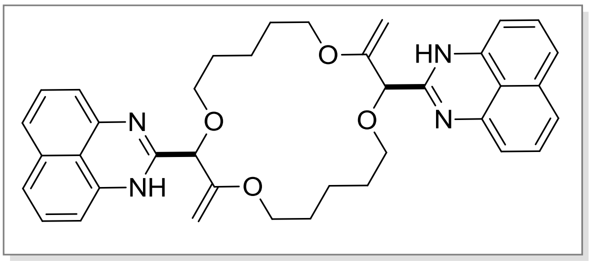

8o. Yield: 48 % of yellow solid (178 mg)

M.p. 130 °C - 134 °C (crystallized from EtOAc)

R*f* : 0.7 (silica gel, mobile phase CH_2Cl_2/EtOAc/MeOH/Et_3N 5/5/1/0.1)

^{1}H NMR (400 MHz, $CDCl_3$): δ/ppm = 1.64 – 1.82 (m, 12H), 3.54 – 3.75 (m, 4H), 3.77 – 3.79 (m, 2H), 3.86 – 3.88 (m, 2H), 4.27 (d, *J* = 2.6 Hz, 2H), 4.40 (s, 2H), 4.43 (d, *J* = 2.6 Hz, 2H), 6.21 – 6.23 (m, 2H), 6.73 (d, *J* = 7.3 Hz, 2H), 6.87 – 6.89 (m, 4H), 7.02 (d, *J* = 8.2 Hz, 2H), 7.14 – 7.16 (m, 2H), 8.01 (s, 2H).

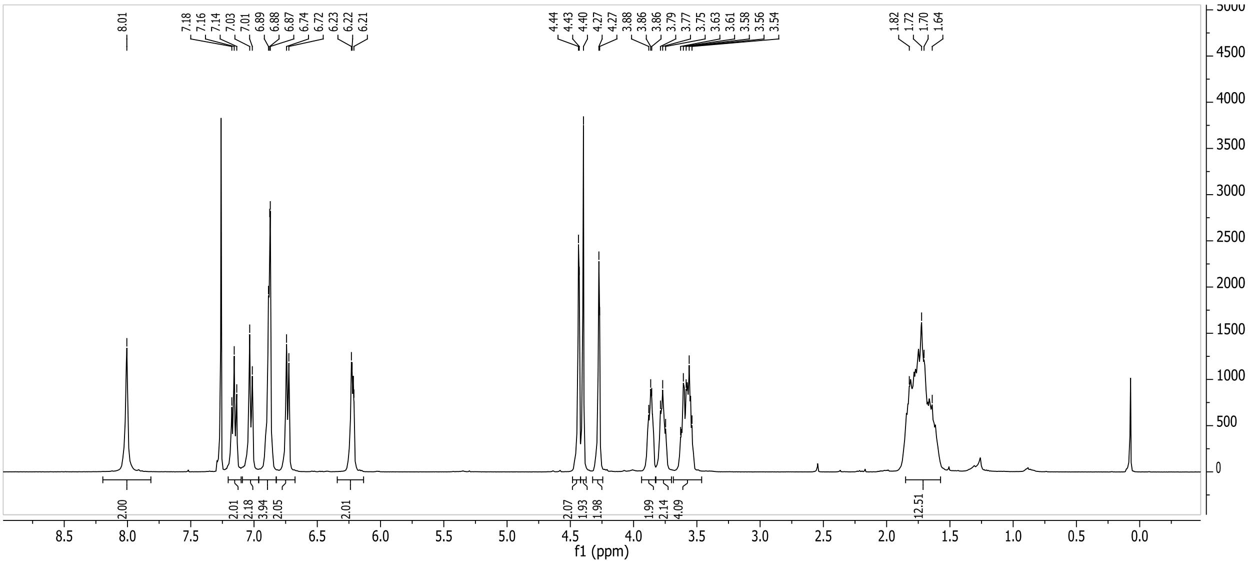

^{13}C NMR (101 MHz, $CDCl_3$): δ/ppm = 24.0 (2CH_2), 28.5 (2CH_2), 29.6 (2CH_2), 68.1 (2CH_2), 68.8 (2CH_2), 82.3 (2CH), 88.1 (2CH_2), 102.2 (2CH), 114.8 (2CH), 118.9 (2CH), 120.4 (2CH), 122.6 (2C), 127.6 (2CH), 128.9 (2CH), 135.5 (2C), 136.8 (2C), 144.4 (2C), 154.2 (2C), 157.1 (2C).

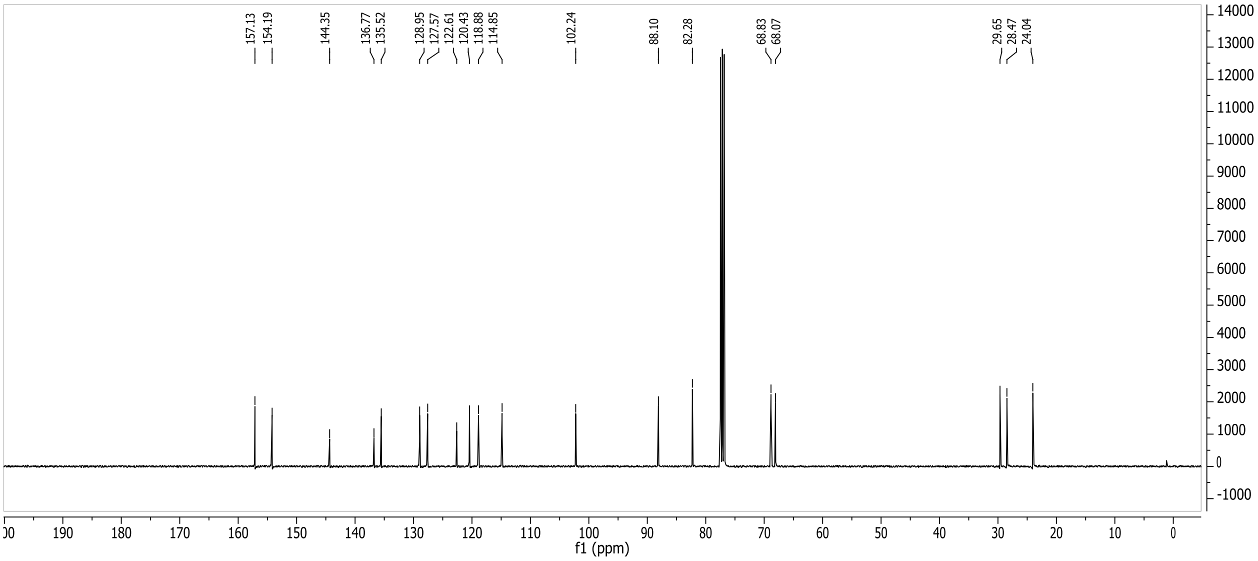

IR (neat): $\tilde{\nu}$ /cm^{-1} = 3390, 3046, 2940, 2869, 1720, 1630, 1594, 1524, 1472, 1443, 1419, 1372, 1341, 1310, 1286, 1230, 1146, 1089, 1033, 994, 976, 932, 891, 860, 825, 814, 771, 758, 732, 691.

HR-ESI: m/z = 617.3106 $[M+H]^+$ (calculated for $C_{38}H_{41}N_4O_4$ m/z = 617.3122)

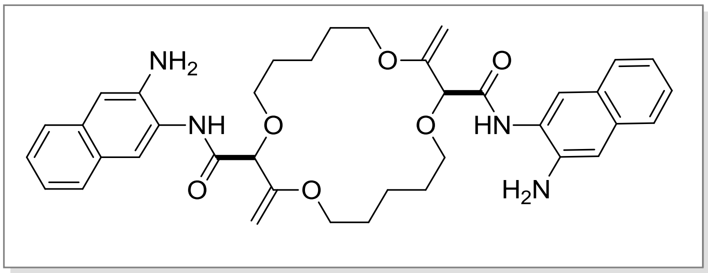

8p. Yield: 41 % of white solid (80 mg)

R_f : 0.65 (silica gel, mobile phase CH_2Cl_2/EtOAc/MeOH/Et_3N 5/5/1/0.1)

^{1}H NMR (400 MHz, $CDCl_3$): δ/ppm = 1.35 – 1.42 (m, 2H), 1.63 – 1.77 (m, 8H), 1.90 – 1.98 (m, 2H), 3.54 – 3.65 (m, 6H), 3.83 (s, 4H- NH_2), 3.89 – 3.92 (m, 2H), 4.33 – 4.37 (m, 6H), 6.48 (s, 2H), 7.02 – 7.13 (m, 6H), 7.44 – 7.46 (m, 2H), 7.76 (s, 2H), 8.85 (s, 2H).

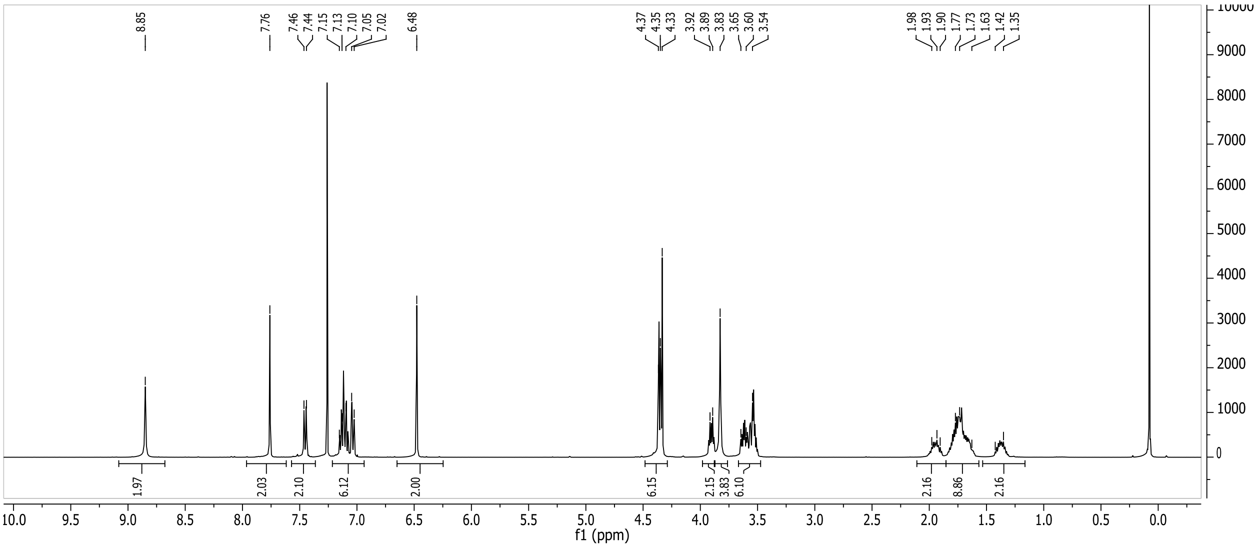

^{13}C NMR (101 MHz, $CDCl_3$): δ/ppm = 24.5 (2CH_2), 29.5 (2CH_2), 30.4 (2CH_2), 68.3 (2CH_2), 68.5 (2CH_2), 82.8 (2CH), 89.5 (2CH_2), 111.9 (2CH), 122.9 (2CH), 123.1 (2CH), 125.4 (2CH), 125.6 (2CH), 125.9 (2C), 127.3 (2CH), 128.4 (2C), 132.7 (2C), 138.8 (2C), 156.3 (2C), 168.0 (2C).

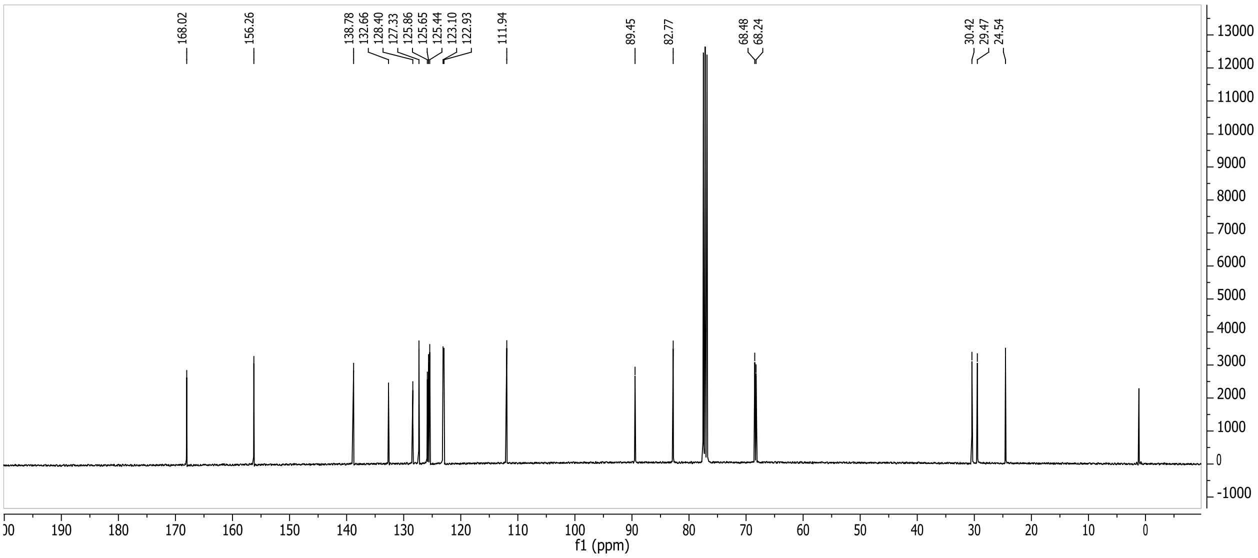

IR (neat): $\tilde{\nu}$ /cm^{-1} = 3396, 3337, 2922, 2866, 1687, 1638, 1580, 1543, 1493, 1474, 1454, 1404, 1347, 1296, 1225, 1141, 1098, 1082, 1004, 947, 908, 878, 866, 832, 813, 728, 687.

HR-ESI: m/z = 653.3322 $[M+H]^+$ (calculated for $C_{38}H_{45}N_4O_6$ m/z = 653.3334)

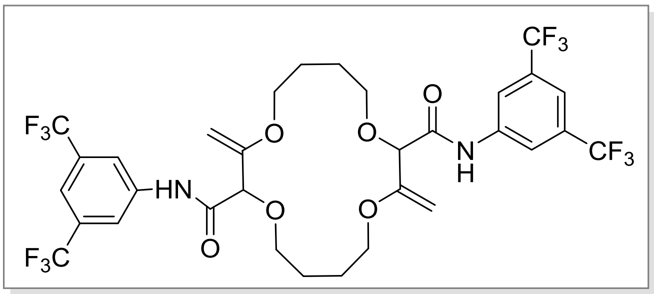

9a. Yield : 88% of white non crystalline solid (182 mg)

M.p. 193 °C - 194 °C (crystallized from CH_2Cl_2 / Pentane)

R*f* : 0.71 (silica gel, mobile phase EtOAc/ Pentane 40/60)

^{1}H NMR (400 MHz, $CDCl_3$): δ/ppm = 1.66 – 1.78 (m, 4H), 1.91 – 2.03 (m, 4H), 3.54 – 3.65 (m, 6H), 3.78 – 3.83 (m, 2H), 4.31 (s, 2H), 4.37 (d, *J* = 2.6 Hz, 2H), 4.40 (d, *J* = 2.6 Hz, 2H), 7.62 (s, 2H), 8.12 (s, 4H), 8.74 (s, 2H).

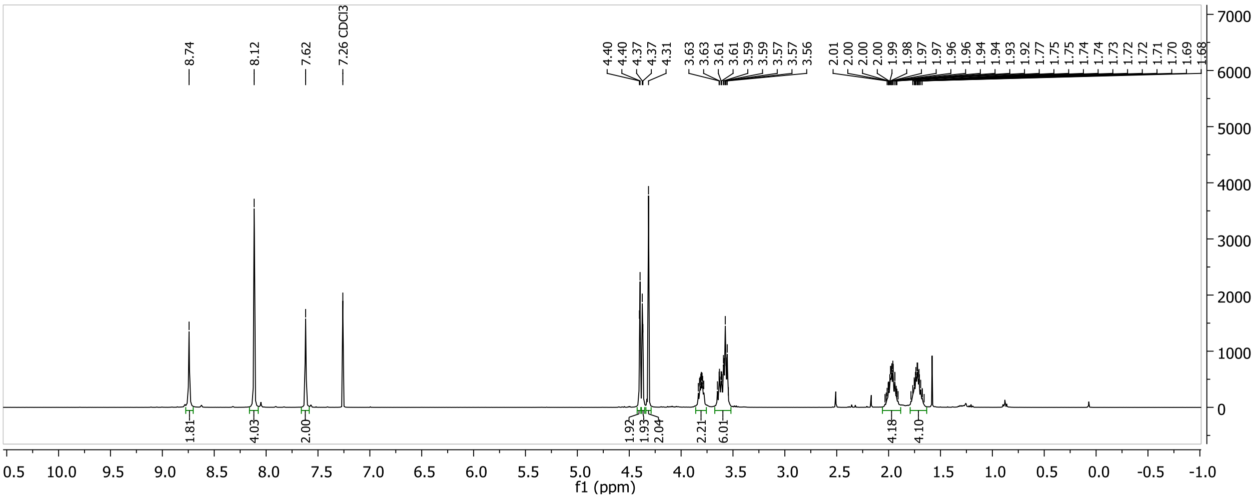

^{13}C NMR (101 MHz, $CDCl_3$): δ/ppm = 24.09 ($2CH_2$), 25.33 ($2CH_2$), 66.41 ($2CH_2$), 67.62 ($2CH_2$), 82.60 (2CH), 89.93 ($2CH_2$), 117.85 (m, 2CH), 119.52 (m, 4CH), 123.21 (q, *J* = 272.8 Hz, 4C), 132.54 (q, *J* = 33.7 Hz, 4C), 138.79 (2C), 158.85 (2C), 167.57 (2C).

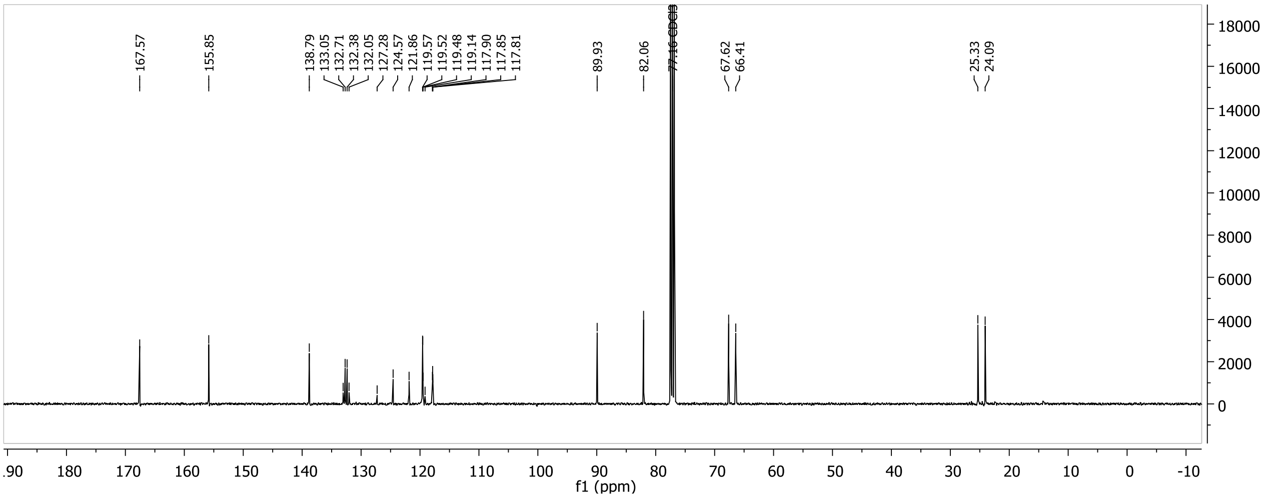

^{19}**F NMR** (282 MHz, $CDCl_3$): δ/ppm = -62.30 (s, 12F)

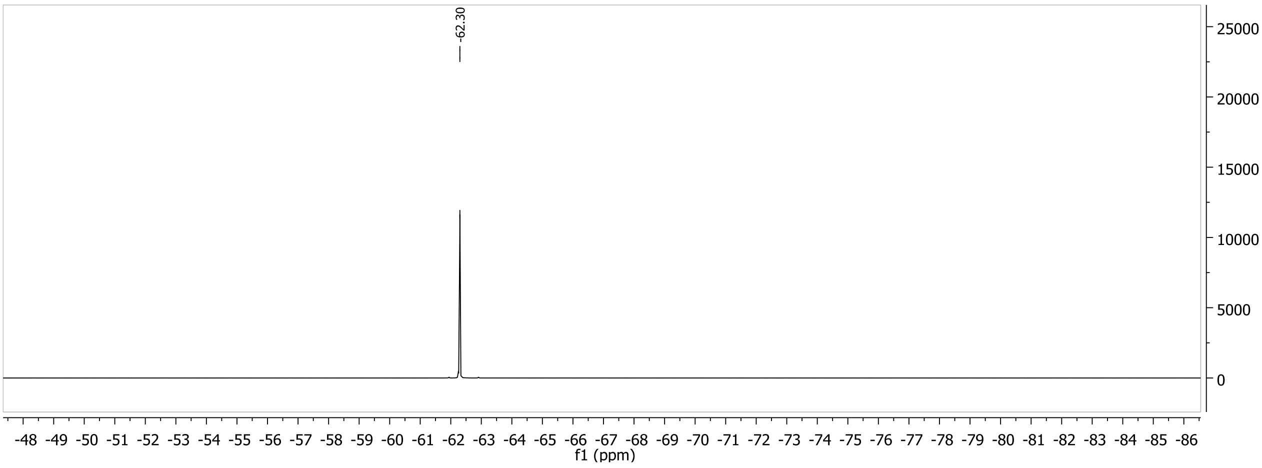

IR (neat): $\tilde{\nu}$ /cm^{-1} = 3292, 2953, 1690, 1621, 1542, 1473, 1440, 1379, 1271, 1174, 1105, 1012, 934, 884, 815, 701, 680.

HR-ESI: m/z = 767.1994 $[M+H]^+$ (calculated for $C_{32}H_{31}N_2F_{12}O_6$ m/z = 767.1985)

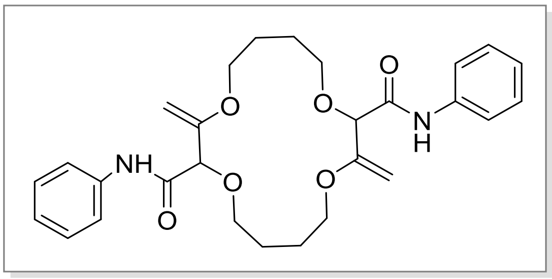

9b. Yield : 53% of white non crystalline solid (71 mg)

R_f : 0.21 (silica gel, mobile phase EtOAc/ Pentane 40/60)

^{1}H NMR (400 MHz, $CDCl_3$): δ/ppm = 1.63 – 1.76 (m, 4H), 1.89 – 2.00 (m, 4H), 3.50 – 3.64 (m, 6H), 3.76 – 3.81 (m, 2H), 4.27 (s, 2H), 4.32 (d, J = 2.5 Hz, 2H), 4.37 (d, J = 2.4 Hz, 2H), 7.10 – 7.14 (m, 2H), 7.31 – 7.36 (m, 4H), 7.59 – 7.62 (m, 4H), 8.48 (s, 2H).

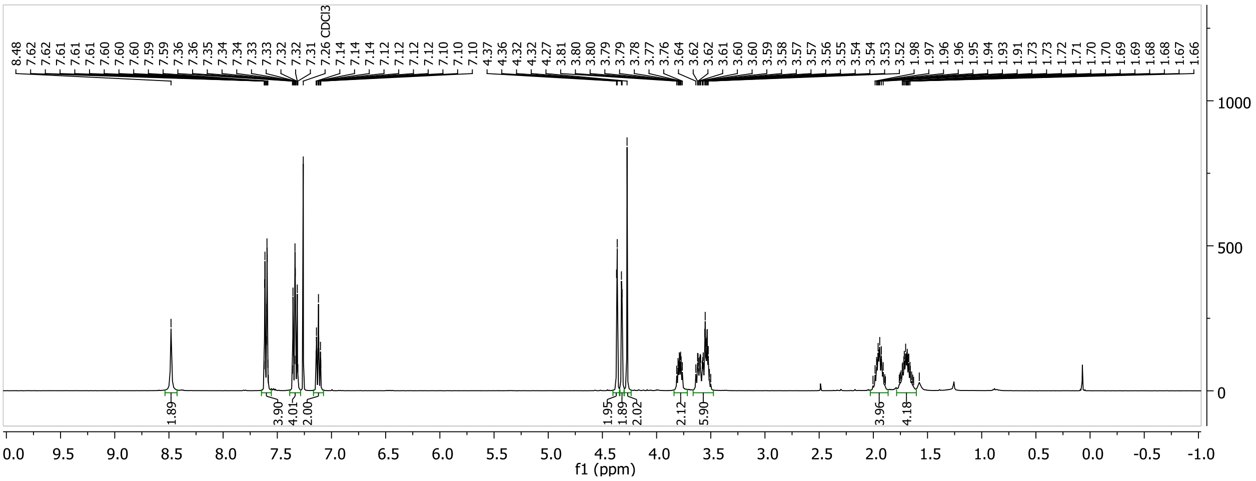

^{13}C NMR (101 MHz, $CDCl_3$): δ/ppm = 24.18 (2CH_2), 25.40 (2CH_2), 66.29 (2CH_2), 67.56 (2CH_2), 82.24 (2CH), 89.24 (2CH_2), 119.81 (2CH), 124.48 (4CH), 129.10 (4CH), 137.50 (2C), 156.47 (2C), 166.88 (2C).

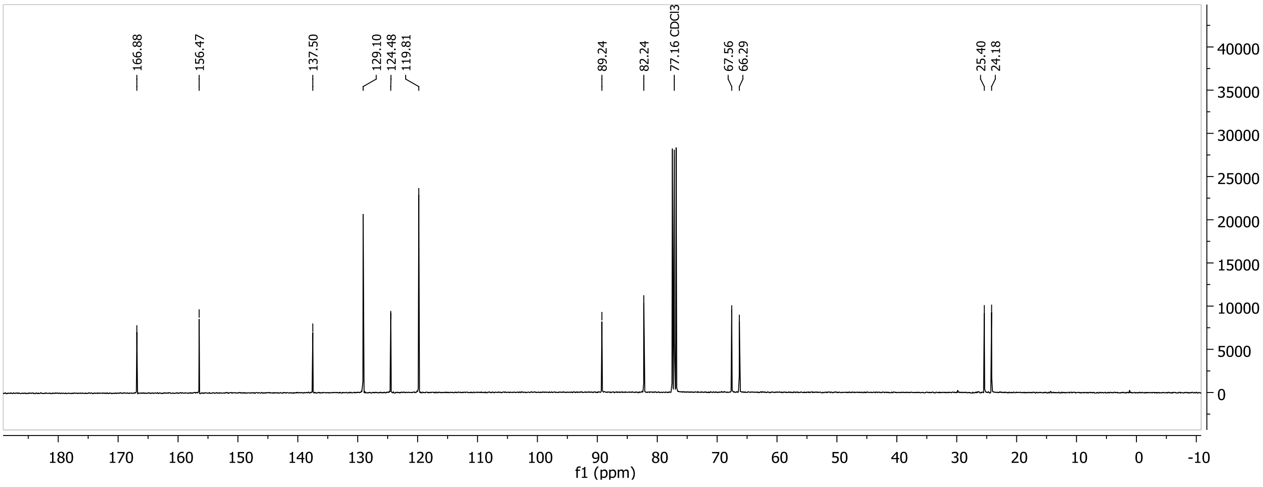

IR (neat): $\tilde{\nu}$ /cm^{-1} = 3291, 2877, 1691, 1624, 1533, 1473, 1441, 1380, 1273, 1173, 1099, 1029, 953, 885, 699, 680.

HR-ESI: m/z = 495.2494 $[M+H]^+$ (calculated for $C_{28}H_{35}N_2O_6$ m/z = 495.2490)

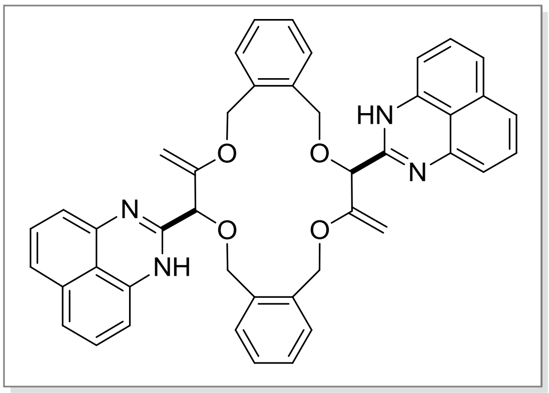

10o. Yield: 36 % of yellow solid (122 mg)

M.p. 166 °C - 170 °C (crystallized from heptane/CH_2Cl_2)

X-ray crystallized from CH_2Cl_2/ heptane (see crystallographic section)

R*f* : 0.66 (silica gel, mobile phase CH_2Cl_2/EtOAc/MeOH/Et_3N 5/5/1/0.1)

^{1}H NMR (400 MHz, $CDCl_3$): δ/ppm = 4.48 – 4.52 (m, 4H), 4.64 – 4.70 (m, 6H), 4.96 – 4.99 (m, 4H), 5.87 (d, *J* = 7.1 Hz, 2H), 6.75 – 6.91 (m, 8H), 7.03 – 7.32 (m, 8H), 7.45 – 7.47 (m, 2H), 8.01 (s, 2H).

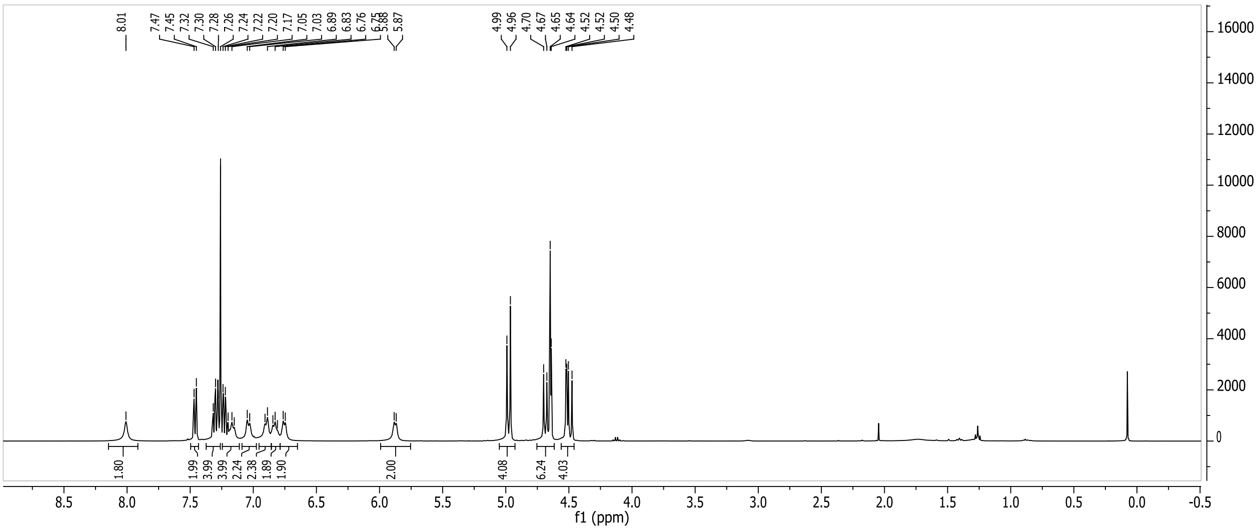

^{13}C NMR (101 MHz, $CDCl_3$): δ/ppm = 67.1 (2CH_2), 68.1 (2CH_2), 81.9 (2CH), 87.9 (2CH_2), 101.2 (2CH), 113.6 (2CH), 117.6 (2C), 119.2 (2CH), 121.5 (2CH), 126.3 (2CH), 127.5 (2CH), 127.8 (2CH), 128.3 (2CH), 128.4 (2CH), 130.0 (2CH), 132.5 (2C), 134.3 (2C), 135.4 (2C), 135.6 (2C), 143.2 (2C), 152.8 (2C), 155.9 (2C).

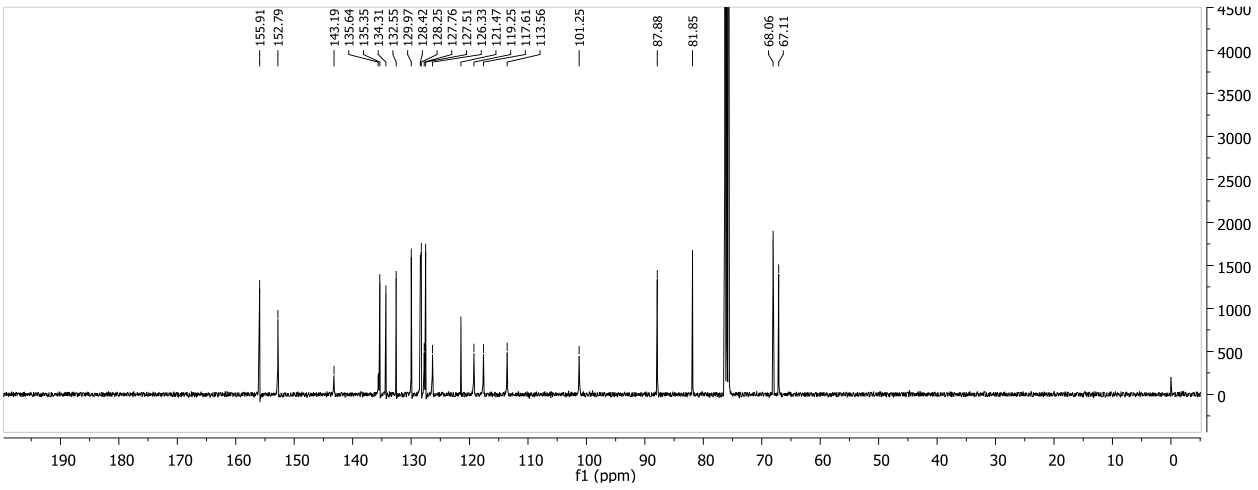

IR (neat): $\tilde{\nu}$ /cm^{-1} = 3394, 3041, 2888, 1630, 1613, 1592, 1523, 1474, 1445, 1424, 1406, 1372, 1339, 1286, 1230, 1215, 1191, 1161, 1124, 1043, 967, 906, 846, 823, 769, 749, 726, 677.

HR-ESI: m/z = 685.2808 $[M+H]^+$ (calculated for $C_{44}H_{37}N_4O_4$ m/z = 685.2809)

4. Enantiodifferentiation of 4a using Eu(hfc)$_3$ as NMR chiral solvating agent (^{1}H NMR, 400 MHz, $CDCl_3$)[1]

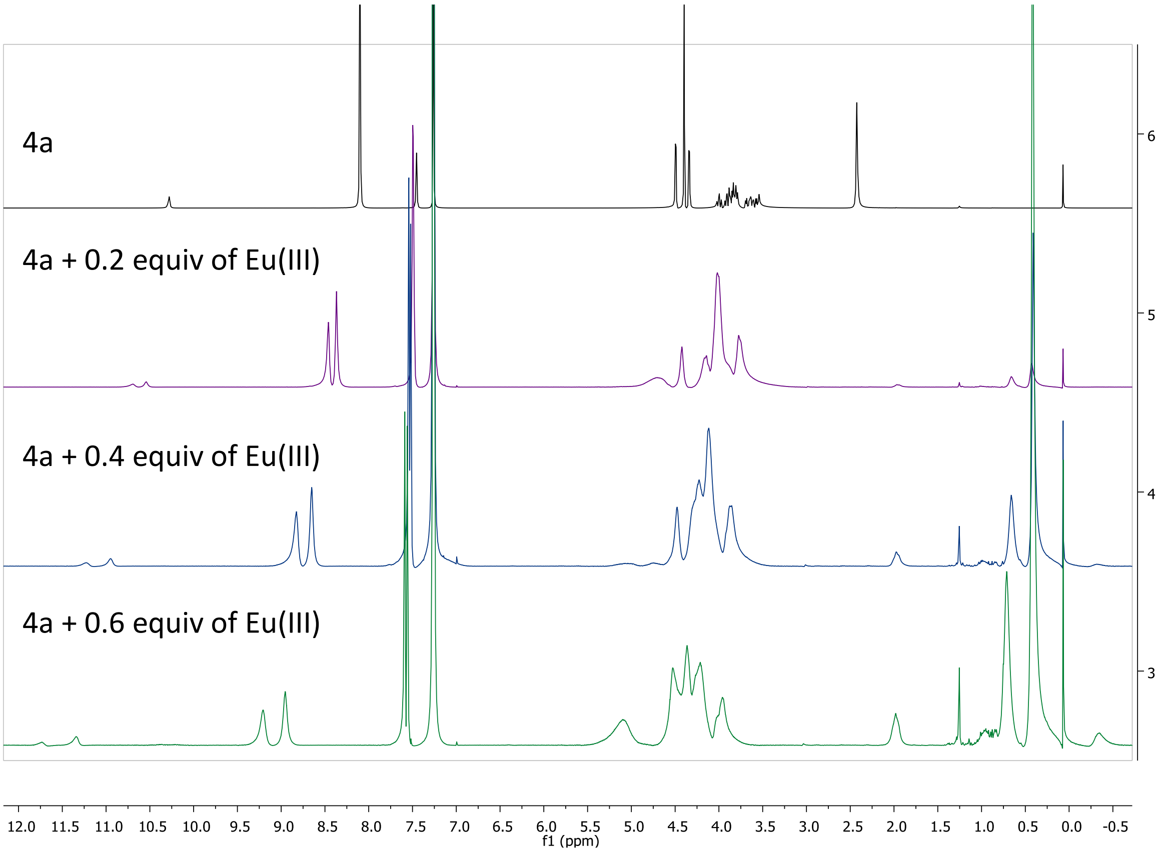

Figure S3

[1] Eu(hfc)$_3$ = Europium tris[3-(heptafluoropropylhydroxymethylene)-(−)-camphorate]

5. CSP-HPLC traces for 4a

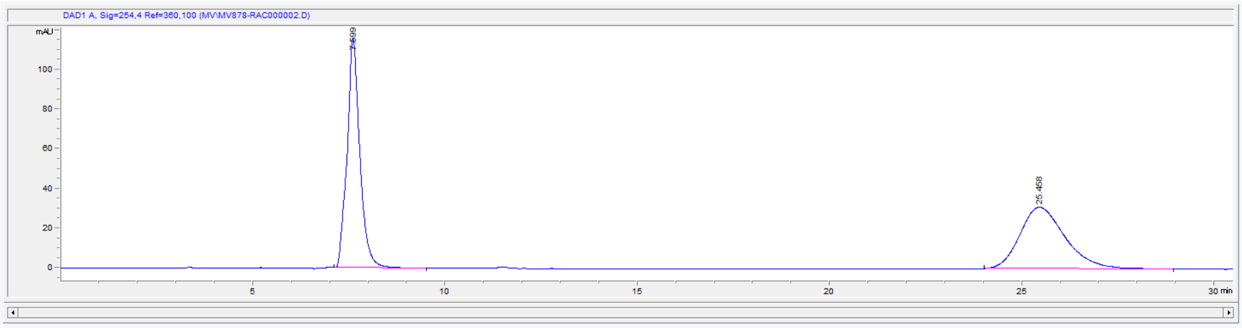

File Information	
LC-File	MV878-RAC000002.D
File Path	C:\CHEM32\1\DATA\MV\
Date	01-Dec-14, 14:35:43
Sample	MV878
Sample Info	IC, Hexane:iPA 85:15, 0.5 mL/min, 23 °C
Barcode	
Operator	mv
Method	MV_IC.M
Analysis Time	30.5 min
Sampling Rate	0.0067 min (0.402 sec), 4576 datapoints

#	Time	Area	Height	Width	Area%	Symmetry
1	7.599	2662	115.4	0.3291	51.239	0.809
2	25.458	2533.2	31	1.0816	48.761	0.728

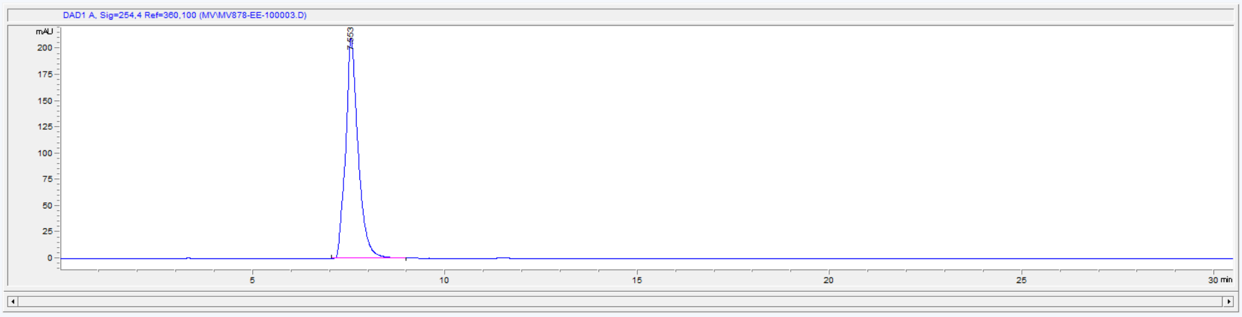

File Information	
LC-File	MV878-EE-100003.D
File Path	C:\CHEM32\1\DATA\MV\
Date	01-Dec-14, 15:10:19
Sample	MV878-ee1
Sample Info	IC, Hexane:iPA 85:15, 0.5 mL/min, 23 °C
Barcode	
Operator	mv
Method	MV_IC.M
Analysis Time	30.493 min
Sampling Rate	0.0067 min (0.402 sec), 4575 datapoints

#	Time	Area	Height	Width	Area%	Symmetry
1	7.553	4739	209.9	0.3194	100.000	0.725

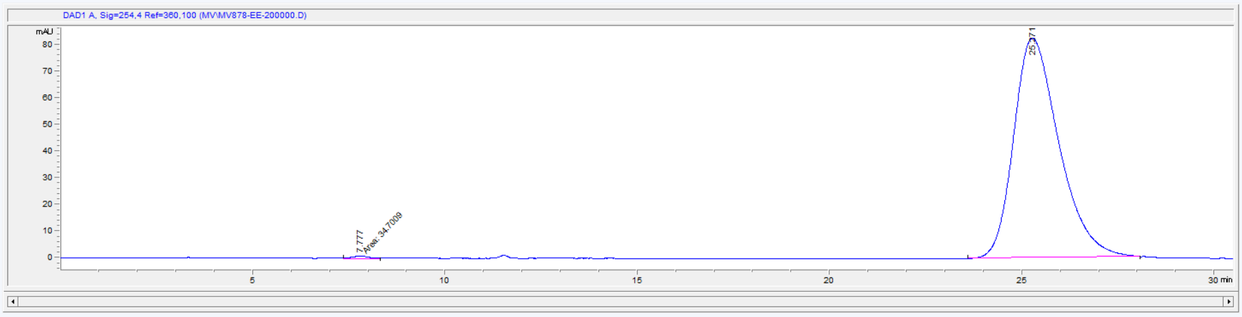

File Information	
LC-File	MV878-EE-200000.D
File Path	C:\CHEM32\1\DATA\MV\
Date	01-Dec-14, 15:47:09
Sample	MV878-ee2
Sample Info	IC, Hexane:iPA 85:15,1.0 mL/min, 23 °C
Barcode	
Operator	mv
Method	MV_IC.M
Analysis Time	30.493 min
Sampling Rate	0.0067 min (0.402 sec), 4575 datapoints

#	Time	Area	Height	Width	Area%	Symmetry
1	7.777	34.7	1	0.559	0.511	0.796
2	25.271	6754	82.5	1.2295	99.489	0.666

HPLC Conditions: IC column; IPA/n-Hexane:15/85; 1.0 mL/min; 23 °C, 254 nm

6. ECD spectra of 4a

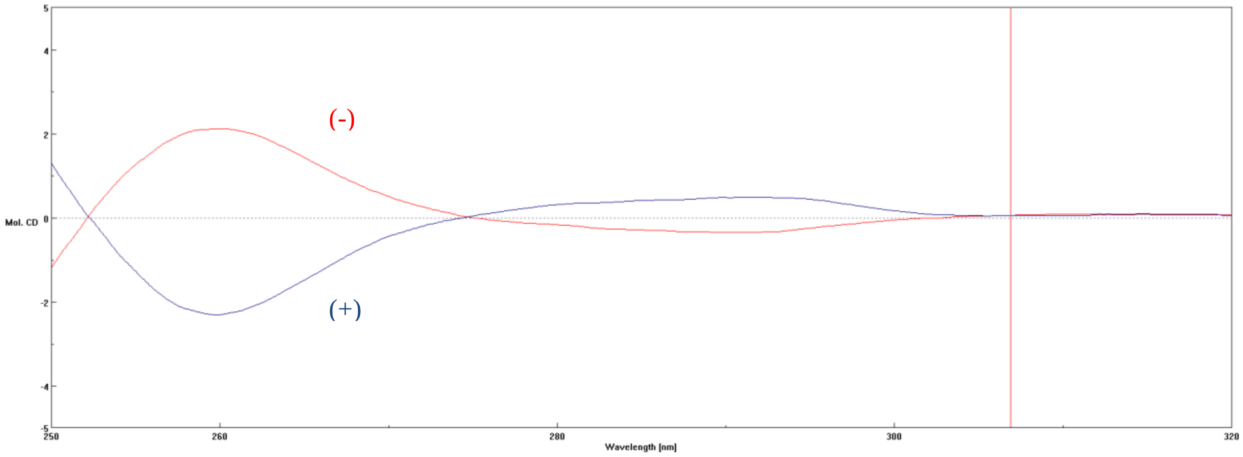

Figure S4

ECD spectra of 10^{-3}M solution of first eluted (–)-**4a** (red) and second eluted (+)-**4a** (blue) in $CHCl_3$

$[\alpha]_D^{20}$ (first eluted **4a**) = –45.4 (*c* 0.26, $CHCl_3$)

$[\alpha]_D^{20}$ (second eluted **4a**) = +48.4 (*c* 0.26, $CHCl_3$)

7. Computational Details.

Geometry optimizations have been performed with the Gaussian 09 (1) package at the B3PW91 (2) level of hybrid density functional theory. The potassium atom was represented by the relativistic effective core potential (RECP) from the Stuttgart group and the associated basis sets (3) augmented by a d polarization function (4). The remaining atoms (H, C, O, N) were represented by a 6-31G** basis set (5). All energies reported in the present work are Zero-point energies corrected.

Table S1 – Relative energies for the trapping of the potassium cation by intermediate **E** (racemic).

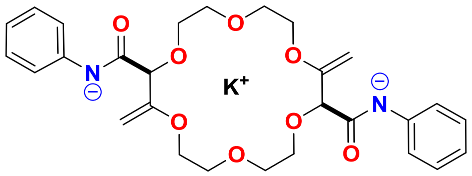

chiral product

	$E_{(Z\text{-point})}$ (kcal/mol)
K^+ trapped by both carbonyl's (**E$_O$**)	0.00
K^+ trapped by one carbonyl and one N^- (**E$_{ON}$**)	+2.38
K^+ trapped by both N^-'s (**E$_N$**)	+5.92

Table S2 – Relative energies for the trapping of the potassium cation by intermediate **F** (meso).

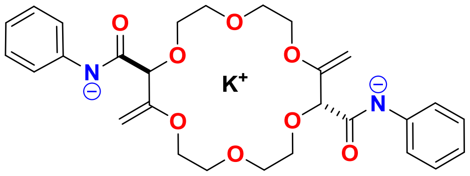

meso product

	$E_{(Z\text{-point})}$ (kcal/mol)
K^+ trapped by the carbonyl (**F$_O$**)	0.00
K^+ trapped by the N^- (**F$_N$**)	+1.72

Table S3 – Relative energies for the trapping of the potassium cation by C18-phenylamide product (racemic).

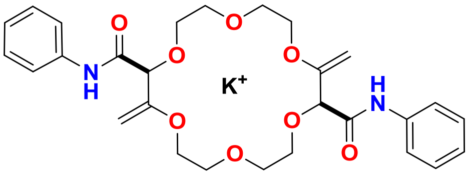

chiral product

	$E_{(Z\text{-point})}$ (kcal/mol)
K^+ trapped by both carbonyl's	0.00
K^+ trapped by one carbonyl and one NH	+4.92
K^+ trapped by both NH's	+10.43

Table S4 – Relative energies for the trapping of the potassium cation by C18-phenylamide product (meso).

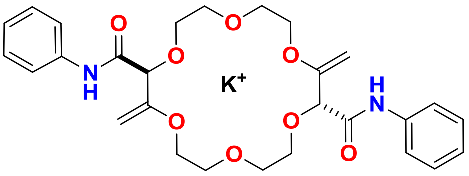

meso product

	$E_{(Z\text{-point})}$ (kcal/mol)
K^+ trapped by the carbonyl on the centre	0.00
K^+ trapped by the carbonyl on the side	+15.36

Table S5 – Relative energies for the most stable conformers for the trapping of the potassium cation by C18-phenylamide product.

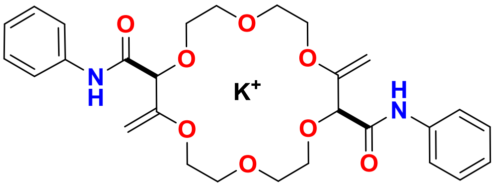

chiral product

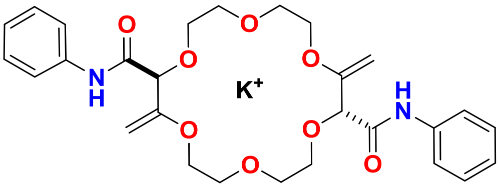

meso product

**$E_{(Z\text{-point})}$ (kcal/mol)**	
0.0	+14.55

Computed Energies and Cartesian Coordinates

Intermediate E_O

B3PW91 Energy = -1825.26668
Z-point correction = 0.569417
Enthalpy correction = 0.608113
Gibbs correction = 0.494706

O	3.925000	7.833000	0.155000
O	4.330000	5.007000	0.032000
O	5.816000	7.660000	4.615000
O	3.517000	5.966000	4.708000
O	5.995000	3.569000	1.540000
O	5.208000	9.155000	2.269000
O	3.170000	4.255000	2.517000
O	7.780000	7.577000	2.812000
N	4.958000	1.603000	0.738000
N	8.561000	9.654000	3.625000
C	5.114000	2.898000	0.946000
C	2.761000	3.716000	1.335000
C	9.773000	9.711000	2.959000
C	5.853000	0.666000	1.225000
C	3.970000	7.088000	-1.045000
H	5.005000	7.014000	-1.415000
H	3.365000	7.580000	-1.826000
C	10.529000	10.893000	3.148000
H	10.099000	11.655000	3.794000
C	3.906000	3.679000	0.353000
H	3.571000	3.159000	-0.552000
C	7.726000	8.641000	3.478000
C	7.873000	-0.140000	2.340000
C	7.069000	0.913000	1.912000
H	7.354000	1.943000	2.091000
C	3.016000	4.657000	4.842000
H	2.333000	4.583000	5.707000
H	3.838000	3.939000	4.999000
C	5.515000	-0.690000	1.001000
H	4.586000	-0.885000	0.471000
C	4.409000	9.847000	1.335000
H	4.783000	10.872000	1.198000
H	3.368000	9.901000	1.684000
C	6.419000	8.913000	4.277000
H	6.680000	9.465000	5.188000
C	5.464000	9.754000	3.465000
C	2.258000	4.278000	3.592000
H	1.810000	3.284000	3.734000
H	1.450000	4.998000	3.396000
C	4.237000	6.392000	5.847000
H	5.055000	5.689000	6.071000
H	3.574000	6.432000	6.729000
C	3.426000	5.701000	-0.790000
H	2.430000	5.774000	-0.327000
H	3.304000	5.186000	-1.759000
C	4.809000	7.766000	5.590000
H	4.006000	8.447000	5.270000
H	5.221000	8.160000	6.536000
C	7.517000	-1.470000	2.110000
H	8.155000	-2.283000	2.449000
C	4.491000	9.116000	0.016000
H	3.950000	9.702000	-0.748000
H	5.545000	9.045000	-0.296000
C	4.941000	10.918000	3.878000
H	4.272000	11.525000	3.279000
H	5.233000	11.307000	4.846000
C	6.324000	-1.733000	1.433000
C	10.341000	8.720000	2.119000
H	9.786000	7.803000	1.964000
C	11.763000	11.084000	2.542000
C	11.581000	8.921000	1.516000
C	1.519000	3.287000	1.067000
H	0.709000	3.300000	1.786000
H	1.313000	2.859000	0.093000
C	12.306000	10.096000	1.715000
H	13.274000	10.240000	1.238000
H	8.802000	0.086000	2.863000
H	6.023000	-2.762000	1.238000
H	11.988000	8.138000	0.878000
H	12.310000	12.010000	2.715000
K	5.668000	6.085000	2.277000

Intermediate E_{ON}

B3PW91 Energy = -1825.263293
Z-point correction = 0.569816
Enthalpy correction = 0.608362
Gibbs correction = 0.496298

O	3.925426	7.833178	0.154694
O	4.330412	5.007280	0.032397
O	5.815872	7.659935	4.614604
O	3.516829	5.966326	4.708373
O	5.995213	3.569051	1.540064
O	5.207594	9.155188	2.268669
O	3.170145	4.255105	2.517246
O	7.779865	7.576672	2.812077
N	4.958359	1.602982	0.738113
N	8.560868	9.653630	3.624823
C	5.114468	2.897777	0.946385
C	2.760614	3.716253	1.335224
C	9.773152	9.710870	2.959159
C	5.853172	0.666092	1.225415
C	3.969791	7.087842	-1.044624
H	5.004869	7.013917	-1.415484

H	3.364504	7.579539	-1.826404
C	10.528785	10.892643	3.148258
H	10.099194	11.654694	3.794133
C	3.906407	3.678591	0.353144
H	3.571228	3.159401	-0.552230
C	7.725861	8.640988	3.477739
C	7.873329	-0.140087	2.339766
C	7.068551	0.913467	1.912076
H	7.353639	1.942852	2.090829
C	3.016484	4.656519	4.841831
H	2.333194	4.583459	5.706564
H	3.837682	3.939343	4.999297
C	5.514943	-0.689891	1.000943
H	4.585525	-0.884987	0.471246
C	4.409399	9.847289	1.334883
H	4.783321	10.872303	1.198348
H	3.367752	9.900974	1.684171
C	6.418951	8.913220	4.277062
H	6.679620	9.465162	5.187687
C	5.463547	9.753867	3.465094
C	2.257642	4.277720	3.591750
H	1.809725	3.283621	3.733711
H	1.450014	4.998338	3.395953
C	4.236885	6.391607	5.846956
H	5.055498	5.688845	6.071287
H	3.574029	6.431585	6.729195
C	3.426204	5.701387	-0.789977
H	2.430237	5.774187	-0.326526
H	3.303668	5.185903	-1.758703
C	4.809224	7.766106	5.589858
H	4.005639	8.447376	5.270105
H	5.220892	8.160219	6.535588
C	7.517108	-1.469840	2.110435
H	8.155313	-2.282978	2.449266
C	4.490733	9.115652	0.015934
H	3.950453	9.702230	-0.748295
H	5.544912	9.045432	-0.296086
C	4.941455	10.917798	3.878061
H	4.272467	11.524712	3.278771
H	5.232589	11.307133	4.845778
C	6.323534	-1.733145	1.432633
C	10.341166	8.719890	2.119478
H	9.785536	7.803411	1.963836
C	11.763321	11.083742	2.541616
C	11.580809	8.920786	1.516475
C	1.519069	3.286915	1.066684
H	0.708512	3.299753	1.786368
H	1.313014	2.858836	0.093496
C	12.306195	10.096473	1.714589
H	13.273574	10.240168	1.238299
H	8.801780	0.086008	2.862897
H	6.022929	-2.761949	1.238283
H	11.988472	8.137531	0.878428
H	12.309709	12.010025	2.714960
K	5.667566	6.084585	2.276523

Intermediate E_N

B3PW91 Energy = -1825.257474
Z-point correction = 0.569639
Enthalpy correction = 0.608306
Gibbs correction = 0.495413

O	0.128057	-0.244896	-0.042586
O	-0.121051	-0.385293	2.806545
O	4.936389	-0.207423	0.793284
O	3.936706	2.191748	1.996919
O	0.338077	0.314901	6.262636
O	2.753752	-0.431076	-0.974817
O	1.467449	1.909253	3.268599
O	5.195146	-3.135188	-1.191204
N	1.775326	-0.806182	4.760209
N	3.858483	-2.758490	0.719483
C	0.721696	-0.092957	5.145769
C	0.169660	1.845780	3.671078
C	3.420272	-4.066186	0.900080
C	2.720006	-1.232826	5.688009
C	-0.936583	-0.889106	0.628288
H	-0.686624	-1.943365	0.826680
H	-1.851606	-0.867977	0.010562
C	2.562411	-4.309786	2.000537
H	2.319905	-3.487313	2.668988
C	-0.194079	0.412045	3.991341
H	-1.217741	0.394148	4.386893
C	4.672446	-2.450472	-0.286557
C	4.802904	-2.490634	6.022593
C	3.783771	-2.028957	5.198197
H	3.789597	-2.303318	4.146240
C	3.583486	2.934244	3.141303
H	4.102884	3.908949	3.151843
H	3.857367	2.390682	4.058601
C	2.743994	-0.933438	7.074808
H	1.935742	-0.335603	7.476183
C	1.676566	-0.189339	-1.853118
H	1.885749	-0.624894	-2.840079
H	1.507802	0.890632	-1.974733
C	4.988991	-0.932190	-0.438676
H	6.002416	-0.877550	-0.856002
C	4.027790	-0.326459	-1.438699
C	2.089818	3.168394	3.141296
H	1.826824	3.811301	3.993079
H	1.772214	3.667446	2.214076

C	5.301905	1.825152	1.979870
H	5.549808	1.216647	2.864034
H	5.943442	2.723652	1.996257
C	-1.202489	-0.169502	1.929942
H	-1.345365	0.903017	1.727852
H	-2.139293	-0.556377	2.367348
C	5.582971	1.042172	0.718838
H	5.234133	1.620402	-0.150367
H	6.673933	0.908348	0.616650
C	4.812184	-2.178660	7.383690
H	5.607447	-2.539481	8.032228
C	0.456064	-0.861877	-1.266404
H	-0.380881	-0.781175	-1.982771
H	0.677619	-1.929746	-1.117257
C	4.386537	0.216679	-2.610025
H	3.677407	0.624437	-3.322186
H	5.432894	0.221907	-2.889301
C	3.772077	-1.399157	7.890846
C	3.725440	-5.184779	0.081765
H	4.384733	-5.027627	-0.762160
C	2.039823	-5.569541	2.267855
C	3.193938	-6.441950	0.357841
C	-0.664186	2.889376	3.775080
H	-0.375626	3.911251	3.554542
H	-1.672603	2.722556	4.133057
C	2.347230	-6.655632	1.445937
H	1.941339	-7.643334	1.652939
H	5.592781	-3.105600	5.595334
H	3.755290	-1.146947	8.950930
H	3.452410	-7.275112	-0.295360
H	1.391758	-5.701714	3.132273
K	2.510123	-0.631741	2.024738

Intermediate F_{O}

B3PW91 Energy = -1825.25843

Z-point correction = 0.5697

Enthalpy correction = 0.608155

Gibbs correction = 0.495757

O	-0.904502	0.362823	0.151659
C	-0.569823	-0.297692	1.300256
C	0.922902	-0.565444	1.370296
O	1.681073	0.410407	0.663801
C	1.634462	1.712641	1.193413
C	2.552469	2.581976	0.360973
O	2.082683	2.612680	-0.970284
C	2.939702	3.324404	-1.844389
C	2.512443	3.108648	-3.275208
H	1.451883	3.341719	-3.430594
H	3.097940	3.773739	-3.924251
H	3.978962	2.981074	-1.713983
H	2.907092	4.403530	-1.621213
H	2.583000	3.598372	0.789727
H	3.573920	2.169508	0.393223
H	1.980894	1.730349	2.240500
H	0.611667	2.116945	1.167623
C	1.235741	-1.949484	0.731376
N	1.403602	-2.868256	1.661554
O	1.248829	-1.993279	-0.529165
C	-1.429384	-0.723020	2.234205
H	-1.033482	-1.253916	3.091211
H	-2.505833	-0.640079	2.147442
O	2.791557	1.743030	-3.612652
C	2.522795	1.361873	-4.912276
C	1.123418	1.592875	-5.433562
O	0.239434	1.218068	-4.378888
C	-1.128110	1.174286	-4.739973
C	-1.952015	1.721419	-3.601304
O	-1.814723	0.888111	-2.455624
C	-2.416949	1.458340	-1.318953
C	-2.279558	0.532157	-0.132961
H	-2.740350	-0.443846	-0.337466
H	-2.795451	0.985962	0.725809
H	-1.954148	2.430818	-1.083678
H	-3.492409	1.638422	-1.492908
H	-3.012878	1.776218	-3.898816
H	-1.587487	2.736334	-3.394685
H	-1.317629	1.809097	-5.611416
H	-1.421571	0.137346	-4.971685
C	0.932504	3.033351	-6.029963
N	0.137185	3.819706	-5.309421
O	1.557385	3.206913	-7.094684
C	3.483315	0.750285	-5.607482
H	3.280618	0.356815	-6.595948
H	4.485407	0.655869	-5.202355
H	1.227311	-0.602723	2.422044
H	0.990411	0.920690	-6.293398
C	-0.145802	5.118019	-5.702374
C	-0.963982	5.877009	-4.831374
C	-1.323352	7.189278	-5.110995
C	-0.881580	7.810668	-6.281746
C	-0.072931	7.082188	-7.155484
C	0.295060	5.766802	-6.884094
H	-1.306082	5.394063	-3.918570
H	-1.954055	7.733781	-4.409593
H	-1.160378	8.837843	-6.505655
H	0.283413	7.548755	-8.073285
H	0.927549	5.206841	-7.561792
C	1.625610	-4.200646	1.349493
C	1.742201	-5.080940	2.450571
C	1.968336	-6.441220	2.286048
C	2.090634	-6.987929	1.005797

C	1.980542	-6.138146	-0.095465
C	1.753440	-4.772411	0.059620
H	1.646630	-4.645849	3.442436
H	2.051115	-7.082392	3.162479
H	2.268919	-8.052415	0.870829
H	2.074629	-6.545975	-1.101274
H	1.671200	-4.117047	-0.798473
K	1.015561	0.164418	-1.981634

Intermediate F_N

B3PW91 Energy = -1825.255618
Z-point correction = 0.569636
Enthalpy correction = 0.608106
Gibbs correction = 0.49564

O	-0.854992	0.367806	0.041114
C	-0.499765	-0.330654	1.161024
C	1.001229	-0.560114	1.221008
O	1.732442	0.431571	0.517240
C	1.683327	1.720252	1.083913
C	2.585256	2.621003	0.268477
O	2.093812	2.695779	-1.052651
C	2.931098	3.444710	-1.915178
C	2.490220	3.262232	-3.346289
H	1.424446	3.483448	-3.481913
H	3.057796	3.953650	-3.983697
H	3.976247	3.112365	-1.806204
H	2.885452	4.516263	-1.660393
H	2.618413	3.621852	0.732417
H	3.608358	2.211604	0.270465
H	2.041906	1.707617	2.127118
H	0.657623	2.117839	1.082637
C	1.318082	-1.994402	0.687063
N	1.536481	-2.047059	-0.626033
O	1.279829	-2.871324	1.571512
C	-1.344402	-0.819632	2.077043
H	-0.929816	-1.377338	2.907534
H	-2.423110	-0.767945	1.992757
O	2.785228	1.910857	-3.722758
C	2.503195	1.558408	-5.027520
C	1.095721	1.791527	-5.525067
O	0.228592	1.377507	-4.471053
C	-1.141183	1.320216	-4.821046
C	-1.963088	1.817814	-3.658647
O	-1.800375	0.952633	-2.540182
C	-2.404554	1.474505	-1.382106
C	-2.236105	0.514039	-0.228092
H	-2.675365	-0.465204	-0.462308
H	-2.755156	0.925812	0.649757
H	-1.961310	2.449287	-1.120014
H	-3.485334	1.636040	-1.541036
H	-3.027704	1.862977	-3.944185
H	-1.613954	2.832021	-3.423837
H	-1.349889	1.978998	-5.670061
H	-1.418815	0.286029	-5.082221
C	0.883714	3.247259	-6.075404
N	0.075414	3.998092	-5.331350
O	1.506116	3.464928	-7.133496
C	3.458670	0.968719	-5.748026
H	3.245800	0.595796	-6.742288
H	4.465891	0.869768	-5.357057
H	1.305881	-0.571646	2.275599
H	0.958616	1.143044	-6.402264
C	-0.224456	5.304662	-5.681060
C	-1.069463	6.016654	-4.795865
C	-1.446539	7.332474	-5.031700
C	-0.996065	8.005341	-6.170289
C	-0.160820	7.324028	-7.057069
C	0.224505	6.005235	-6.829811
H	-1.418471	5.493369	-3.908256
H	-2.098008	7.839416	-4.321140
H	-1.288583	9.035616	-6.359926
H	0.202651	7.831118	-7.950281
H	0.876106	5.480826	-7.517772
C	1.811119	-3.256512	-1.253034
C	2.191790	-3.200869	-2.615300
C	2.462677	-4.343208	-3.358619
C	2.367911	-5.606764	-2.772965
C	2.002047	-5.688020	-1.428722
C	1.730572	-4.549077	-0.675523
H	2.292271	-2.224881	-3.086483
H	2.753975	-4.244125	-4.402789
H	2.578500	-6.504353	-3.349607
H	1.926156	-6.663580	-0.949870
H	1.456951	-4.618926	0.369496
K	1.047006	0.258170	-2.101554

References

(1) Frisch, M. J.; Trucks, G. W.; Schlegel, H. B.; Scuseria, G. E.; Robb, M. A.; Cheeseman, J. R.; Scalmani, G.; Barone, V.; Mennucci, B.; Petersson, G. A.; Nakatsuji, H.; Caricato, M.; Li, X.; Hratchian, H. P.; Izmaylov, A. F.; Bloino, J.; Zheng, G.; Sonnenberg, J. L.; Hada, M.; Ehara, M.; Toyota, K.; Fukuda, R.; Hasegawa, J.; Ishida, M.; Nakajima, T.; Honda, Y.; Kitao, O.; Nakai, H.; Vreven, T.; Montgomery, J. A., Jr.; Peralta, J. E.; Ogliaro, F.; Bearpark, M.; Heyd, J. J.; Brothers, E.; Kudin, K. N.; Staroverov, V. N.; Kobayashi, R.; Normand, J.; Raghavachari, K.; Rendell, A.; Burant, J. C.; Iyengar, S. S.; Tomasi, J.; Cossi, M.; Rega, N.; Millam, J. M.; Klene, M.; Knox, J. E.; Cross, J. B.; Bakken, V.; Adamo, C.; Jaramillo, J.; Gomperts, R.; Stratmann, R. E.; Yazyev, O.; Austin, A. J.; Cammi, R.; Pomelli, C.; Ochterski, J. W.; Martin, R. L.; Morokuma, K.; Zakrzewski, V. G.; Voth, G. A.; Salvador, P.; Dannenberg,J.J.;Dapprich,S.;Daniels,A.D.;Farkas,O.; Foresman, J. B.; Ortiz, J. V.; Cioslowski, J.; Fox, D. J. *Gaussian 09*, Revision D1; Gaussian, Inc.: Wallingford, CT, **2009**.

(2) a) Becke, A. D. *J. Chem. Phys.* **1993**, 98, 5648−5652. b) Perdew, J. P.; Wang, Y. *Phys. Rev. B: Condens. Matter* **1992**, 45, 13244−13249.

(3) Andrae, D.; Haußermann, U.; Dolg, M.; Stoll, H.; Preuß, H. *Theor. Chim. Acta* **1990**, 77, 123−141.

(4) A. Höllwarth, M. Böhme, S. Dapprich, A.W. Ehlers, A. Gobbi, V. Jonas, K.F. Köhler, R. Stegmann, A. Veldkamp and G. Frenking *Chem. Phys. Lett.* **1993**, 208, 237- 240.

(5) a) Hehre, W. J.; Ditchfield, R.; Pople, J. A. *J. Chem. Phys.* **1972**, *56*, 2257-2261. b) Hehre, W. J.; Stewart, R. F.; Pople, J. A. *J. Chem. Phys.* **1969**, *51*, 2657-2664.

8. Vibrational circular dichroism (VCD) and infrared (IR) analysis

IR and VCD measurements

IR and vibrational circular dichroism (VCD) spectra were recorded on a Bruker PMA 50 accessory coupled to a Tensor 27 Fourier transform infrared spectrometer. A photoelastic modulator (Hinds PEM 90) set at l/4 retardation was used to modulate the handedness of the circular polarized light. Demodulation was performed by a lock-in amplifier (SR830 DSP). An optical low-pass filter (< 1800 cm-1) in front of the photoelastic modulator was used to enhance the signal/noise ratio. Spectra were recorded with a transmission cell equipped with CaF_2 windows and a 0.2 mm Teflon spacer. Solutions of (–)-**4a** and (+)-**4a** in CD_2Cl_2 at concentrations of 7 mg in 700 µl CD_2Cl_2 were measured under identical conditions and subtracted to each other in order to eliminate artifacts. Samples were measured at a resolution of 4 cm^{-1} by averaging about 24'000 scans for both enantiomers. Spectra are presented without further data processing.

IR and VCD calculations

Calculations were performed for (*R*,*R*)-**4a**. The geometry optimizations, vibrational frequencies, IR absorption and VCD intensities were calculated with Density Functional Theory (DFT) using the B3PW91 functional and a 6-31+G(d,p) basis set. Frequencies were scaled by a factor of 0.98. IR absorption and VCD spectra were constructed from calculated dipole and rotational strengths assuming Lorentzian band shape with a half-width at half maximum of 4 cm^{-1}. The crystal structure served as the starting point for the geometry optimization, which was done for the molecule and its complex with water. All calculations were performed using Gaussian09.[1]

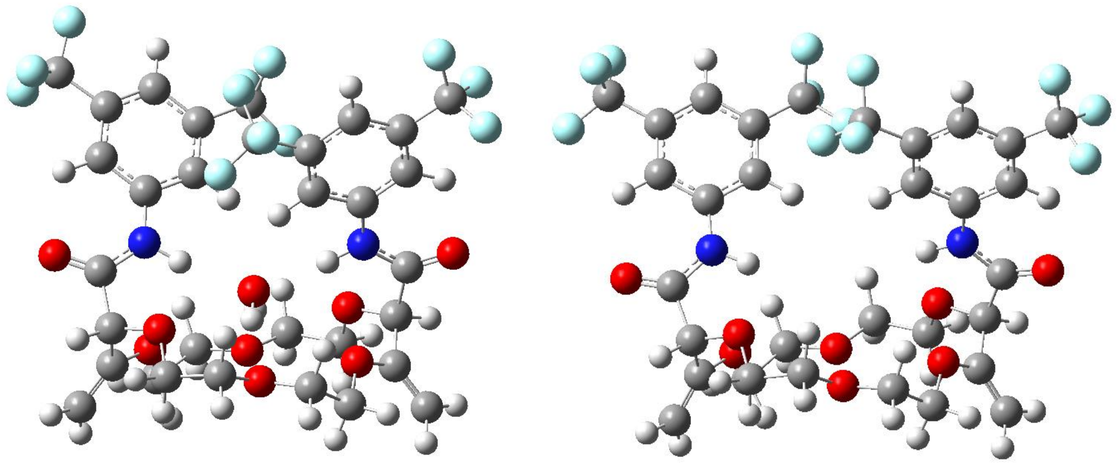

Figure S5: Optimized structures of (R,R)-**4a**·H_2O (left) and (R,R)-**4a** (right).

The IR spectrum clearly shows the coexistence of **4a** and its complex with water (**4a**·H_2O) in solution. This is evidenced by the double band between 1550 cm^{-1} and 1580 cm^{-1} due to the N-H deformation

vibration of the amide bond, which shifts to higher wavenumbers upon complex formation (see IR Figure).

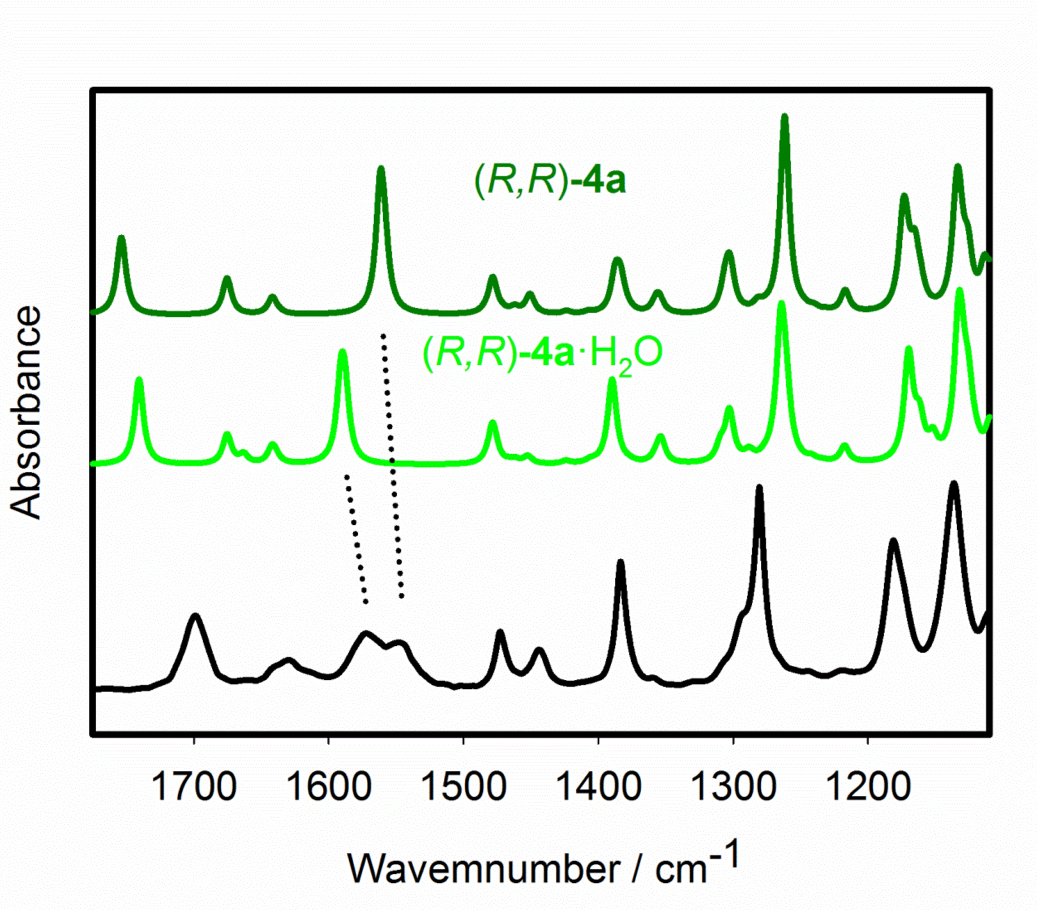

Figure S6: IR spectra of **4a**. Bottom: experimental spectrum (7 mg in 700 ml CD_2Cl_2), top: calculated spectra for **4a** and its complex with water. Dashed lines indicate the N-H vibrations that are sensitive to complex formation.

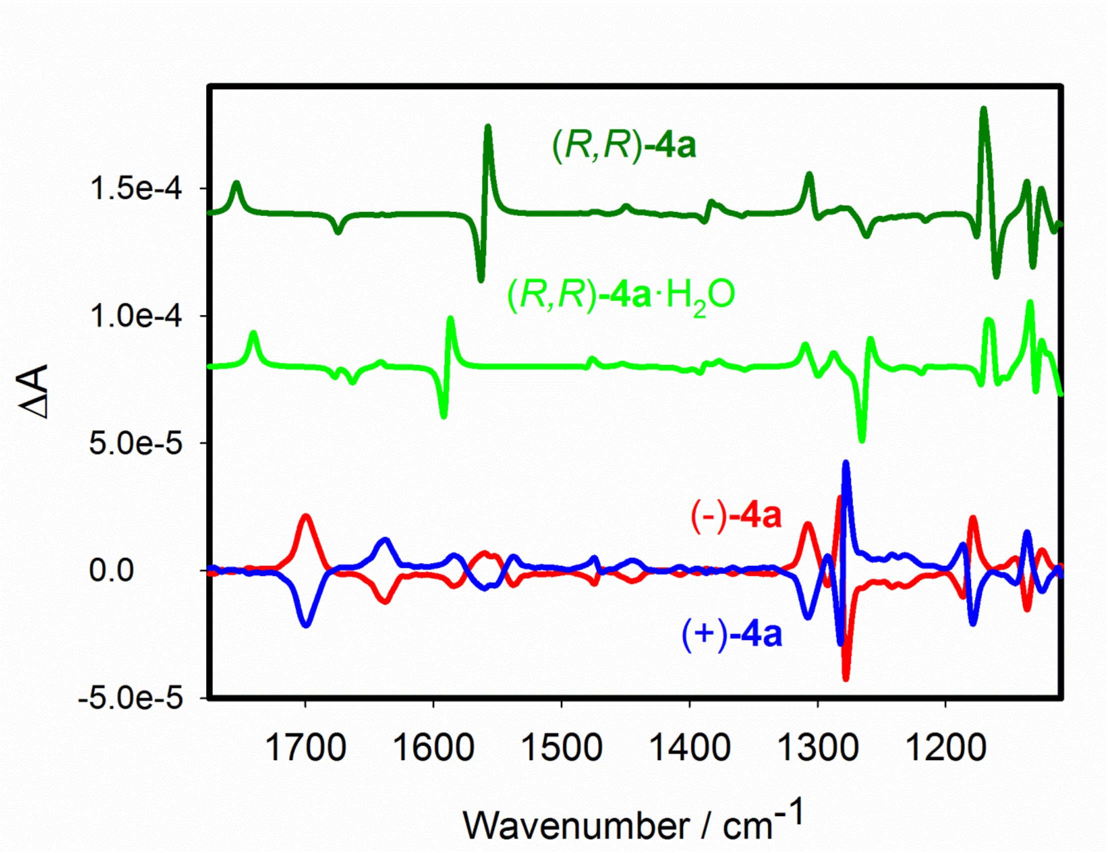

Figure S7: VCD spectra of **4a**. Bottom: experimental spectra (7 mg in 700 ml CD_2Cl_2) of (-)-**4a** and (+)-**4a**, top: calculated spectra for the (*R*,*R*)-**4a** and (*R*,*R*)-**4a**·H_2O.

(1) Gaussian 09, Revision C.01, M. J. Frisch, G. W. Trucks, H. B. Schlegel, G. E. Scuseria, M. A. Robb, J. R. Cheeseman, G. Scalmani, V. Barone, B. Mennucci, G. A. Petersson, H. Nakatsuji, M. Caricato, X. Li, H. P. Hratchian, A. F. Izmaylov, J. Bloino, G. Zheng, J. L. Sonnenberg, M. Hada, M. Ehara, K. Toyota, R. Fukuda, J. Hasegawa, M. Ishida, T. Nakajima, Y. Honda, O. Kitao, H. Nakai, T. Vreven, J. A. Montgomery, Jr., J. E. Peralta, F. Ogliaro, M. Bearpark, J. J. Heyd, E. Brothers, K. N. Kudin, V. N. Staroverov, T. Keith, R. Kobayashi, J. Normand, K. Raghavachari, A. Rendell, J. C. Burant, S. S. Iyengar, J. Tomasi, M. Cossi, N. Rega, J. M. Millam, M. Klene, J. E. Knox, J. B. Cross, V. Bakken, C. Adamo, J. Jaramillo, R. Gomperts, R. E. Stratmann, O. Yazyev, A. J. Austin, R. Cammi, C. Pomelli, J. W. Ochterski, R. L. Martin, K. Morokuma, V. G. Zakrzewski, G. A. Voth, P. Salvador, J. J. Dannenberg, S. Dapprich, A. D. Daniels, O. Farkas, J. B. Foresman, J. V. Ortiz, J. Cioslowski, and D. J. Fox, Gaussian, Inc., Wallingford CT, 2010.

9. Crystallographic data

All data were collected on an Agilent Supernova diffractometer equipped with an ATLAS CCD detector using Cu radiation. Integration and data reduction were carried out in the crysalis Software[1] [ref1]. Refinement was made using SHELXL[2]. Material for publication was prepared using the Olex2[3] software.

Macrocycle 4a

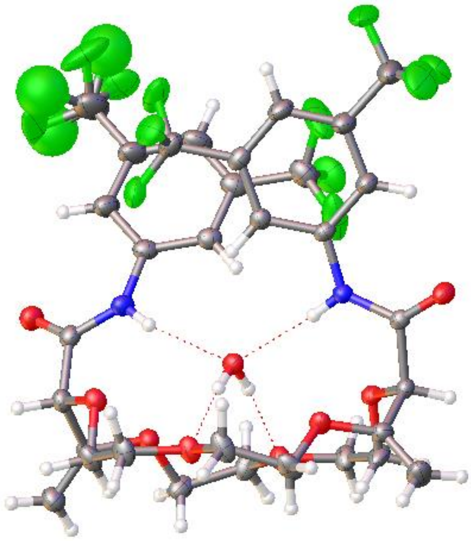

Figure S5

**CCDC number**	**1034977**
Formula	$C_{32}H_{32}F_{12}N_2O_9$
$D_{calc.}$/ g cm^{-3}	1.467
m/mm^{-1}	0.145
Formula Weight	816.60
Colour	orange
Shape	block
Max Size/mm	0.27
Mid Size/mm	0.21
Min Size/mm	0.12
T/K	119.9(3)
Crystal System	monoclinic
Space Group	$P2_1/c$
a/Å	11.9175(4)
b/Å	19.0210(5)
c/Å	17.2260(6)
a/°	90
b/°	108.787(4)
g/°	90
V/Å^3	3696.8(2)
Z	4
Z'	1
θ_{min}/°	3.29
θ_{max}/°	28.22
Measured Refl.	17106
Independent Refl.	7663
Reflections Used	5916
R_{int}	0.0267
Parameters	519
Restraints	36
Largest Peak	0.616
Deepest Hole	-0.450
GooF	1.022
wR_2 (all data)	0.1330
wR_2	0.1211
R_1 (all data)	0.0679
R_1	0.0505

Comments on the X-ray structure:

One of the CF_3 group is disordered and was refined using two components, the one with the smaller occupancy was refined isotropically. Geometrical restrains (1-2 and 1-3 bond lengths) were applied as well as restraints on the anisotropic displacement parameters.

Macrocycle 4l

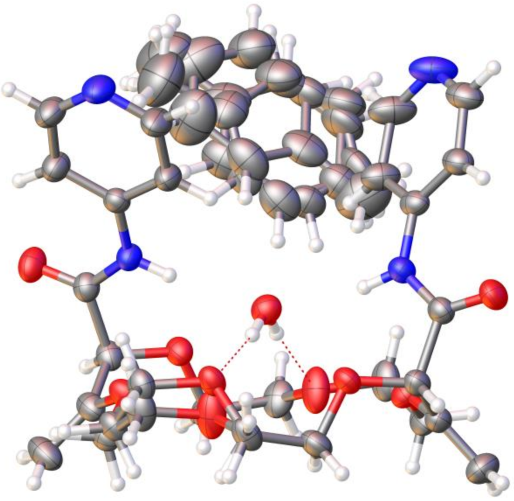

Figure S6

**CCDC number**	**1034976**
Formula	$C_{33}H_{42}N_4O_9$
$D_{calc.}$/ g cm^{-3}	1.241
m/mm^{-1}	0.751
Formula Weight	638.70
Colour	colourless
Shape	plate
Max Size/mm	0.81
Mid Size/mm	0.42
Min Size/mm	0.13
T/K	180(2)
Crystal System	monoclinic
Space Group	$P2_1/n$
a/Å	10.43180(15)
b/Å	18.5656(3)
c/Å	17.6555(3)
a/°	90
b/°	90.8170(13)
g/°	90
V/Å^3	3419.05(8)
Z	4
Z'	1
θ_{min}/°	3.455
θ_{max}/°	72.537
Measured Refl.	13891
Independent Refl.	6622
Reflections Used	5819
R_{int}	0.0162
Parameters	521
Restraints	299
Largest Peak	0.228
Deepest Hole	-0.170
GooF	1.106
wR_2 (all data)	0.1370
wR_2	0.1295
R_1 (all data)	0.0426
R_1	0.0377

Comments on the X-ray structure:

Part of the macrocycle is disordered and was refined using two components. Geometrical restraints were applied on 1-2 and 1-3 distances as well as restraints on anisotropic displacement parameters.

A disordered toluene molecule is also present. It was modelled using three rigid bodies. Restraints were applied on anisotropic displacement parameters.
The water molecule was refined as a rigid-body.

Macrocycle 8a

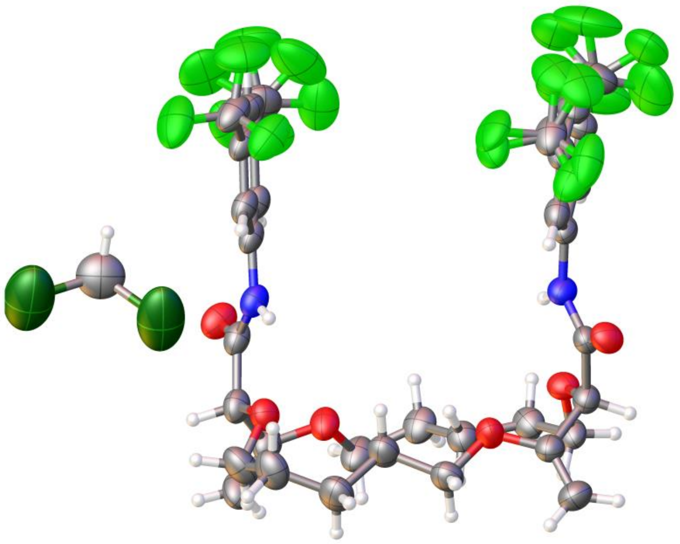

Figure S7

**CCDC number**	**1034974**
Formula	$C_{35}H_{36}Cl_2F_{12}N_2O_6$
$D_{calc.}$/ g cm^{-3}	1.436
μ/mm^{-1}	2.340
Formula Weight	879.56
Colour	colourless
Shape	block
Max Size/mm	0.43
Mid Size/mm	0.19
Min Size/mm	0.15
T/K	180.1(5)
Crystal System	monoclinic
Space Group	C2/c
a/Å	29.3901(7)
b/Å	13.5696(4)
c/Å	21.1866(6)
α/°	90
β/°	105.652(3)
γ/°	90
V/Å^3	8136.2(4)
Z	8
Z'	1
θ_{min}/°	3.123
θ_{max}/°	72.571
Measured Refl.	15971
Independent Refl.	7836
Reflections Used	6433
R_{int}	0.0308
Parameters	598
Restraints	171
Largest Peak	1.522
Deepest Hole	-0.984
GooF	1.092
wR_2 (all data)	0.2851
wR_2	0.2596
R_1 (all data)	0.0968
R_1	0.0848

Comments on the X-ray structure:

Three disordered CF_3 groups were refined as two components each. Geometrical restraints on C-F and F-F distances were applied, as well as restraints on anisotropic displacement parameters.

There is still a small peak present close to the dichloromethane molecule that may be due to a small disorder in the solvent model. Inclusion of this disorder in the model did not improve it significantly, so that this disorder was not included in the final model.

Macrocycle 10o

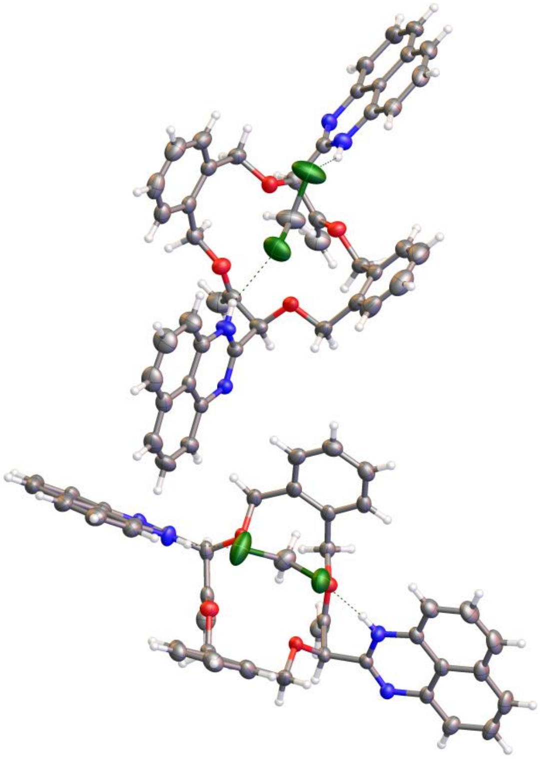

Figure S8

**CCDC number**	**1034978**
Formula	$C_{45}H_{38}Cl_2N_4O_4$
$D_{calc.}$/ g cm^{-3}	1.318
μ/mm^{-1}	1.904
Formula Weight	769.69
Colour	yellow
Shape	prism
Max Size/mm	0.28
Mid Size/mm	0.07
Min Size/mm	0.05
T/K	180(2)
Crystal System	Triclinic
Space Group	P-1
a/Å	9.5715(3)
b/Å	18.5858(4)
c/Å	23.8686(8)
α/°	67.485(3)
β/°	81.597(3)
γ/°	88.411(2)
V/Å^3	3878.5(2)
Z	4
Z'	2
θ_{min}/°	3.84
θ_{max}/°	72.55
Measured Refl.	27896
Independent Refl.	14967
Reflections Used	11614
R_{int}	0.0271
Parameters	1003
Restraints	0
Largest Peak	1.277
Deepest Hole	-1.022
GooF	1.011
wR_2 (all data)	0.1496
wR_2	0.1355
R_1 (all data)	0.0710
R_1	0.0540

10. References

1 *CrysalisPro Software system*, Agilent Technologies UK Ltd., Oxford, UK.

2 G. M. Sheldrick, *Acta Crystallogr. A*, 2008, **64**, 112–122.

3 O. V. Dolomanov, L. J. Bourhis, R. J. Gildea, J. A. K. Howard and H. Puschmann, *J. Appl. Crystallogr.*, 2009, **42**, 339–341.

4 P. van der Sluis and A. L. Spek, *Acta Crystallogr. A*, 1990, **46**, 194–201.

5 A. L. Spek, *J. Appl. Crystallogr.*, 2003, **36**, 7–13.
